# Synthesis and Properties of Oligonucleotides Containing LNA-Sulfamate and Sulfamide Backbone Linkages

**DOI:** 10.1021/acs.orglett.4c01232

**Published:** 2024-05-08

**Authors:** Belma Zengin Kurt, Debashis Dhara, Afaf H. El-Sagheer, Tom Brown

**Affiliations:** † Department of Chemistry, Chemistry Research Laboratory, University of Oxford, Oxford OX1 3TA, U.K.; ∥ Department of Pharmaceutical Chemistry, Faculty of Pharmacy, Bezmialem Vakif University, 34093 Istanbul, Turkey; § School of Chemistry, University of Southampton, Highfield, Southampton SO17 1BJ, U.K.

## Abstract

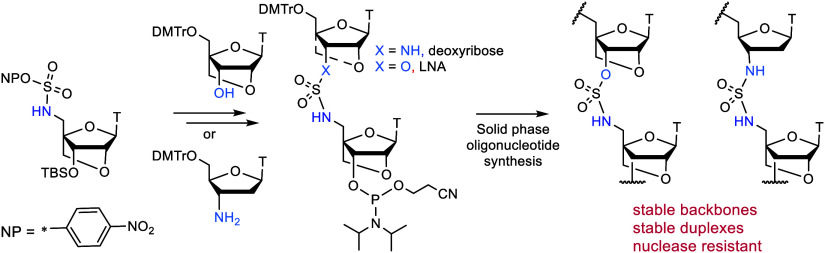

Oligonucleotides hold great promise as therapeutic agents but poor bioavailability limits their utility. Hence, new analogues with improved cell uptake are urgently needed. Here, we report the synthesis and physical study of reduced-charge oligonucleotides containing artificial LNA-sulfamate and sulfamide linkages combined with 2′-O-methyl sugars and phosphorothioate backbones. These oligonucleotides have high affinity for RNA and excellent nuclease resistance.

Therapeutic oligonucleotides have great potential in the treatment of cancer^1^ and genetic disorders,^2^ and the recent approval of several antisense oligonucleotides (ASOs) including Inclisiran and Mipomersen for chronic diseases has intensified interest in the field.^3^ ASOs exert their effects through several alternative mechanisms, such as splice modulating, exon-skipping, siRNA-mediated gene silencing, or RNase-H mediated mRNA degradation.^4^ ASOs do not readily pass-through cell membranes, and some cell types, such as those in muscle and brain are very difficult to address. Hence, the further development of ASOs with enhanced properties is essential.^5^ The nuclease resistance and cell uptake properties of ASOs can be improved through chemical modification of the sugar phosphate backbone.^6^ Natural oligonucleotides are rapidly digested by nucleases in cells, and modifications including 2’O-alkyl, and 2′-fluoro sugars and phosphorothioate (PS) backbones^6b^ are used to overcome these challenges.^4e,7^ However, while the PS backbone increases nuclease resistance,^8^ it also reduces RNA target affinity.^9^ To compensate for this, modified sugars such as locked nucleic acid (LNA) are employed to boost RNA affinity^8,10^ and confer resistance to nucleases. Reducing the net anionic charge of the oligonucleotide is another method that has been used to increase nuclease resistance and cell uptake.^11^ This can be achieved through the use of charge-neutral or positively charged internucleotide linkages.^12^

An uncharged DNA backbone containing the sulfamate group has been reported by Huie et al.^13^ This structure, unlike phosphorothioate, has the advantage of being achiral, and is essentially isostructural with the natural DNA phosphodiester backbone (Figure 1). However, although the 3′-O-sulfamate backbone contributes to nuclease resistance, it also reduces duplex stability. Fettes described 3′-N-sulfamate and sulfamide structures in DNA^14^ showing that the 3′-N-sulfamate slightly increases duplex stability. Both the above studies were carried out on oligonucleotides with unmodified phosphodiester backbones which are unsuitable for use in vivo. In this work, our aim was to increase RNA binding affinity by combining LNA sugars with sulfonyl backbone variants and to insert these into 2′-O-methyl-PS modified oligonucleotides. To achieve this, it was first necessary to establish methods to synthesize ASOs containing LNA- sulfamate and sulfamide linkages.

**Figure 1:**
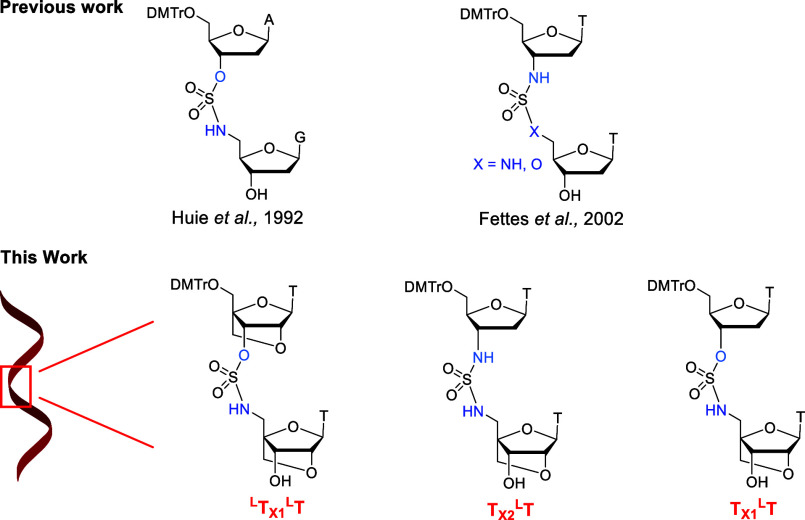
Previously synthesized sulfamate and sulfamide backbones compared to the current work.

Our key objective was to synthesis oligonucleotides containing the artificial backbones shown in Figure 1, with LNA located below, or above and below it. This would allow us to evaluate the positional influence of LNA on duplex stability. Our synthetic strategy is outlined in Scheme 1. Commercially available compound **1** was converted to protected LNA-T nucleoside **2** following a reported procedure (SI Scheme S1).^15^ Next, the benzyl group was removed by catalytic hydrogenation to give **3**, and the 3′–OH group was protected with *tert*-butyldimethylsilyl to give **4**.^16^ The mesyl group was then replaced by azide to give **5** which was reduced to the amine by catalytic hydrogenation,^12a^ yielding TBS-protected 5′-NH_2_-LNA-T nucleoside **6** (Scheme 1A). Intermediate **7**(14) for use in dinucleotide synthesis was prepared by reacting **6** with p-nitrophenylsulfurochloridate in the presence of p-nitrophenol and triethylamine (Scheme 1B). We then reacted activated p-nitrosulfamate nucleotide analogue **7** with nucleosides **8** (SI Scheme S2),^15,17^**9** (SI Scheme S3),^18^ and **10** as shown in Scheme 2.

**Scheme 1:**
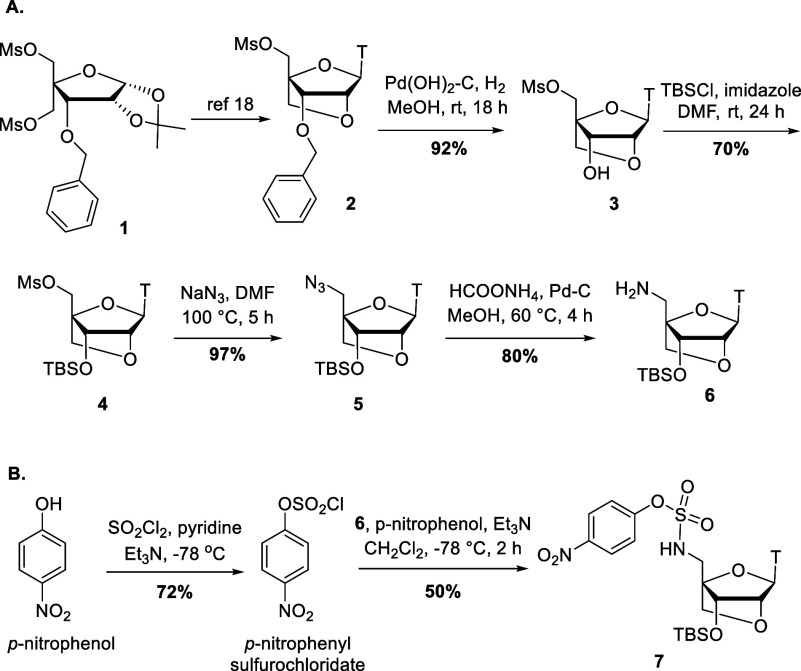
A. Synthesis of TBS Protected 5′-Amino-LNA-thymidine 6. B. Synthesis of Sulfamate Intermediate **7**

**Scheme 2:**
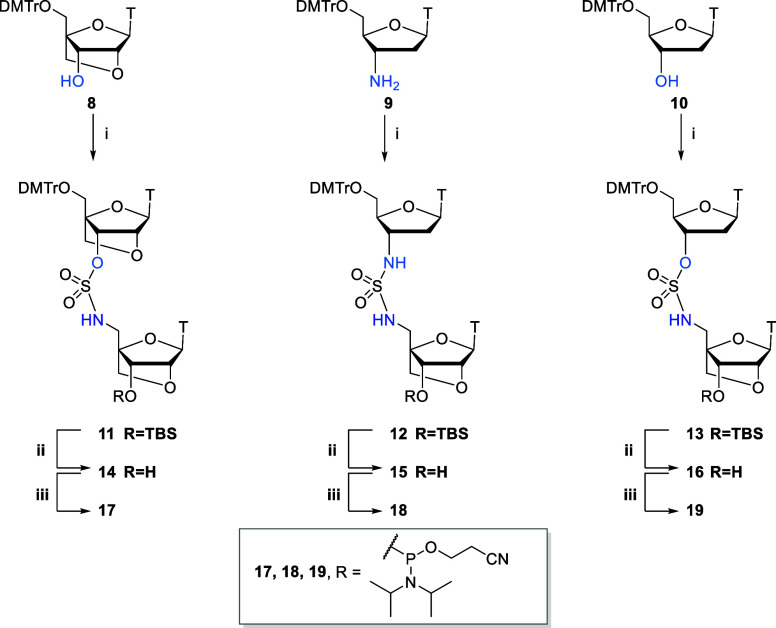
Synthesis of Sulfamate/Sulfamide Backbone Dinucleotide Phosphoramidites Reagents and conditions: (i) **7**, DMAP, THF, rt, 18 h, **11** 38%, **12** 80%, **13** 45%; (ii) TBAF, THF, rt, 5 h, **14** 90%, **15** 88%, **16** 88%; (iii) Chloro (diisopropylamino)-β-cyanoethoxyphosphine, Et_3_N, THF, rt, 3 h, **17** 65%, **18** 58%, **19** 72%.

Reaction of **7** with **8** gave the LNA-LNA O3′ → N5′ sulfamate dinucleotide **11**. Initially Et_3_N was used as the base for the sulfamate coupling step with limited success. Several reaction conditions were tried, but in all cases very low yields resulted (10–20%). We then switched from Et_3_N to DMAP and higher yields were obtained for all reactions involving **7**. After TBS deprotection of **11**, compound **14**(18c) was phosphitylated to give LNA-LNA O3′ → N5′ sulfamate dinucleotide **17** for use in oligonucleotide synthesis. Similarly, reaction of **7** with **9** gave DNA-LNA N3′ → N5′ sulfamide dinucleotide **12** in 80% yield. The TBS group was removed to give **15** which was converted to phosphoramidite **18**. Finally, reaction between compounds **7** and **10** gave DNA-LNA O3′ → N5′ sulfamate dinucleotide **13** in a yield of 45%. TBS deprotection gave **16** which was converted to dinucleotide phosphoramidite **19** (Scheme 2).

Modified dinucleotide phosphoramidites **17**, **18** and **19** were used to prepare oligonucleotides on an Applied Biosystems ABI-394 DNA synthesizer. The oligonucleotides (**ON1-ON5**) contained either one or two sulfa-type linkages and the other linkages were 2′-O-methyl phosphorothioates for compatibility with cell-based assays. The sequence is designed to target a splice site in model HeLa Luc cells to restore the aberrant luciferase reading frame and give a luminescent readout of exon skipping (Table 1).^12a,19^ Initially deprotection of all oligonucleotides was carried out at room temperature using a 1:1 mixture of ethylene diamine (EDA) and THF, commonly used for oligonucleotides such as alkyl phosphonates which are unstable to ammonia deprotection (Table S1). The observed mass of the DMT-ON oligonucleotide **ON1** containing the LNA-LNA O3′ → N5′ sulfamate was correct, but the presence of one and two acetyl groups was observed for **ON3** and **ON2** respectively. Both oligonucleotides contain the DNA-LNA N3′ → N5′ sulfamide, and it is apparent that the acetic anhydride/*N*-methylimidazole capping reagent used in oligonucleotide synthesis reacts with the backbone nitrogen atoms of the sulfamide linkage. The mass spectrum of **ON5** (DNA-LNA O3′ → N5′ sulfamate linkage) gave disappointing results, suggesting cleavage of the sulfamate backbone (Figure 2A). We were unable to obtain interpretable mass data for **ON4** due to the presence of two unstable DNA-LNA O3′ → N5′ sulfamate linkages which leads to a mixture of short strands. Changing the deprotection conditions to concentrated aqueous ammonia (55 °C, 5h) gave the desired masses for oligonucleotides **ON1**, **ON2**, and **ON3**. Importantly, the N atom of the N3′ → N5′ sulfamide backbone, which was acetylated during the solid-phase synthesis, was successfully deacetylated by ammonia treatment. Yet again, incorrect mass was observed for **ON5**, due to cleavage of the S–O bond, and no clear mass was observed for **ON4**. The mass spectrum of **ON5** suggested acetylation of the backbone NH and cleavage at the 3′-oxygen of the sulfonyl group (Figure 2A). Unfortunately, milder treatment of **ON4** and **ON5** with ammonia at room temperature failed to solve the problem. We reasoned that the electron withdrawing acetyl group negates the stabilizing effect of the backbone sulfamate nitrogen.

**Table 1:**
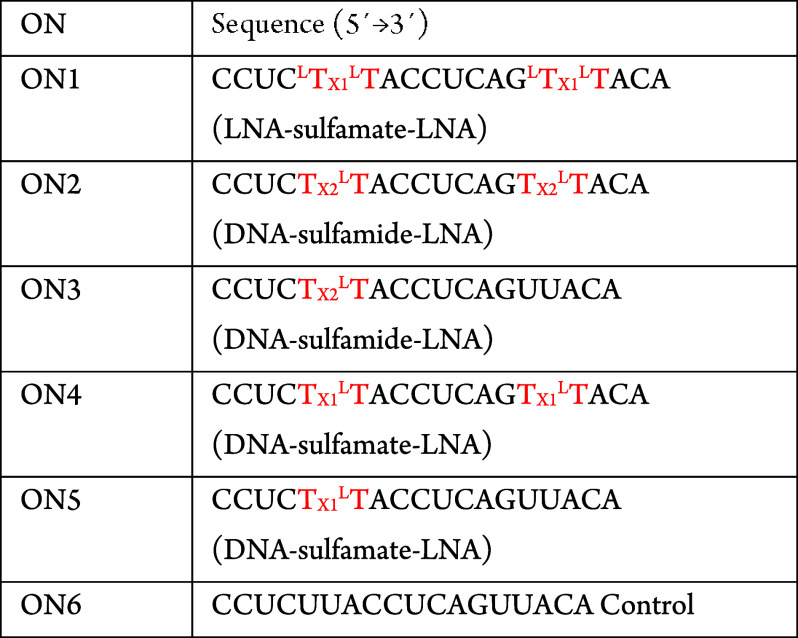
Oligonucleotides: All Oligonucleotides Are 2′-OMe Phosphorothioate Except for Any Sulfa-Type Linkages

**Figure 2:**
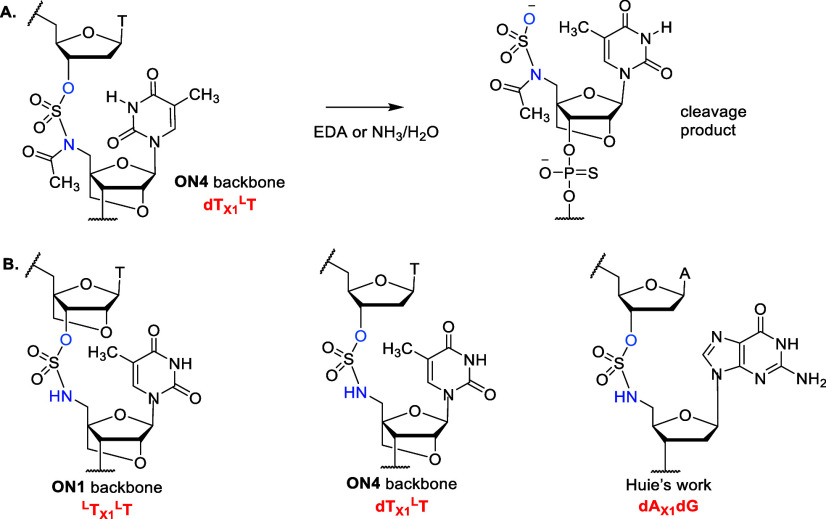
**A.** Cleavage of the acetylated sulfamate backbone during oligonucleotide deprotection. **B.** Sulfamate backbones that are stable during deprotection.

Pleasingly, by omitting the capping step during solid-phase synthesis, we were able to successfully obtain oligonucleotides **ON4** and **ON5** that contain the DNA-LNA O3′ → N5′ sulfamate linkage (Table S1). The purity of these oligonucleotides is slightly compromised by the lack of the capping step, which makes trityl-on HPLC purification less effective.

Interestingly, in the study conducted by Huie et al.,^13^ oligonucleotides containing the DNA–DNA O3′ → N5′ sulfamate linkage were successfully deprotected with 27% ammonia. The sulfamate dimer sequence used by Huie was 5′- dA_X1_dG-3′ whereas we used 5′- dT_X1_^L^T-3′, (Figure 2B). The bulkiness of the purine ring may possibly hinder nucleophilic attack at the sulfamate linkage or even prevent backbone acetylation. The reason that the LNA-LNA O3′ → N5′ sulfamate linkage is more stable to deprotection than DNA-LNA O3′ → N5′ sulfamate is probably steric in nature, but the electronegative ring oxygen of the LNA sugar might also hinder the approach of nucleophiles. As expected, the DNA-LNA N3′ → N5′ sulfamide linkage is very stable to oligonucleotide deprotection as was found for its deoxyribose analogue.^14^

The 5′-DMT groups were removed from all oligonucleotides by treatment with 80% aqueous acetic acid, and mass spectrometry was used to characterize them (Table S2).

Analysis of duplex stability by UV-melting (Table 2, SIFigure S29) showed that the LNA-LNA O3′ → N5′ sulfamate linkage (**ON1**) increases the Tm against complementary DNA, and RNA by more than 4 °C per modification. The DNA-LNA O3′ → N5′ sulfamate linkage was less effective, only slightly improving the stability of duplexes with its DNA and RNA complements (**ON4**, **ON5**). Comparison of melting temperatures of LNA-LNA O3′ → N5′ and DNA-LNA O3′ → N5′ sulfamate linkages shows that the 5′-LNA sugar has a strong positive influence on the artificial sulfamate backbone. Interestingly HPLC/MS showed that oligonucleotides containing DNA-LNA O3′ → N5′ sulfamate linkages fragment at the sulfonyl group during repeated heating cycles of UV melting, giving rise to complex melting curves. For this reason, the later cycles were omitted from the Tm calculations. In contrast, the LNA-sulfamate-LNA oligonucleotide (**ON1**) was stable during UV melting.

**Table 2:** UV Melting Results^a^

	DNA Target		RNA Target	
ON	Tm, °C	ΔTm	Tm, °C	ΔTm
ON1	57.6	+9.1	77.7 (71.1*)	+8.2
ON2	55.8	+7.3	74.1 (71.0**)	+4.6
ON3	52.5	+4.0	72.1	+2.6
ON2-Ac*	52.0	+3.5	69.0	–0.5
ON3-Ac	50.0	+1.5	70.2	+0.7
ON4	49.7	+1.2	68.9	–0.6
ON5	48.9	+0.4	69.3	–0.2
ON6	48.5		69.5 (61.3*, 65.5**)	

a Target DNA strand = 5′-TGTAACTGAGGTAAGAGG-3′. Target RNA strand 5′-UGUAACUGAGGUAAGAGG-3′. *ON3-Ac was obtained as a mono- and diacetylated mixture. The experiments were performed in 100 mM NaCl, 10 mM phosphate buffer, pH 7.0. Tm = Melting temperature, Δ*T*_m_ = (*T*_m_ of the modified duplex-Tm of the unmodified duplex). *Tm value measured in 25 mM NaCl, 10 mM phosphate buffer, pH 7.0; and **Tm values were measured in 50 mM NaCl, 10 mM phosphate buffer, pH 7.

The DNA-LNA N3′ → N5′ sulfamide linkages in **ON2** (2 sulfamides) and **ON3** (1 sulfamide) increase the Tm by +7.3 and 4.0 °C respectively against complementary DNA and by 4.6 and 2.6 °C against complementary RNA. Duplex stabilization is less for the acetylated sulfamide backbone (Ac) in **ON2_Ac** and **ON3_Ac.**

Fettes et al.^14^ found that a single DNA–DNA N3′ → O5′ sulfamate barely stabilized a DNA duplex (+0.1 °C), and two and three N3′ → O5′ sulfamate linkages had a negligible cumulative effect. The same linkage destabilized the duplex with RNA by 1.2 °C. They also found that DNA–DNA N3′ → N5′ sulfamide linkages destabilize the duplex against both DNA and RNA (−3.2 °C). Hence, our strategy of replacing the deoxyribose sugar in these artificial sulfa-type backbones along with LNA has a significant beneficial effect on duplex stability, and the LNA-sulfamate-LNA combination is particularly effective.

Alterations to the global duplex structures of the backbone-modified oligonucleotides against complementary DNA and RNA were determined by circular dichroism (CD) (SI Figure S30). The helical conformations are only slightly affected in comparison to the control **ON6**. Increasing the proportion of LNA in duplexes containing O3′→N5′ sulfamate (**ON1**) and N3′→N5′ sulfamide (**ON2**) produced a slight hypsochromic shift.

All oligonucleotides with sulfamate or sulfamide linkages remained stable to endonuclease S1 from *Aspergillus oryzae* after 2 days, whereas the unmodified oligonucleotide was degraded in less than 1 h (SI Figures S31–S35). This strongly suggests that sulfamate and sulfamide backbones will not be substrates for cellular nucleases.

In conclusion, oligonucleotides containing LNA-O3′→N5′ sulfamate-LNA and DNA-N3′→N5′ sulfamide-LNA linkages were synthesized using a standard solid-phase dinucleotide phosphoramidite strategy. The method could potentially be carried out on a large scale. These new backbone modifications, particularly LNA-O3′→N5′ sulfamate-LNA, hybridize to complementary RNA with high affinity and show strong resistance to enzymatic degradation. Poor cellular uptake remains a significant hurdle in oligonucleotide therapeutics, and PS and LNA modifications with neutral backbones such as sulfamate have the potential to improve clinical efficacy.^12a^ In this context we are planning to evaluate oligonucleotide analogues with different numbers of LNA-sulfamate and LNA-sulfamide backbones in various therapeutic assays.

## Data Availability

The data underlying this study are available in the published article and its Supporting Information.

## Supporting Information Available

Schemes S1–S3, Tables S1, S2, experimental procedures, analytical data of synthesized compounds, ^1^H and ^13^C NMR spectra of novel compounds, and UPLC-HRMS of oligonucleotides (PDF)

## References

[ref1] Xiong H.; Veedu R. N.; Diermeier S. D. Recent Advances in Oligonucleotide Therapeutics in Oncology. Int. J. Mol. Sci. 2021, 22 (7), 3295 10.3390/ijms22073295. 33804856 PMC8036554

[ref2] Koo T.; Wood M. J. Clinical trials using antisense oligonucleotides in duchenne muscular dystrophy. Hum. Gene Ther. 2013, 24 (5), 479–488. 10.1089/hum.2012.234. 23521559

[ref3] aRaguraman P.; Balachandran A. A.; Chen S. X.; Diermeier S. D.; Veedu R. N. Antisense Oligonucleotide-Mediated Splice Switching: Potential Therapeutic Approach for Cancer Mitigation. Cancers (Basel) 2021, 13 (21), 5555 10.3390/cancers13215555. 34771719 PMC8583451

[ref4] aIto K. R.; Obika S. Recent Advances in Medicinal Chemistry of Antisense Oligonucleotides. Comprehensive Medicinal Chemistry Iii, Vol 6: Biologics Medicine 2017, 216–232. 10.1016/B978-0-12-409547-2.12420-5.

[ref5] aRoberts T. C.; Langer R.; Wood M. J. A. Advances in oligonucleotide drug delivery. Nat. Rev. Drug Discovery 2020, 19 (10), 673–694. 10.1038/s41573-020-0075-7. 32782413 PMC7419031

[ref6] aIwamoto N.; Butler D. C. D.; Svrzikapa N.; Mohapatra S.; Zlatev I.; Sah D. W. Y.; Meena; Standley S. M.; Lu G.; Apponi L. H.; et al. Control of phosphorothioate stereochemistry substantially increases the efficacy of antisense oligonucleotides. Nat. Biotechnol. 2017, 35 (9), 845–851. 10.1038/nbt.3948. 28829437

[ref7] Rinaldi C.; Wood M. J. A. Antisense oligonucleotides: the next frontier for treatment of neurological disorders. Nat. Rev. Neurol 2018, 14 (1), 9–21. 10.1038/nrneurol.2017.148. 29192260

[ref8] Wan W. B.; Seth P. P. The Medicinal Chemistry of Therapeutic Oligonucleotides. J. Med. Chem. 2016, 59 (21), 9645–9667. 10.1021/acs.jmedchem.6b00551. 27434100

[ref9] Boczkowska M.; Guga P.; Stec W. J. Stereodefined Phosphorothioate Analogues of DNA: Relative Thermodynamic Stability of the Model PS-DNA/DNA and PS-DNA/RNA Complexes. Biochemistry 2002, 41 (41), 12483–12487. 10.1021/bi026225z. 12369839

[ref10] aPallan P. S.; Allerson C. R.; Berdeja A.; Seth P. P.; Swayze E. E.; Prakash T. P.; Egli M. Structure and nuclease resistance of 2 ’,4 ’-constrained 2 ’-O-methoxyethyl (cMOE) and 2 ’-O-ethyl (cEt) modified DNAs. Chem. Commun. 2012, 48 (66), 8195–8197. 10.1039/c2cc32286b. PMC3404228 22614180

[ref11] aSheehan D.; Lunstad B.; Yamada C. M.; Stell B. G.; Caruthers M. H.; Dellinger D. J. Biochemical properties of phosphonoacetate and thiophosphonoacetate oligodeoxyribonucleotides. Nucleic Acids Res. 2003, 31 (14), 4109–4118. 10.1093/nar/gkg439. 12853628 PMC165954

[ref12] aBaker Y. R.; Thorpe C.; Chen J.; Poller L. M.; Cox L.; Kumar P.; Lim W. F.; Lie L.; McClorey G.; Epple S.; et al. An LNA-amide modification that enhances the cell uptake and activity of phosphorothioate exon-skipping oligonucleotides. Nat. Commun. 2022, 13 (1), 4036 10.1038/s41467-022-31636-2. 35821218 PMC9276774

[ref13] Huie E. M.; Kirshenbaum M. R.; Trainor G. L. Oligonucleotides with a Nuclease-Resistant Sulfur-Based Linkage. J. Org. Chem. 1992, 57 (17), 4569–4570. 10.1021/jo00043a004.

[ref14] Fettes K. J.; Howard N.; Hickman D. T.; Adah S.; Player M. R.; Torrence P. F.; Micklefield J. Synthesis and nucleic-acid-binding properties of sulfamide- and 3′sulfamate-modified DNA. J. Chem. Soc.-Perkin Trans. 1 2002, (4), 485–495. 10.1039/b110603c.

[ref15] Koshkin A. A.; Fensholdt J.; Pfundheller H. M.; Lomholt C. A simplified and efficient route to 2’-O, 4’-C-methylene-linked bicyclic ribonucleosides (locked nucleic acid). J. Org. Chem. 2001, 66 (25), 8504–8512. 10.1021/jo010732p. 11735531

[ref16] Beni Y.; Dash C.; Parang K. Synthesis of beta-triphosphotriester pronucleotides. Tetrahedron Lett. 2015, 56 (17), 2247–2250. 10.1016/j.tetlet.2015.03.036. 26661734 PMC4675357

[ref17] Nielsen P.; Christensen N. K.; Dalskov J. K. α-LNA (locked nucleic acid with α-D-configuration): Synthesis and selective parallel recognition of RNA. Chem.—Eur. J. 2002, 8 (3), 712–722. 10.1002/1521-3765(20020201)8:3<712::AID-CHEM712>3.0.CO;2-0. 11855719

[ref18] aPalframan M. J.; Alharthy R. D.; Powalowska P. K.; Hayes C. J. Synthesis of triazole-linked morpholino oligonucleotides via Cu(I) catalysed cycloaddition. Org. Biomol Chem. 2016, 14 (11), 3112–3119. 10.1039/C6OB00007J. 26905296 PMC5047124

[ref19] Kang S. H.; Cho M. J.; Kole R. Up-regulation of luciferase gene expression with antisense oligonucleotides: Implications and applications in functional assay developments. Biochemistry 1998, 37 (18), 6235–6239. 10.1021/bi980300h. 9572837

