# Supplementary material for: Synthesis and Properties of Oligonucleotides Containing LNA-Sulfamate and Sulfamide Backbone Linkages
Source: Org Lett. 2024 May 8;26(19):4137–41. doi: 10.1021/acs.orglett.4c01232 (PMC11110047; doi:10.1021/acs.orglett.4c01232)
Supplement: Supplementary file 1 — ol4c01232_si_001.pdf [file ol4c01232_si_001.pdf]

Supporting Information

Synthesis and Properties of Oligonucleotides Containing LNA-Sulfamate and Sulfamide Backbone Linkages

Belma Zengin Kurt[1,2#], Debashis Dhara[1#], Afaf H. El-Sagheer[1,3*], Tom Brown[1*].

1. Department of Chemistry, University of Oxford, Chemistry Research Laboratory, Oxford, OX1 3TA, UK.

2. Department of Pharmaceutical Chemistry, Bezmialem Vakif University, Faculty of Pharmacy, 34093, Istanbul, Türkiye

3. School of Chemistry, University of Southampton, Highfield, Southampton, SO17 1BJ, UK.

*Corresponding authors. #Joint first authors.

e-mail: tom.brown@chem.ox.ac.uk, ahes@soton.ac.uk

Table of Content

1. General Information S3
2. Supplementary schemes S3
3. Experimental procedures S5
4. Synthesis, purification and analysis of oligonucleotides S19
5. UPLC and Mass Spectra of the Oligonucleotides S21
6. UV melting experiments S36
7. Circular Dichroism S37
8. Nuclease stability S38
9. References S41
10. NMR spectra of compounds S42

1. General Information

Reactions were performed in oven-dried glassware under an inert atmosphere of argon using anhydrous solvents which were collected from an mBraun SPS-800 bench-top solvent purification system. Solvents for phosphitylation reactions were degassed by bubbling argon gas through before use. All other chemicals were used as obtained from commercial sources without further purification. Thin layer chromatography (TLC) was performed using Merck pre-coated 0.23 mm thick plates of Kieselgel 60 F254 and visualized using UV (λ = 254 nm) or by staining with p-anisaldehyde, or ninhydrin (depending on functionality). ^{1}H, ^{13}C, and ^{31}P NMR spectra were recorded on a Bruker AVH 400, AVG 400, or Bruker NEO 600 spectrometer operating at 400, 500, or 600 MHz respectively. Mass Spectroscopy: High-resolution mass spectra (HRMS) were recorded by the Chemistry Department Mass Spec. Service, University of Oxford on a Thermo Scientific Exactive Mass Spectrometer (Waters Equity autosampler and pump) - electrospray ionization (ESI) and an Agilent 7200 Accurate Mass QTOF GCMS (SIM Direct Insertion Probe) for electron ionization (EI) and chemical ionization (CI).

2. Suplementary Schemes

Synthesis of Key building blocks

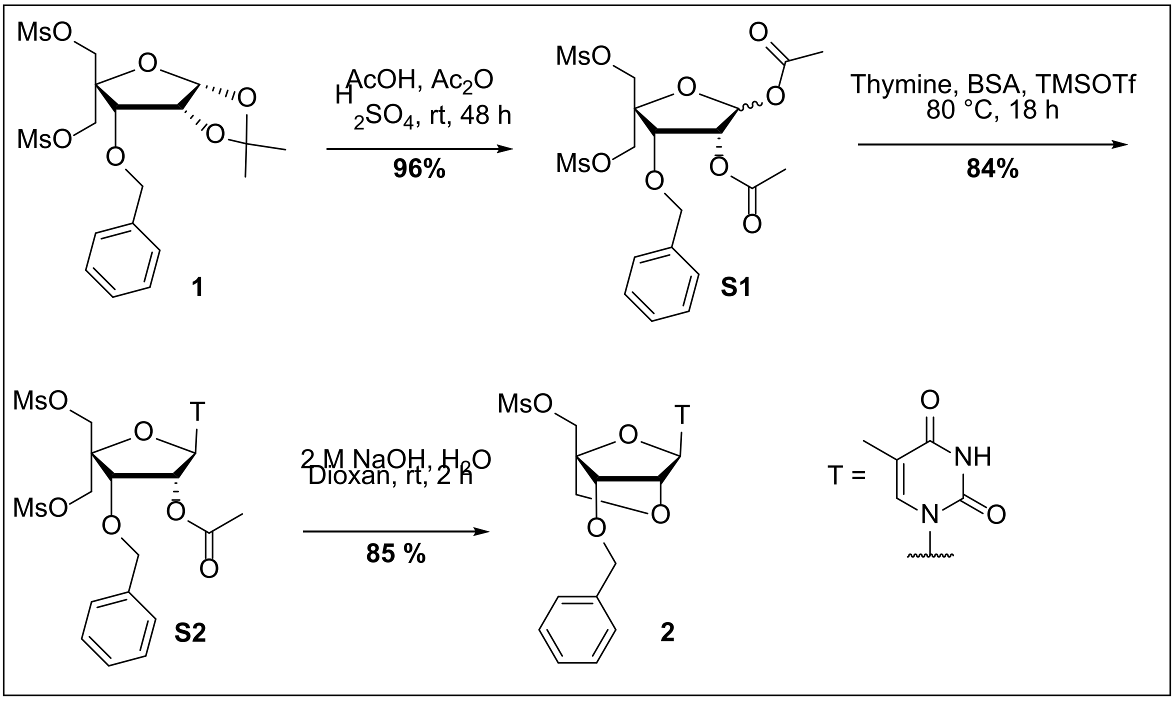

Suplementary Scheme S1. Synthesis of DMT protected LNA-thymidine (**2**)[1]

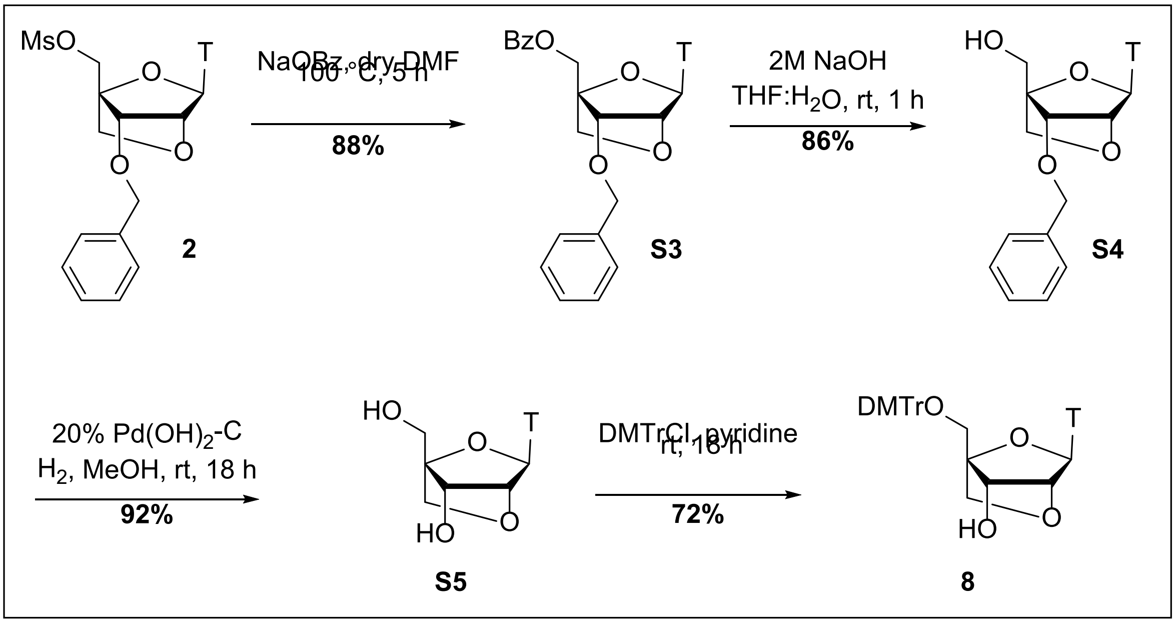

Suplementary Scheme S2. Synthesis of DMT protected LNA-thymidine (**8**). The compound **8** was prepared according to the literature procedure [1]. The data obtained are consistent with the literature.

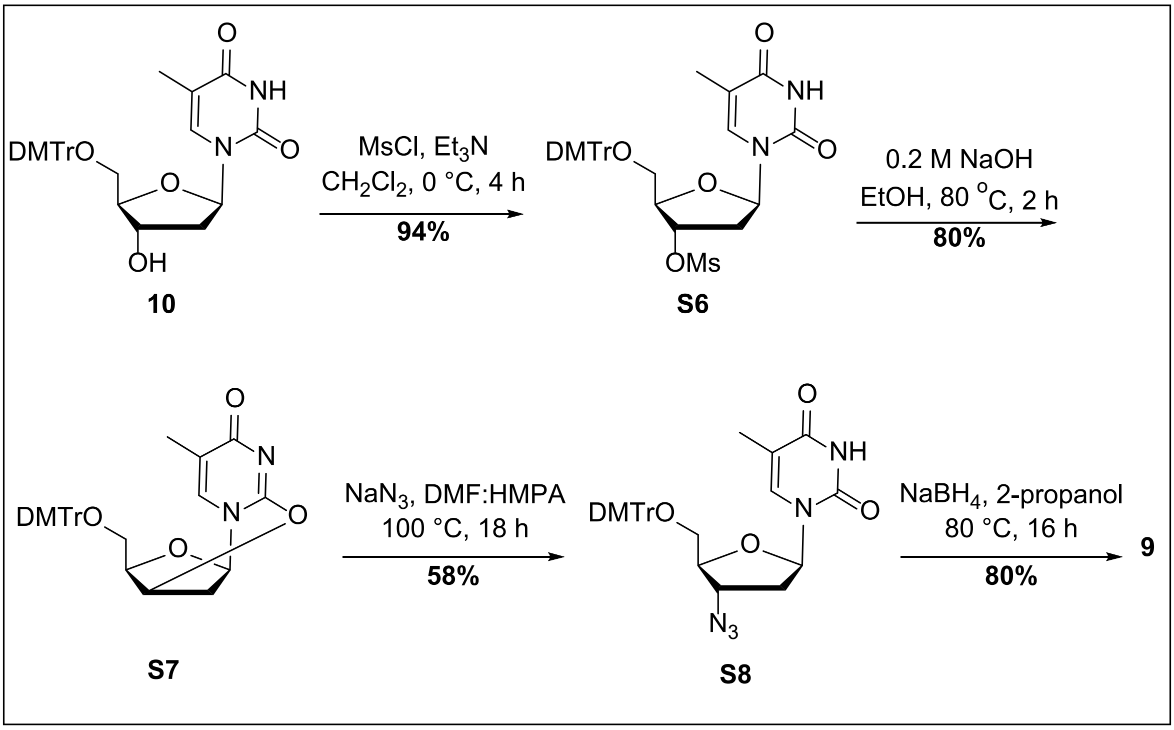

Suplamentary Scheme S3. Synthesis of DMT protected 3'-amino-3’-deoxythymidine (**9**) **4** was prepared according to the literature procedure [3,4,5]. The data obtained are consistent with the literature.

3. Experimental Procedure

Synthesis of Key Compounds

*1,2-Di-O-acetyl-3-O-benzyl-4-C-methanesulfonoxymethyl-5-O-methanesulfonyl-D-erythro-pentofuranose **(S1)***

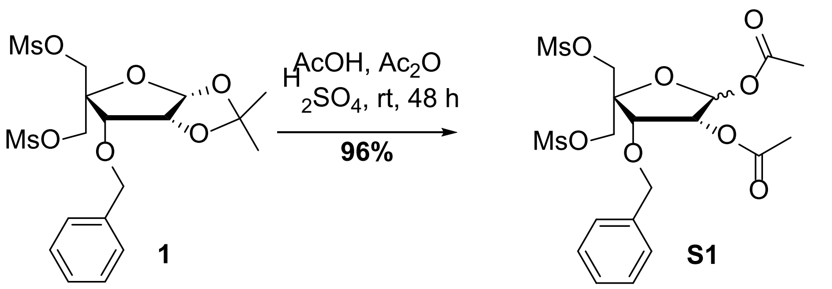

Chemical Formula: $C_{19}H_{26}O_{12}S_2$
Exact Mass: 510.0866

Ac_2O (7.5 mL, 80 mmol) and concentrated H_2SO_4 (7.5 µL) were added to a solution of compound **1** (5 g, 11 mmol) in AcOH (75 mL), and the mixture was stirred overnight at room temperature. More concentrated H_2SO_4 (1.5 µL) was added, and the reaction was continued for 24 h. H_2O (60 mL) was added, and the mixture was stirred for 3 h and washed twice with CH_2Cl_2 (70 and 30 mL). The combined organic layers were washed with saturated $NaHCO_3$ (4 x 60 mL), dried (Na_2SO_4), and concentrated under reduced pressure to give compound **S1** (5.3 g, 96% yield) as a colorless syrup. The analytical data were matched with the previously reported data[2].

*1-(2-O-Acetyl-3-O-benzyl-4-C-methanesulfonoxymethyl-5-O-methanesulfonyl-α-D-erythro-pentofuranosyl)thymine **(S2)***

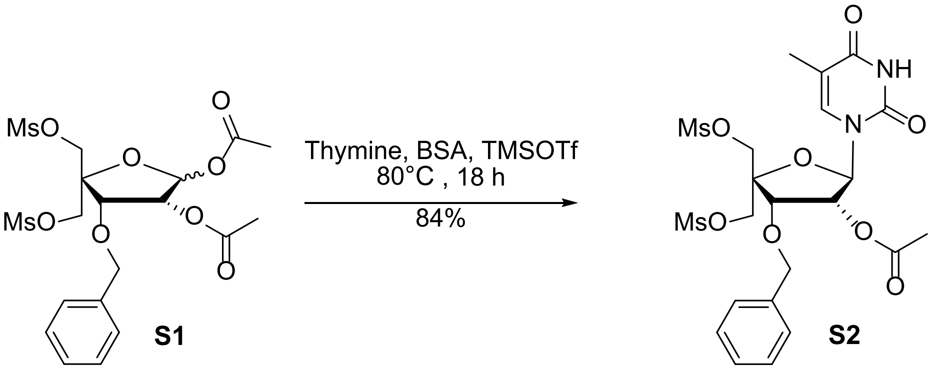

Chemical Formula: $C_{22}H_{28}N_2O_{12}S_2$
Exact Mass: 576,11

1g of **S1** (1.9 mmol) was added to a 50 mL round-bottom flask and dissolved in 10 mL of dry acetonitrile. Thymine (309 mg, 2.45 mmol)1.340 mL and N,O-bis(trimethylsilyl)acetamide (5.48 mmol) were added. The mixture was refluxed in a heating block for 45 min under N_2 gas, cooled to room temperature then trimethylsilyl trifluoromethanesulfonate (0.495 mL, 2.73 mmol) was added. The reaction was refluxed overnight, cooled to room temperature, and poured onto saturated aqueous $NaHCO_3$. This was extracted three times with CH_2Cl_2 and the combined the organic phases were washed with saturated aqueous $NaHCO_3$ and then brine, dried (Na_2SO_4) concentrated. The crude was purified by flash column chromatography (1-3%

MeOH/ CH_2Cl_2) to give **S2** (0.94 g, 84% yield). The analytical data were matched with the previously reported data[2].

((1R,3R,4R,7S)-7-(benzyloxy)-3-(5-methyl-2,4-dioxo-3,4-dihydro pyrimidin-1(2H)-yl)-2,5-dioxabicyclo[2.2.1]heptan-1-yl)methyl methanesulfonate ***(2)***

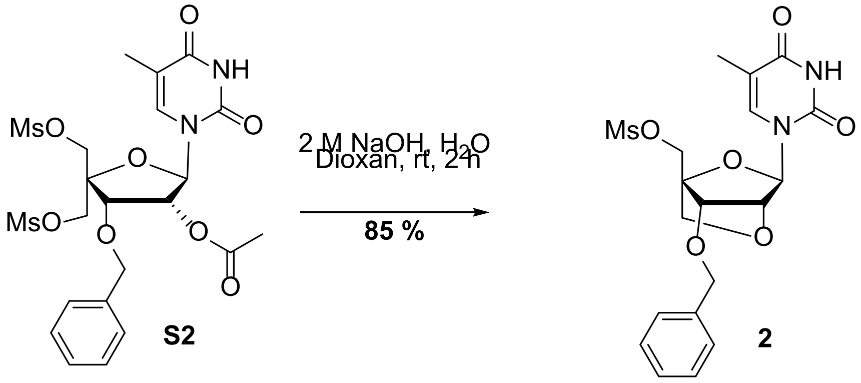

Chemical Formula: $C_{19}H_{22}N_2O_8S$
Exact Mass: 438,11

Compound **S2** (840 mg, 1.45 mmol) was dissolved in dioxan (10 mL) and water (10 mL), followed by the addition of NaOH (2M, 2 mL) and stirred at rt. $NaHCO_3$ is added to the mixture which was washed with CH_2Cl_2. The organic layers were dried with Na_2SO_4, filtered, and concentrated to dryness to yield the title compound **2** (550 mg, 85% yield). The analytical data were matched with the previously reported data[2].

((1R,3S,4R,7S)-7-hydroxy-3-(5-methyl-2,4-dioxo-3,4-dihydropyrimidin-1(2H)-yl)-2,5-dioxabicyclo [2.2.1]heptan-1-yl)methyl methanesulfonate ***(3)***

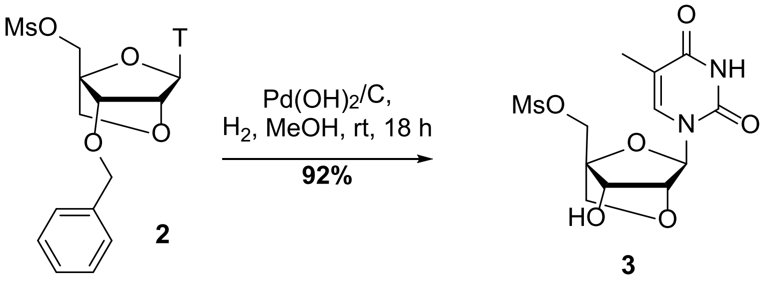

Chemical Formula: $C_{12}H_{16}N_2O_8S$
Molecular Weight: 348,33

Compound **2** (2 g, 4.55 mmol) was dissolved in MeOH (200 mL), followed by the addition of $Pd(OH)_2/C$ (320 mg, 2.28 mmol) and stirred in an inert atmosphere (N_2). The N_2 was replaced with H_2 and the reaction was stirred rt at 24 h. The $Pd(OH)_2/C$ catalyst was removed by filtration through celite, washed with MeOH (200 mL), and the combined filtrates were concentrated to dryness to yield the title compound **3** (1.46 g, 4.18 mmol, 92% yield) as a white solid.

^1H NMR (400 MHz, DMSO-*d6*) δ 11.38 (s, 1H), 7.51 (d, *J* = 1.5 Hz, 1H), 5.49 (s, 1H), 4.79 (d, *J* = 11.9 Hz, 1H), 4.60 (d, *J* = 12.0 Hz, 1H), 4.21 (s, 1H), 4.09 (s, 1H), 4.01 – 3.92 (m, 2H), 3.78 (d, *J* = 7.9 Hz, 1H), 3.28 (s, 3H), 1.80 (d, *J* = 1.1 Hz, 3H). ^{13}C NMR (101 MHz, DMSO-*d6*) δ 164.2, 150.3, 134.8, 109.2, 87.0, 86.0, 79.4, 71.1, 69.9, 66.3, 37.3, 12.6. HRMS (m/z) $[M+H]^+$ calcd. for $C_{12}H_{17}N_2O_8S^+$, 349.0700, found 349.0690.

((1R,3S,4R,7S)-7-((tert-butyldimethylsilyl)oxy)-3-(5-methyl-2,4-dioxo-3,4-dihydro pyrimidin-1(2H)-yl)-2,5-dioxabicyclo[2.2.1]heptan-1-yl)methyl methanesulfonate ***(4)***

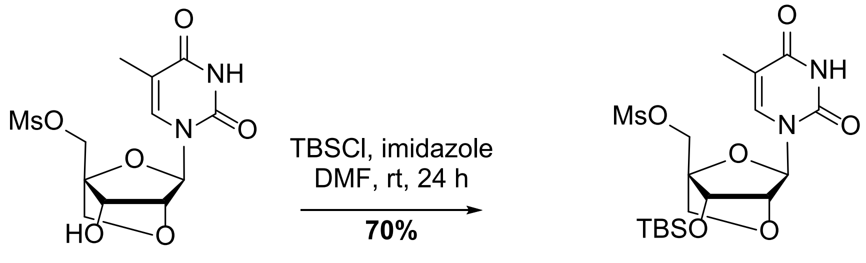

3 **4**

Chemical Formula: $C_{18}H_{30}N_2O_8SSi$
Exact Mass: 462,15

To a solution of **3** (1 g, 2.87 mmol) in DMF (10 mL) cooled to 0 °C was added imidazole (0.489 g, 7.18 mmol) followed by dropwise addition of TBSCl (0.780 g, 5.2 mmol) over a period of 10 min. The cooling bath was removed, and the reaction mixture was stirred at room temperature for 24 h. The solvent was evaporated; the residue was diluted with ethyl acetate (20 mL), washed with water, then saturated sodium bicarbonate, and dried over Na_2SO_4. The solvent was removed, and the resulting solid was purified by flash column chromatography, 5% MeOH in EtOAc to give the title compound **4** (0.9 g, 2.00 mmol, 70% yield).

^{1}H NMR (400 MHz, $CDCl_3$) δ 8.40 (s, 1H), 7.38 (q, J = 1.3 Hz, 1H), 5.51 (s, 1H), 4.48 (d, J = 11.7 Hz, 1H), 4.37 (d, J = 11.8 Hz, 1H), 4.28 (s, 1H), 3.97 (s, 1H), 3.92 (d, J = 7.8 Hz, 1H), 3.74 (d, J = 7.7 Hz, 1H), 3.00 (s, 3H), 1.86 (d, J = 1.3 Hz, 3H), 0.79 (s, 9H), 0.05 (s, 6H). ^{13}C NMR (101 MHz, $CDCl_3$) δ 163.4, 149.5, 134.3, 110.9, 87.6, 86.2, 79.1, 71.2, 70.9, 64.0, 37.9, 25.5, 17.9, 12.5, -4.7, -5.0. HRMS (m/z) $[M+Na]^+$ calcd. for $C_{18}H_{30}N_2O_8SSiNa^+$, 485.1384, found 485.1385.

1-((1S,3S,4R,7S)-1-(azidomethyl)-7-((tert-butyldimethylsilyl)oxy)-2,5-dioxabicyclo[2.2.1] heptan-3-yl)-5-methylpyrimidine-2,4(1H,3H)-dione ***(5)***

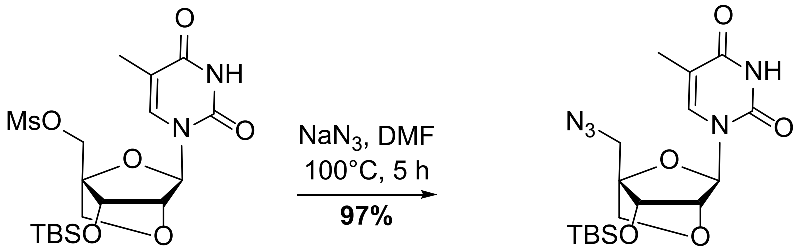

4 **5**

Chemical Formula: $C_{17}H_{27}N_5O_5Si$
Exact Mass: 409.18

NaN_3 (0.297 g, 4.58 mmol, 2 eq) and **4** (1.15 g, 2.29 mmol) were dissolved in DMF (40 mL) and the reaction was stirred at 100 °C for 5 h in a heating block. The solvent was removed under vacuum and the resulting residue was partitioned between EtOAc (40 mL) and water (40 mL). The organic layer was washed with water (2 x 40 mL), dried over Na_2SO_4, and evaporated to dryness. The crude was purified by flash column chromatography (50/50 PE:EtOAc) to give the compund **5** (0.99 g, 4.44 mmol, 97% yield) as a white solid.

^{1}H NMR (400 MHz, $CDCl_3$) δ 8.39 (s, 1H), 7.36 (q, *J* = 1.3 Hz, 1H), 5.49 (s, 1H), 4.26 (s, 1H), 3.92 (s, 1H), 3.85 (d, *J* = 7.7 Hz, 1H), 3.69 (d, *J* = 7.7 Hz, 1H), 3.63 (d, *J* = 13.6 Hz, 1H), 3.49 (d, *J* = 13.5 Hz, 1H), 1.87 (d, *J* = 1.2 Hz, 3H), 0.78 (s, 9H), 0.006 (s, 6H). ^{13}C NMR (101 MHz, $CDCl_3$) δ 163.4, 149.5, 134.3, 110.6, 87.6, 87.3, 79.0, 71.9, 71.1, 47.2, 25.5, 17.9, 12.8, -4.7, -5.0. HRMS (m/z) $[M+Na]^+$ calcd. for $C_{17}H_{27}N_5O_5SiNa^+$, 432.1679, found 432.1676.

1-((1S,3S,4R,7S)-1-(aminomethyl)-7-((tert-butyldimethylsilyl)oxy)-2,5-dioxabicyclo [2.2.1]heptan-3-yl)-5-methylpyrimidine-2,4(1H,3H)-dione ***(6)***

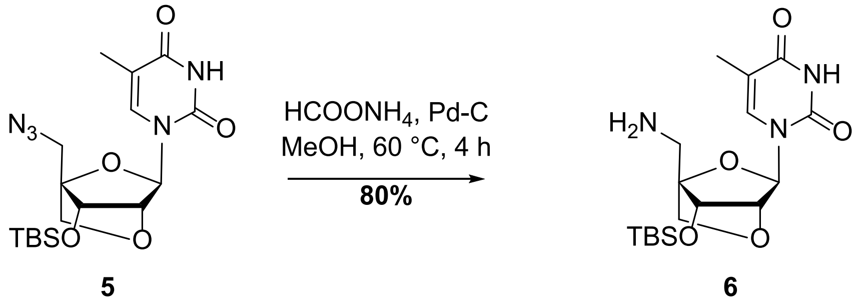

Chemical Formula: $C_{17}H_{29}N_3O_5Si$
Exact Mass: 383.1876

Compound **5** (0.5 g, 1.22 mmol) and ammonium formate (0.9 g, 14.7 mmol, 12 eq) were dissolved in MeOH (50 mL) and 20 wt% palladium hydroxide on carbon (34 mg, 0.2 mmol, 20 mol%) was added. The flask was flushed with argon and the reaction was stirred at 60 °C for 4 h in a heating block. The reaction was filtered through celite to remove the catalyst and the solvent was removed under vacuum. The resulting solid was purified by flash column chromatography (0-30% MeOH in EtOAc) to give **6** (375 mg, 0.9 mmol) as a white solid in 80% yield.

^{1}H NMR (400 MHz, DMSO-*d6*) δ 7.59 (d, *J* = 1.4 Hz, 1H), 5.37 (s, 1H), 4.08 (s, 1H), 4.00 (s, 1H), 3.73 (d, *J* = 7.8 Hz, 1H), 3.60 (d, *J* = 7.8 Hz, 1H), 2.85 (d, *J* = 2.1 Hz, 2H), 1.73 (d, *J* = 1.2 Hz, 3H), 0.78 (s, 9H), 0.018 (s, 3H), 0.011 (s, 3H). ^{13}C NMR (101 MHz, DMSO-*d6*) δ 164.3, 150.4, 135.4, 108.9, 89.5, 86.9, 79.2, 72.4, 70.9, 40.6, 40.4, 40.2, 37.8, 25.9, 18.1, 12.8, -4.4, -4.5. HRMS (m/z) $[M+Na]^+$ calcd. for $C_{17}H_{29}N_3O_5SiNa^+$, 406.1769, found 406.1765.

4-nitrophenyl (((1S,3R,4R,7S)-7-((tert-butyldimethylsilyl)oxy)-3-(5-methyl-2,4-dioxo-3,4-dihydropyrimidin-1(2H)-yl)-2,5-dioxabicyclo[2.2.1]heptan-1-yl)methyl)sulfamate ***(7)***

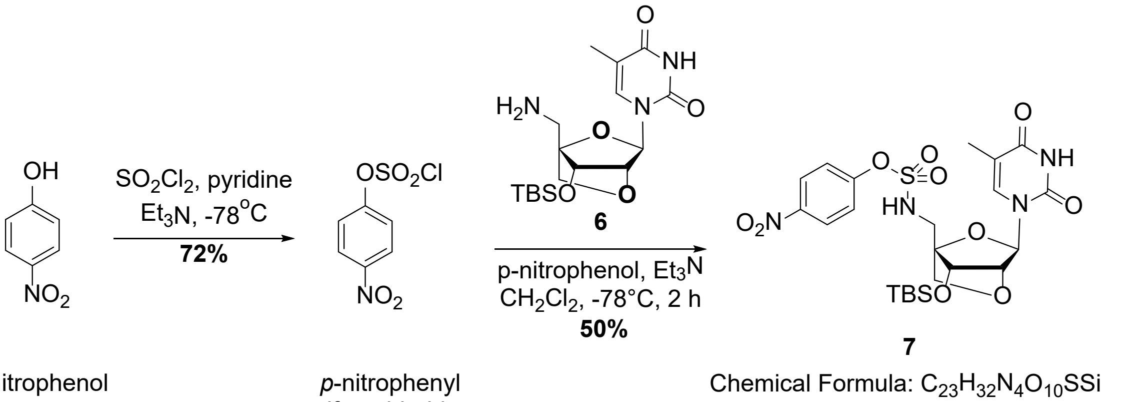

p-nitrophenol | *p*-nitrophenyl sulfurochloridate | Chemical Formula: $C_{23}H_{32}N_4O_{10}SSi$ Exact Mass: 584.1608

4-nitrophenyl sulfurochloridate; A solution of 4-nitrophenol (1.39 g, 10 mmol) and pyridine (0.81 mL, 10 mmol) in Et_2O (10 mL) was added dropwise to a solution of SO_2Cl_2 (0.80 mL, 10 mmol) in Et_2O (10 mL) at -78 °C under argon. The mixture was allowed to warm to room temperature,

stirred for 4 h, and then filtered through Celite. The residue was purified by flash chromatography (CH_2Cl_2–petroleum ether 1:1) (1.70 g, 72% yield).
^{1}H NMR (400 MHz, $CDCl_3$) δ 8.38 – 8.29 (m, 2H), 7.58 – 7.50 (m, 2H). ^{13}C NMR (101 MHz, $CDCl_3$) δ 153.4, 126.0, 122.9.

Title compound; A solution of **6** (1 g, 2.60 mmol), 4-nitrophenol (3.63 g, 26.1 mmol) and Et_3N (4.36 mL, 31.3 mmol) in dry CH_2Cl_2 (2.4 mL) was added dropwise over a period of 5 min to a solution of 4-nitrophenyl sulfurochloridate (1.24 g, 5.21 mmol) in dry CH_2Cl_2 (0.6 mL) at -78 °C under argon. After 1 h the reaction mixture was allowed to warm to room temperature, diluted with CH_2Cl_2 (15 mL), and washed with 1 M aq. NaH_2PO_4 (2 × 50 mL). The aqueous layers were then re-extracted with CH_2Cl_2 (3 × 50 mL) and the combined organic extracts were dried over Na_2SO_4 and evaporated under reduced pressure. Purification by flash chromatography, eluting with petroleum ether: EtOAc (0→50%) gave the desired product **7** (0.75 g, 50% yield) as a white solid.

^{1}H NMR (400 MHz, $CDCl_3$) δ 8.34 (s, 1H), 8.17 (d, *J* = 6.9 Hz, 2H), 7.33 (d, *J* = 7.0 Hz, 2H), 7.12 (d, *J* = 1.4 Hz, 1H), 5.72 (t, *J* = 6.2 Hz, 1H), 5.46 (s, 1H), 4.29 (s, 1H), 4.00 (d, *J* = 2.2 Hz, 1H), 3.93 (d, *J* = 7.9 Hz, 1H), 3.71 (d, *J* = 8.0 Hz, 1H), 3.59 (d, *J* = 6.8 Hz, 1H), 3.50 (dd, *J* = 14.3, 5.8 Hz, 1H), 1.78 (d, *J* = 1.2 Hz, 3H), 0.77 (s, 9H), -0.01 (s, 6H). ^{13}C NMR (101 MHz, $CDCl_3$) δ 163.2, 154.2, 149.4, 134.6, 125.7, 122.3, 88.3, 86.3, 79.3, 77.2, 72.0, 40.9, 25.5, 17.8, 12.6, -4.6. HRMS (m/z) $[M+H]^+$ calcd. for $C_{23}H_{33}N_4O_{10}SSi^+$, 585.1681, found 585.1701.

Synthesis of dinucleotides

*(1R,3R,4R,7S)-1-((bis(4-methoxyphenyl)(phenyl)methoxy)methyl)-3-(5-methyl-2,4-dioxo-3,4-dihydropyrimidin-1(2H)-yl)-2,5-dioxabicyclo[2.2.1]heptan-7-yl (((1S,3R,4R,7S)-7-((tert-butyldimethylsilyl)oxy)-3-(5-methyl-2,4-dioxo-3,4-dihydropyrimidin-1(2H)-yl)-2,5-dioxabicyclo[2.2.1]heptan-1-yl)methyl)sulfamate **(11)***

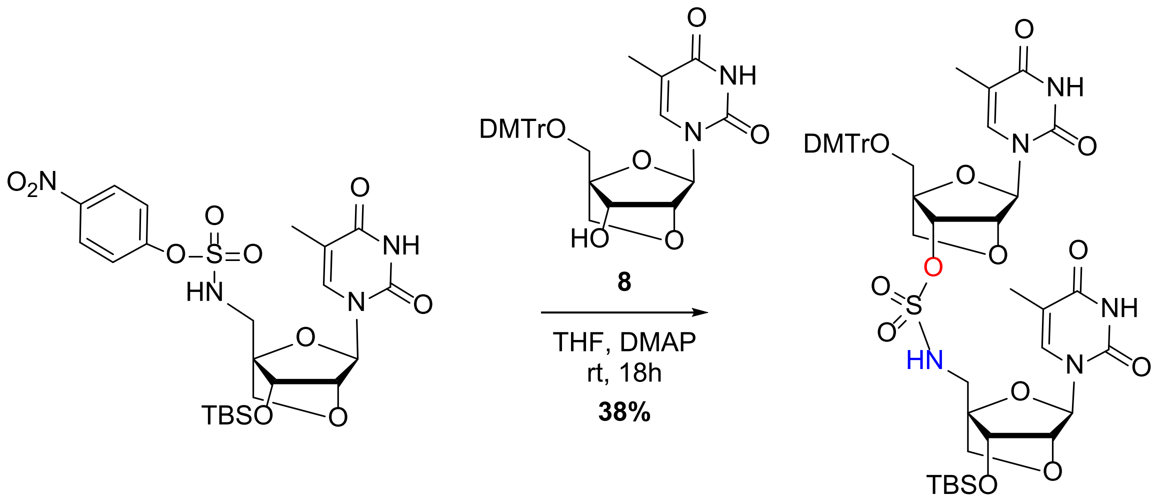

7

11

Chemical Formula: $C_{49}H_{59}N_5O_{15}SSi$
Exact Mass: 1017.3498

Sulfamate **7** (300 mg, 0.5 mmol) and nucleoside **8** (353 mg, 0.6 mmol) were dissolved in dry THF (3 mL) and dry molecular sieve (600 mg) was added. The reaction was stirred at room temperature for 1 hour in a sealed vial under argon. DMAP was then added and after 18 h the reaction mixture was filtered, diluted with EtOAc (50 mL), and washed with $NaHCO_3$ (1 × 20 mL), then saturated NaCl (1 × 20 mL). The organic layer was dried over Na_2SO_4 and the solvent was evaporated under reduced pressure. Crude compound **11** was used without purification (200 mg, 38% yield).

HRMS (m/z) $[M+Na]^+$ calcd. for $C_{49}H_{59}N_5NaO_{15}SSi^+$, 1040.3395, found 1040.3423.

*(2R,3S,5R)-2-((bis(4-methoxyphenyl)(phenyl)methoxy)methyl)-5-(5-methyl-2,4-dioxo-3,4-dihydropyrimidin-1(2H)-yl)tetrahydrofuran-3-yl (((1S,3R,4R,7S)-7-((tert-butyldimethylsilyl)oxy)-3-(5-methyl-2,4-dioxo-3,4-dihydropyrimidin-1(2H)-yl)-2,5-dioxabicyclo[2.2.1]heptan-1-yl)methyl)sulfamide **(12)***

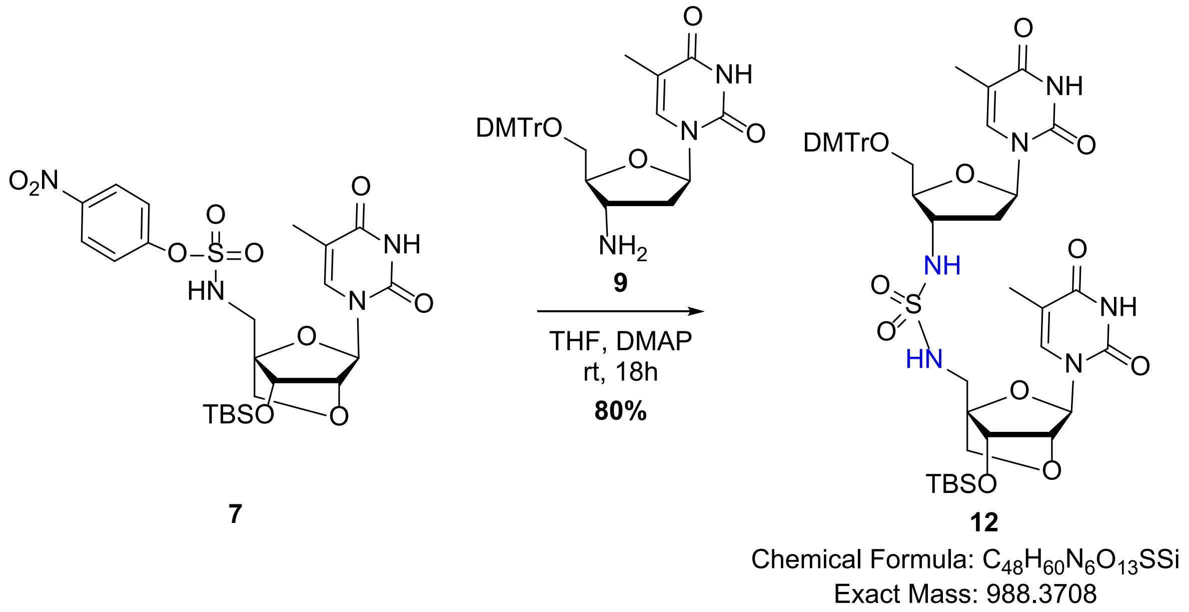

A mixture of sulfamate **7** (300 mg, 0.5 mmol), nucleoside **9** (335 mg, 0.6 mmol) and dry molecular sieve (600 mg) in dry THF (3 mL) was stirred at room temperature for 1 h in a sealed vial under argon. DMAP was added and the reaction was left to stir. After 18 h the reaction mixture was filtered, diluted with EtOAc (50 mL), washed with $NaHCO_3$ (1 × 20 mL), then saturated NaCl (1 × 20 mL). The organic layer was dried over Na_2SO_4 and the solvent was evaporated under reduced pressure. Purification by flash chromatography, eluting with 10 % MeOH in EtOAc gave the pure title compound **12** (406 mg, 80% yield) as a white solid.

^{1}H NMR (400 MHz, Acetone-*d6*) δ 9.88 (d, *J* = 7.1 Hz, 2H), 7.51 (dq, *J* = 3.7, 1.2 Hz, 2H), 7.43 – 7.39 (m, 2H), 7.30 – 7.26 (m, 4H), 7.25 – 7.19 (m, 2H), 7.18 – 7.12 (m, 1H), 6.85 – 6.74 (m, 4H), 6.58 (d, *J* = 8.3 Hz, 1H), 6.38 (t, *J* = 6.7 Hz, 1H), 6.18 (t, *J* = 6.5 Hz, 1H), 5.41 (s, 1H), 4.28 – 4.21 (m, 2H), 4.17 (s, 1H), 4.08 (ddd, *J* = 6.0, 3.7, 2.6 Hz, 1H), 3.89 (d, *J* = 7.8 Hz, 1H), 3.68 (s, 7H), 3.55 – 3.47 (m, 1H), 3.43 – 3.29 (m, 3H), 2.58 – 2.38 (m, 2H), 1.68 (d, *J* = 1.2 Hz, 3H), 1.34 (d, *J* = 1.2 Hz, 3H), 0.78 (s, 9H), 0.01 (s, 6H). ^{13}C NMR (101 MHz, Acetone-*d6*) δ 158.8, 135.7, 135.4, 134.7, 130.1, 128.1, 127.8, 126.8, 113.1, 110.0, 109.1, 87.5, 87.1, 84.2, 83.8, 79.3, 72.1, 71.6, 63.3, 54.6, 53.5, 39.7, 38.5, 28.73, 25.1, 17.6, 11.7, 11.2, -5.5, -5.6. HRMS (m/z) $[M+H]^+$ calcd. for $C_{48}H_{60}N_6NaO_{13}SSi^+$, 989.3708, found 989.3781.

*(2R,3S,5R)-2-((bis(4-methoxyphenyl)(phenyl)methoxy)methyl)-5-(5-methyl-2,4-dioxo-3,4-dihydropyrimidin-1(2H)-yl)tetrahydrofuran-3-yl(((1S,3R,4R,7S)-7-((tert-butyldimethylsilyl)oxy)-3-(5-methyl-2,4-dioxo-3,4-dihydropyrimidin-1(2H)-yl)-2,5-dioxabicyclo[2.2.1]heptan-1-yl)methyl)sulfamate **(13)***

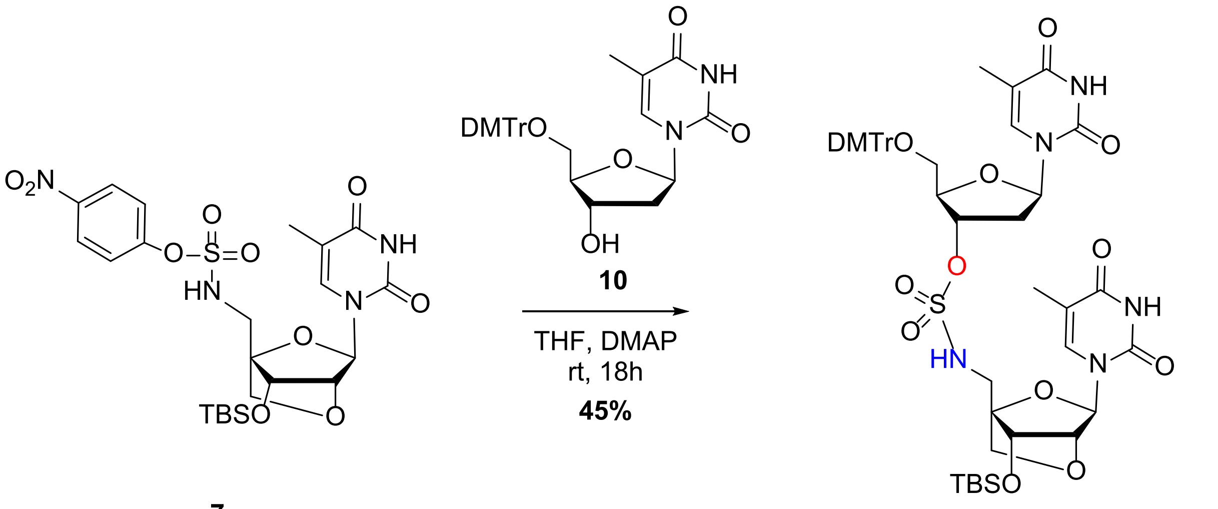

7

13

Chemical Formula: $C_{48}H_{59}N_5O_{14}SSi$
Exact Mass: 989.3548

To a mixture of sulfamate **7** (300 mg, 0.5 mmol) and nucleoside **10** (335 mg, 0.6 mmol) in dry THF (3 mL) was added dry molecular sieve (600 mg). The mixture was stirred at room temperature for 1 h in a sealed vial under argon. DMAP (313 mg, 2.57 mmol) was added. The reaction was left ot stir for 18 h. Then, the reaction mixture was filtered, diluted with EtOAc (50 mL), and washed with $NaHCO_3$ (1 × 20 mL), then saturated NaCl (1 × 20 mL). The organic layer was dried over Na_2SO_4 and the solvent was evaporated under reduced pressure. The crude product was purified by reverse phase flash chromatography, eluting with H_2O:ACN (0→100 %), gave the compound **13** (230 mg, 45% yield) as a white powder.

^{1}H NMR (400 MHz, Acetone-*d6*) δ 9.91 (s, 2H), 7.47 (dq, *J* = 3.6, 1.2 Hz, 2H), 7.39 – 7.34 (m, 2H), 7.27 – 7.20 (m, 6H), 7.18 – 7.12 (m, 1H), 6.82 – 6.77 (m, 4H), 6.25 (dd, *J* = 8.6, 5.9 Hz, 1H), 5.41 (s, 1H), 5.32 – 5.26 (m, 1H), 4.31 – 4.25 (m, 2H), 4.19 (s, 1H), 3.97 – 3.89 (m, 2H), 3.72 (d, *J* = 7.9 Hz, 1H), 3.67 (s, 7H), 3.48 (d, *J* = 14.7 Hz, 1H), 3.39 (dt, *J* = 10.6, 3.6 Hz, 1H), 3.28 (dd, *J* = 10.5, 3.0 Hz, 1H), 2.61 – 2.52 (m, 2H), 1.66 (d, *J* = 1.2 Hz, 3H), 1.31 (d, *J* = 1.2 Hz, 3H), 0.78 (s, 9H), 0.002 (s, 6H). ^{13}C NMR (101 MHz, Acetone-*d6*) δ 158.9, 144.8, 135.1, 134.5, 130.1, 130.0, 128.0, 127.9, 127.0, 113.2, 110.4, 109.2, 87.5, 86.9, 86.8, 84.2, 83.4, 81.5, 79.3, 71.9, 71.7, 63.6, 54.6, 40.4, 37.7, 25.1, 17.5, 11.7, 11.1, -5.5, -5.7. HRMS (m/z) $[M+H]^+$ calcd. for $C_{48}H_{59}N_5O_{14}SSiNa^+$, 1012.3458, found 1012.3441.

*(1R,3R,4R,7S)-1-((bis(4-methoxyphenyl)(phenyl)methoxy)methyl)-3-(5-methyl-2,4-dioxo-3,4-dihydropyrimidin-1(2H)-yl)-2,5-dioxabicyclo[2.2.1]heptan-7-yl (((1S,3R,4R,7S)-7-hydroxy-3-(5-methyl-2,4-dioxo-3,4-dihydropyrimidin-1(2H)-yl)-2,5-dioxabicyclo[2.2.1]heptan-1-yl)methyl)sulfamate **(14)***

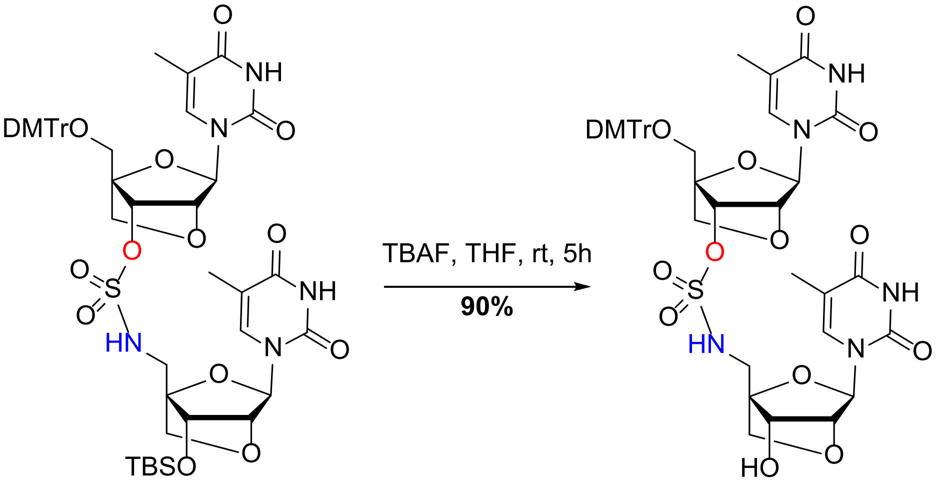

11 **14**

Chemical Formula: $C_{43}H_{45}N_5O_{15}S$
Exact Mass: 903.2633

A solution of 1 M TBAF in THF (404 uL, 0.404 mmol) was added to a solution of sulfamate dimer **11** (137 mg, 0.135 mmol) in dry THF (5 mL) and the resulting mixture was stirred for 5 h. EtOAc (30 mL) was then added, and the mixture was washed with 1 M aq. KH_2PO_4 (3 × 20 mL). The aqueous layers were then re-extracted with EtOAc (2 × 20 mL). The combined organic extracts were dried over Na_2SO_4 and the solvent was evaporated under reduced pressure. Purification by flash chromatography, eluting with 10% MeOH in EtOAc, gave compound **14** (110 mg, 90%) as a white foam.

^{1}H NMR (400 MHz, DMSO-*d6*) δ 11.55 (s, 1H), 11.38 (s, 1H), 7.56 (d, *J* = 1.3 Hz, 1H), 7.48 – 7.40 (m, 3H), 7.37 – 7.24 (m, 7H), 6.96 – 6.88 (m, 4H), 5.70 (d, *J* = 4.0 Hz, 1H), 5.59 (s, 1H), 5.44 (s, 1H), 4.88 (s, 1H), 4.71 (s, 1H), 4.17 (s, 1H), 3.89 – 3.82 (m, 2H), 3.75 (s, 8H), 3.67 (d, *J* = 8.0 Hz, 1H), 3.53 – 3.40 (m, 3H), 2.00 (s, 2H), 1.73 (d, *J* = 1.2 Hz, 3H), 1.55 (d, *J* = 1.2 Hz, 3H). ^{13}C NMR (151 MHz, DMSO-*d6*) δ 170.8, 164.2, 164.2, 158.7, 152.6, 150.4, 150.3, 144.9, 135.4, 135.2, 135.0, 134.2, 130.2, 130.1, 128.5, 128.0, 127.4, 113.8, 113.8, 109.4, 109.0, 87.1, 86.9, 86.7, 86.5, 85.5, 79.4, 77.2, 75.3, 72.0, 71.6, 70.3, 60.2, 58.1, 55.5, 34.9, 21.2, 14.5, 12.9, 12.9, 12.7, 12.7. HRMS (m/z) $[M+Na]^+$ calcd. for $C_{43}H_{45}N_5NaO_{15}S^+$, 926.2531, found 926.2525.

(2R,3S,5R)-2-((bis(4-methoxyphenyl)(phenyl)methoxy)methyl)-5-(5-methyl-2,4-dioxo-3,4-dihydropyrimidin-1(2H)-yl)tetrahydrofuran-3-yl(((1S,3R,4R,7S)-7-((tert-butyldimethylsilyl)oxy)-3-(5-methyl-2,4-dioxo-3,4-dihydropyrimidin-1(2H)-yl)-2,5-dioxabicyclo[2.2.1]heptan-1-yl)methyl)sulfamide ***(15)***

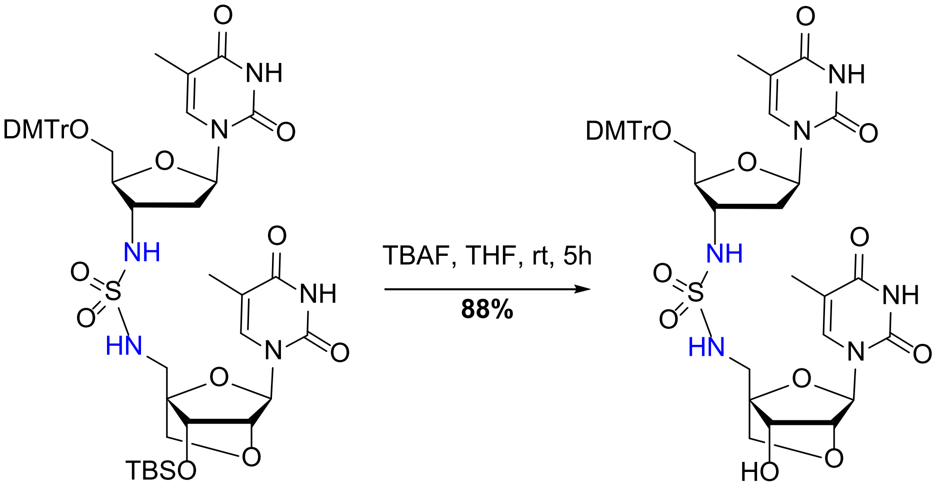

12 **15**

Chemical Formula: $C_{42}H_{46}N_6O_{13}S$
Exact Mass: 874.2844

A solution of 1 M TBAF in THF (1 mL, 1 mmol) was added to a solution of sulfamate dimer **12** (330 mg, 0.334 mmol) in dry THF (5 mL) and the resulting mixture was stirred for 5 h at room temperature. EtOAc (30 mL) was then added, and the mixture was washed with 1 M aq. KH_2PO_4 (3 × 20 mL). The aqueous layers were then re-extracted with EtOAc (2 × 20 mL) and the combined organic extracts were dried over Na_2SO_4 and the solvent was evaporated under reduced pressure. The crude residue was purified by flash chromatography, eluting with 10% MeOH in EtOAc, gave compound **15** (260 mg, 88%) as a white powder.

^{1}H NMR (400 MHz, DMSO-*d6*) δ 11.36 (s, 2H), 7.58 – 7.48 (m, 4H), 7.39 (dt, *J* = 6.5, 1.3 Hz, 2H), 7.34 – 7.21 (m, 7H), 6.93 – 6.85 (m, 4H), 6.20 (t, *J* = 6.4 Hz, 1H), 5.59 (d, *J* = 4.2 Hz, 1H), 5.34 (s, 1H), 4.12 (s, 1H), 3.99 (d, *J* = 3.7 Hz, 1H), 3.87 (d, *J* = 4.3 Hz, 1H), 3.81 (d, *J* = 8.0 Hz, 1H), 3.74 (s, 6H), 3.69 (d, *J* = 7.9 Hz, 1H), 3.30 – 3.12 (m, 4H), 2.49 – 2.30 (m, 2H), 1.72 (d, *J* = 1.1 Hz, 3H), 1.40 (d, *J* = 1.2 Hz, 3H). ^{13}C NMR (151 MHz, DMSO-*d6*) δ 170.8, 164.2, 164.1, 158.6, 158.6, 150.7, 150.3, 145.2, 136.0, 135.8, 135.7, 135.2, 130.2, 130.1, 128.3, 128.1, 127.2, 113.7, 113.7, 109.9, 108.9, 87.0, 86.8, 86.4, 84.3, 83.0, 79.3, 71.8, 70.3, 63.70, 60.2, 55.5, 55.5, 53.2, 38.4, 35.0, 21.2, 14.5, 12.6, 12.5, 11.5. HRMS (m/z) $[M+H]^+$ calcd. for $C_{42}H_{46}N_6O_{13}SNa^+$, 897.2736, found 897.2743.

*(2R,3S,5R)-2-((bis(4-methoxyphenyl)(phenyl)methoxy)methyl)-5-(5-methyl-2,4-dioxo-3,4-dihydropyrimidin-1(2H)-yl)tetrahydrofuran-3-yl (((1S,3R,4R,7S)-7-hydroxy-3-(5-methyl-2,4-dioxo-3,4-dihydropyrimidin-1(2H)-yl)-2,5-dioxabicyclo[2.2.1]heptan-1-yl)methyl)sulfamate **(16)***

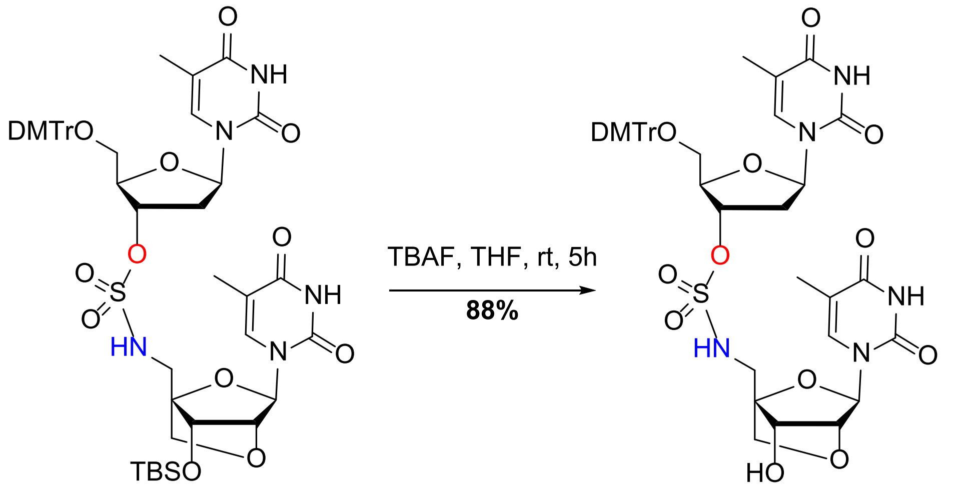

A solution of 1 M TBAF in THF (0.697 mL, 0.69 mmol) was added to a solution of sulfamate dimer **13** (230 mg, 0.232 mmol) in dry THF (5 mL) and the resulting mixture was stirred for 5 h at room temperature. EtOAc (30 mL) was then added, and the mixture was washed with 1 M aq. KH_2PO_4 (3 × 20 mL). The aqueous layers were then re-extracted with EtOAc (2 × 20 mL) and the combined organic extracts were dried over Na_2SO_4 and the solvent was evaporated under reduced pressure. The crude produce was purified by flash chromatography, eluting with 10% MeOH in EtOAc, gave compound **16** (180 mg, 88%) as a white powder.

^{1}H NMR (400 MHz, DMSO-*d6*) δ 11.41 (s, 1H), 11.37 (s, 1H), 7.49 (dd, *J* = 2.2, 1.2 Hz, 2H), 7.40 – 7.35 (m, 2H), 7.32 (t, *J* = 7.6 Hz, 2H), 7.24 (ddd, *J* = 9.0, 4.3, 2.1 Hz, 5H), 6.90 (dt, *J* = 9.0, 1.7 Hz, 4H), 6.22 (dd, *J* = 8.3, 6.1 Hz, 1H), 5.72 (d, *J* = 4.1 Hz, 1H), 5.35 (s, 1H), 5.23 (dt, *J* = 5.1, 2.3 Hz, 1H), 4.28 (q, *J* = 3.1 Hz, 1H), 4.12 (s, 1H), 3.91 – 3.83 (m, 2H), 3.74 (s, 7H), 3.70 – 3.61 (m, 1H), 3.44 – 3.36 (m, 1H), 3.12 (dd, *J* = 10.6, 3.0 Hz, 1H), 2.62 – 2.54 (m, 2H), 1.71 (d, *J* = 1.2 Hz, 3H), 1.37 (d, *J* = 1.2 Hz, 3H). ^{13}C NMR (151 MHz, DMSO-*d6*) δ 170.8, 164.2, 164.0, 158.7, 158.7, 150.8, 150.3, 145.0, 135.8, 135.6, 135.4, 135.1, 130.2, 130.1, 128.4, 128.1, 128.0, 127.3, 113.8, 113.8, 110.4, 109.0, 86.8, 86.7, 86.7, 84.2, 83.0, 81.0, 79.5, 71.7, 70.4, 63.8, 60.2, 55.5, 55.5, 37.5, 21.2, 14.5, 12.5, 12.5, 12.0, 12.0. HRMS (m/z) $[M+H]^+$ calcd. for $C_{42}H_{45}N_5O_{14}SNa^+$, 898.2576, found 898.2599.

(1R,3R,4R,7S)-1-((bis(4-methoxyphenyl)(phenyl)methoxy)methyl)-3-(5-methyl-2,4-dioxo-3,4-dihydropyrimidin-1(2H)-yl)-2,5-dioxabicyclo[2.2.1]heptan-7-yl(((1S,3R,4R,7S)-7-(((2-cyanoethoxy)(diisopropylamino)phosphaneyl)oxy)-3-(5-methyl-2,4-dioxo-3,4-dihydropyrimidin-1(2H)-yl)-2,5-dioxabicyclo[2.2.1]heptan-1-yl)methyl)sulfamate **(17)**

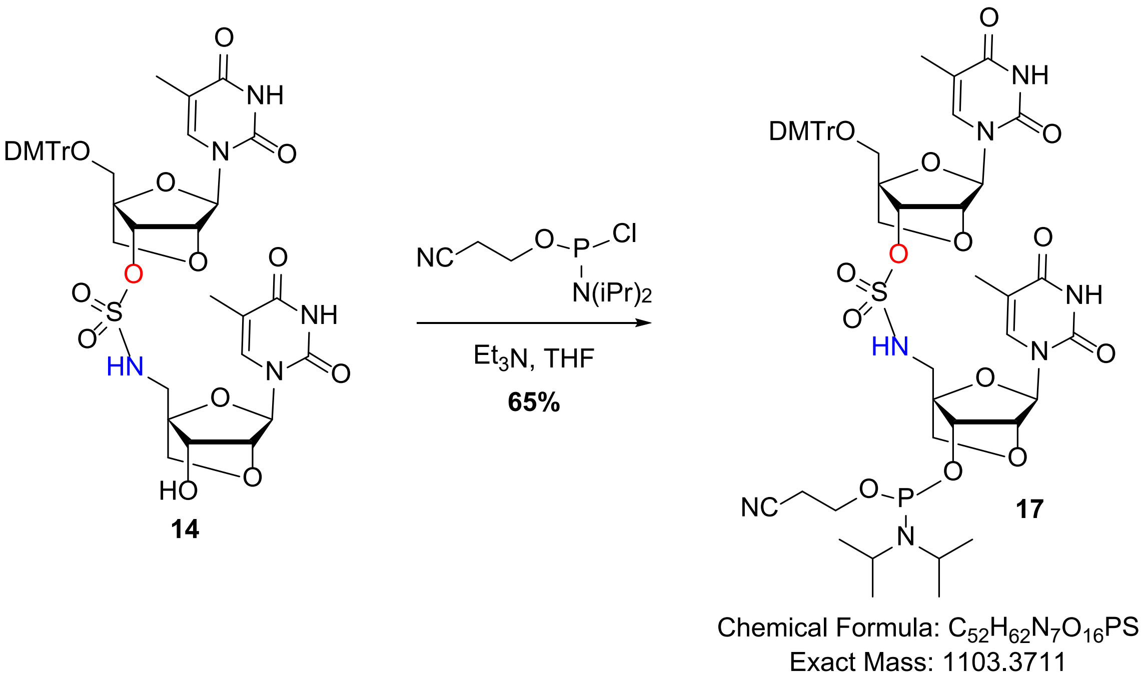

To a solution of dimer **14** (100 mg, 0.111 mmol) in dry degassed THF (5 mL) was added dry degassed Et_3N (61.7 µL, 0.443 mmol) followed by chloro (diisopropylamino)-*β*-cyanoethoxyphosphine (49.4 µL, 0.221 mmol). After stirring at room temperature under argon for 3 hours the reaction mixture was diluted with degassed CH_2Cl_2 (5.0 mL), washed with sat. aq KCl (5 mL) and dried over Na_2SO_4. The organic layer was concentrated under reduced pressure and the crude product was purified by silica gel column chromatography (degassed 10% MeOH in EtOAc) to give phosphoramidite **17** (80 mg, 0.072 mmol, 65%) as a white solid.

^{31}P NMR (162 MHz, CD_3CN) δ 149.0, 148.8. HRMS (m/z) $[M+Na]^+$ calcd. for $C_{52}H_{62}N_7O_{16}PSNa^+$, 1126.3609, found 1126.3634.

*(1S,3R,4R,7S)-1-(((N-((2S,3S,5R)-2-((bis(4-methoxyphenyl)(phenyl)methoxy)methyl)-5-(5-methyl-2,4-dioxo-3,4-dihydropyrimidin-1(2H)-yl)tetrahydrofuran-3-yl)sulfamoyl)amino)methyl)-3-(5-methyl-2,4-dioxo-3,4-dihydropyrimidin-1(2H)-yl)-2,5-dioxabicyclo[2.2.1]heptan-7-yl (2-cyanoethyl) diisopropylphosphoramidite **(18)***

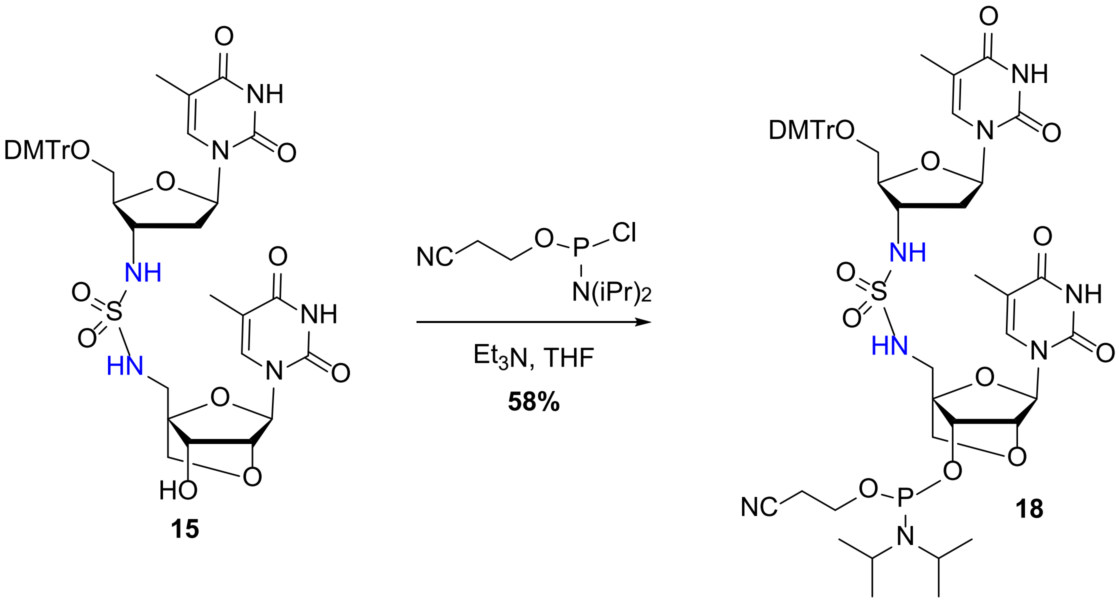

Chemical Formula: $C_{51}H_{63}N_8O_{14}PS$
Exact Mass: 1074.3922

To a solution of dimer **15** (250 mg, 0.286 mmol) in dry degassed THF (5 mL) was added dry degassed Et_3N (119 µL, 0.857 mmol) followed by chloro(diisopropylamino)-*β*-cyanoethoxyphosphine (95.6 µL, 0.429 mmol). The reaction was stirred at room temperaure under argon for 3 hours then diluted with degassed CH_2Cl_2 (5 mL), washed with sat. aq KCl (5 mL) and dried over Na_2SO_4. The organic layer was concentrated under reduced pressure and the crude product was purified by silica column chromatography (degassed 10% MeOH in EtOAc) to give phosphoramidite **18** (180 mg, 0.167 mmol, 58% yield) as a white solid.

^{31}P NMR (162 MHz, CD_3CN) δ 148.9, 148.7. HRMS (m/z) $[M+H]^+$ calcd. for $C_{51}H_{64}N_8O_{14}PS^+$, 1075.3995, found 1075.4025.

(2R,3S,5R)-2-((bis(4-methoxyphenyl)(phenyl)methoxy)methyl)-5-(5-methyl-2,4-dioxo-3,4-dihydropyrimidin-1(2H)-yl)tetrahydrofuran-3-yl (((1S,3R,4R,7S)-7-(((2-cyanoethoxy)(diisopropylamino)phosphaneyl)oxy)-3-(5-methyl-2,4-dioxo-3,4-dihydropyrimidin-1(2H)-yl)-2,5-dioxabicyclo[2.2.1]heptan-1-yl)methyl)sulfamate ***(19)***

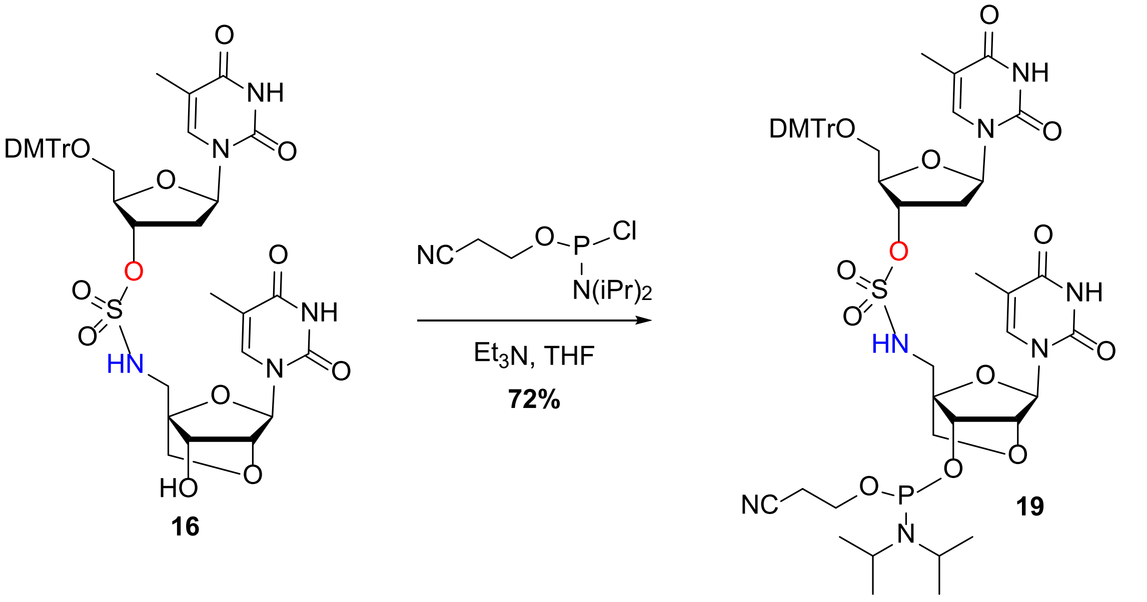

To a solution of dimer **16** (260 mg, 0.297 mmol) in dry degassed THF (5 mL) was added dry degassed Et_3N (124 µL, 0.891 mmol) followed by chloro(diisopropylamino)-*β* -cyanoethoxyphosphine (99.3 µL, 0.445 mmol).The reaction mixture was stirred at rt under argon for 3 hours, diluted with degassed CH_2Cl_2 (5 mL), washed with sat. aq KCl (5 mL) and dried over Na_2SO_4. The organic layer was concentrated under reduced pressure and the crude product was purified by silica column chromatography (degassed 10% MeOH in EtOAc) to give phosphoramidite **19** (230 mg, 0.213 mmol, 72% yield) as a white solid.

^{31}P NMR (162 MHz, CD_3CN) δ 149.1, 148.9. HRMS (m/z) $[M+H]^+$ calcd. for $C_{51}H_{63}N_7O_{15}PS^+$, 1076.3835, found 1076.3855.

4. Synthesis of oligonucleotides

I. 2'OMe Phosphorothioate oligonucleotide synthesis

2'OMe oligonucleotides were synthesised on an Applied Biosystems 394 automated DNA/RNA synthesiser using a standard phosphoramidite cycle of detritylation, coupling, oxidation and capping, on the 1.0 µmole scale. Regents for detritylation (TCA), coupling (BTT), oxidation (iodine) and capping (acetic anhydride and N-methylimidazole), are identical to those used for standard oligonucleotide DNA synthesis and were purchased from Sigma-Aldrich. In case of phosphorothioate oligonucleotides synthesis, sulphurising Reagent, EDITH (LK2171) from Biosearch Technologies, was used for sulphurisation (4 min).

Pre-packed nucleoside SynBase™ CPG 1000/110 synthesis resin (Link Technologies and Glen Research Twist) was used, and β-cyanoethyl phosphoramidite monomers (5'-DMT-2'O-methyl-rA(Bz), 5'-DMT-2'O-methyl-rG(iBu), 5'-DMT-2'O-methyl-rC(Ac) and 5'-DMT-2'O-Methyl-rU, Sigma-Aldrich) were dissolved in anhydrous MeCN to a concentration of 0.1 M immediately prior to use with a coupling time of 6 min. The modified dinucleotide phosphoramidites were dissolved at the same concentration and coupled for 8 minutes. Stepwise coupling efficiencies were determined by automated trityl cation conductivity monitoring and were >98% in all cases. The DMT group was kept on in the solid support after the last coupling and was removed after deprotection and purification of the oligonucleotides by HPLC.

II. Deprotection from the solid support

Condition A: Cleavage and deprotection were achieved by treatment with 0.5 mL of THF and 0.5 mL of ethylenediamine for 3 h room temperature. The mixture of THF and ethylenediamine was discarded and then the resin was washed with 1 mL of DNase free water to dissolve the crude DMT-ON oligonucleotides.

Condition B: Cleavage and deprotection were performed by treatment with concentrated aqueous ammonia at 55 ℃ for 5 h in a screw-capped vial. The reaction was allowed to cool to room temperature and was concentrated on a rotary evaporator. The crude DMT-ON oligonucleotide was dissolved in water and lyophilised.

Condition C: Cleavage and deprotection were performed by treatment with concentrated aqueous ammonia at rt for 24 h in a capped vial and work-up was asdescribed above

III. *Purification of oligonucleotides*

Oligonucleotides were purified using a Gilson reverse-phase high performance liquid chromatography (RP HPLC) system with an ACE® C8 column (particle size: 10 μm, pore size: 100 Å, column dimensions: 10 mm x 250 mm) with a gradient of buffer A (0.1 M triethylammonium bicarbonate (TEAB), pH 7.5) to buffer B (0.1 M TEAB, pH 7.5 containing 50% v/v MeCN) and flow rate of 4 mL/min. The gradient was increased from 0% to 100% buffer B over 30 minutes (Condition A). Elution was monitored by UV absorbance at 295 nm. After HPLC purification, oligonucleotides were freeze dried then dissolved in water without the need for desalting.

Table S1. Analysis of deprotection of 5´-DMT oligonucleotides

**Oligonucleotide**	**Sequence (5´→3´)**	**Deprotection**	**MS Calc.**	**MS Obs.**	**Remarks**
**ON1**<br>LNA-sulfamate-LNA	DMT-CCUC^{L}T$_{x1}$^{L}TACCUCAG^{L}T$_{x1}$^{L}TACA	EDA, THF, 3h	6413.32	6413.00	✓
		NH_3, 55 °C, 5h	6413.32	6413.00	✓
**ON2**<br>DNA-sulfamide-LNA	DMT-CCUCT$_{x2}$^{L}TACCUCAGT$_{x2}$^{L}TACA	EDA, THF, 3h	6355.34	6401.00	Acetylated backbone
		EDA, THF, 3h	6355.34	6443.00	Acetylated backbone
		NH_3, 55 °C, 5h	6355.34	6357.00	✓
ON3<br>DNA-sulfamide-LNA	DMT-CCUCT$_{x2}$^{L}TACCUCAGUUACA	EDA, THF, 3h	6376.91	6420.50	Acetylated backbone
		NH_3, 55 °C, 5h	6376.31	6381.00	✓
ON4<br>DNA-sulfamate-LNA	DMT-CCUCT$_{x1}$^{L}TACCUCAGT$_{x1}$^{L}TACA	EDA, THF, 3h	6357.32	--	Decomposition
		NH_3, rt, 24h	6357.32	--	Decomposition
		NH_3, 55 °C 5h, 24h	6357.32	--	Decomposition
		NH_3, rt, 24h, NC*	6357.32	6360.60	✓
		EDA, THF, 3h, NC*	6357.32	6360.60	✓
**ON5**<br>DNA-sulfamate-LNA	DMT-CCUCT$_{x1}$^{L}TACCUCAGUUACA	EDA/THF, 3h	6377.90	4554.00	S-O bond cleavage
		NH_3, 55 °C 5h, 24h	6377.90	4554.00	S-O bond cleavage
		NH_3, rt, 24h	6377.90	4554.00	S-O bond cleavage
		NH_3, rt, 24h, NC*	6377.90	6381.40	✓
		EDA, THF, 3h, NC*	6377.90	6381.40	✓

IV. *Deprotection of the pure DMT-ON oligonucleotides*

The DMT-ON oligonucleotides were then dissolved in 100 μL of 80% acetic acid and left for 30 mins at room temperature to remove the DMT group. The solution was neutralised with 300 μL of triethylammonium acetate buffer (2 M, pH 7) and the detritylated oligonucleotides were desalted using a NAP-10 column (Cytiva) according to the manufacturer's instructions. The crude oligonucleotides were lyophilised.

Table S2. Deprotection of the **DMTOFF** group from the **DMTON oligonucleotides**.

ON	Sequence (5'→3')	MS Cald	MS Obs	Purity
**ON1**	CCU C^{L}T$_{X1}$^{L}T ACC UCA G^{L}T$_{X1}$^{L}TACA	6111.18	6113.00	92%
**ON2**	CCU CT$_{X2}$^{L}T ACC UCA GT$_{X2}$^{L}T ACA	6053.20	6053.20	93%
**ON3**	CCU CT$_{X2}$^{L}T ACC UCA GUU ACA	6074.77	6076.50	97%
**ON2-Ac**^{l}	CCU CT$_{X2,Ac}$^{L}T ACC UCA GT$_{X2,Ac}$^{L}T ACA	6095.24	6097.00	--
**ON3-Ac**	CCU CT$_{X2,Ac}$^{L}T ACC UCA GUU ACA	6116.81	6117.00	--
**ON4**	CCU CT $_{X1}$^{L}T ACC UCA GT $_{X1}$^{L}T ACA	6055.18	6055.60	(53% [a])
**ON5**	CCU CT $_{X1}$^{L}T ACC UCA GUU ACA	6075.76	6075.70	(48%[a])

[a]Purity was calculated by integrating the gel bands obtained from the nuclease stability studies with Image J software. The samples were heated at 95 ℃ in aqueous buffer to denature them before loading the gel, leading to decomposition of ON4 and ON5 at the sulfamate linkages. The actual purity of these oligonucleotides (unheated) is high as can be seen from the HPLC/MS analyses (Figures S11 to S14)

V. *Oligonucleotide analysis*

All oligonucleotides were characterised by negative-mode ultra-performance liquid chromatography (UPLC) mass spectrometry using a Waters Xevo G2-XS QT of mass spectrometer with an Acquity UPLC system. The system is equipped with an Acquity UPLC oligonucleotide BEH C18 column (particle size: 1.7 µm; pore size: 130 Å; column dimensions: 2.1 mm x 50 mm). Data were analysed using Waters MassLynx software v 4.1 or Waters UNIFI Scientific Information System software.

5. UPLC and Mass Spectra of the Oligonucleotides

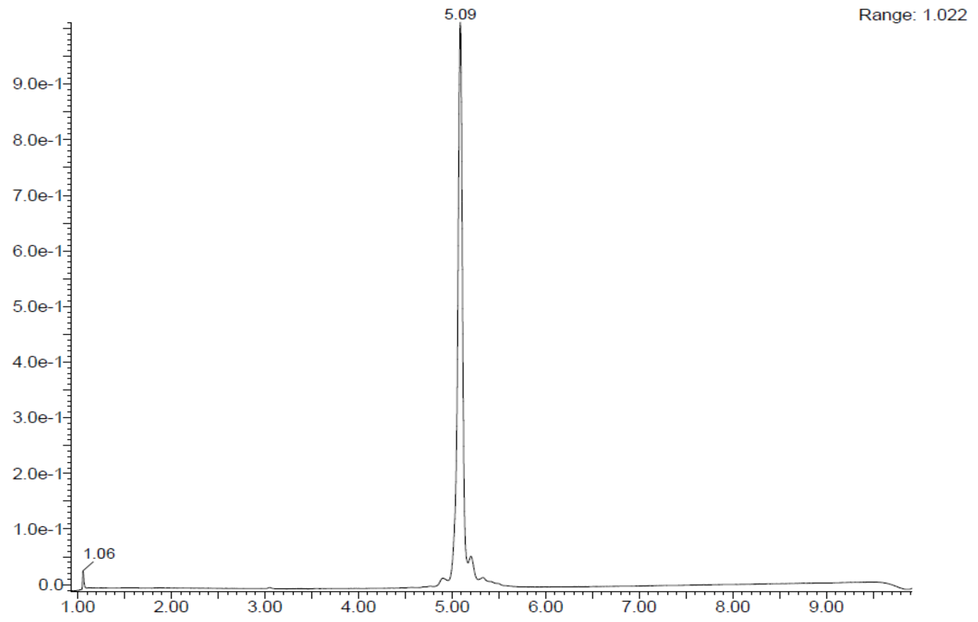

Supplementary Figure S1. Reverse-phase UPLC of crude **ON1** after DMT removal before purification (UV absorbance at 260 nm vs time in min)

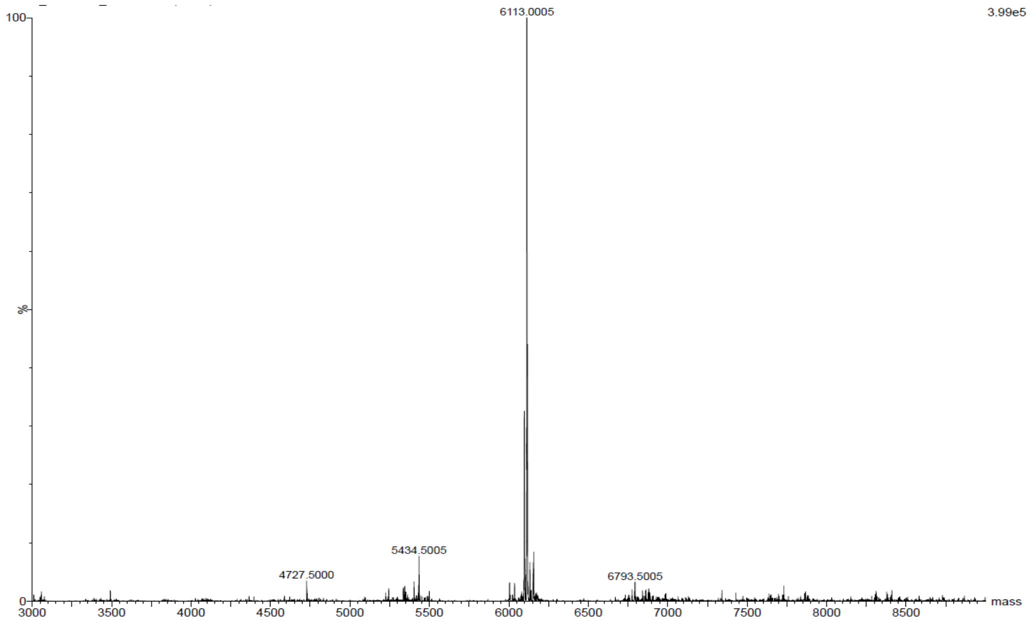

Supplementary Figure S2. Mass spectrum (ES-) of crude **ON1** after DMT removal without purification. Required **6111.18** Da, found **6113.00** Da. y-axis = relative intensity (%), x-axis = mass in Da.

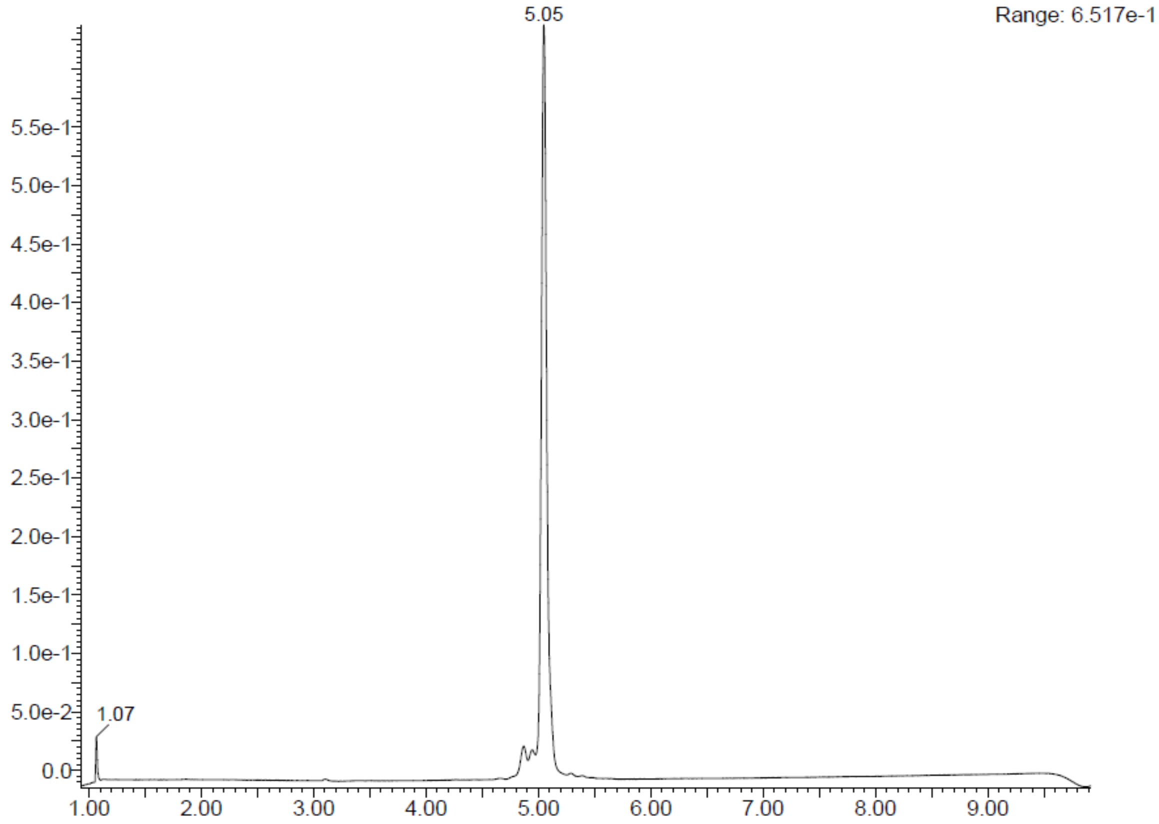

Supplementary Figure S3. Reverse-phase UPLC crude **ON2** after DMT removal before purification (UV absorbance at 260 nm vs time in min).

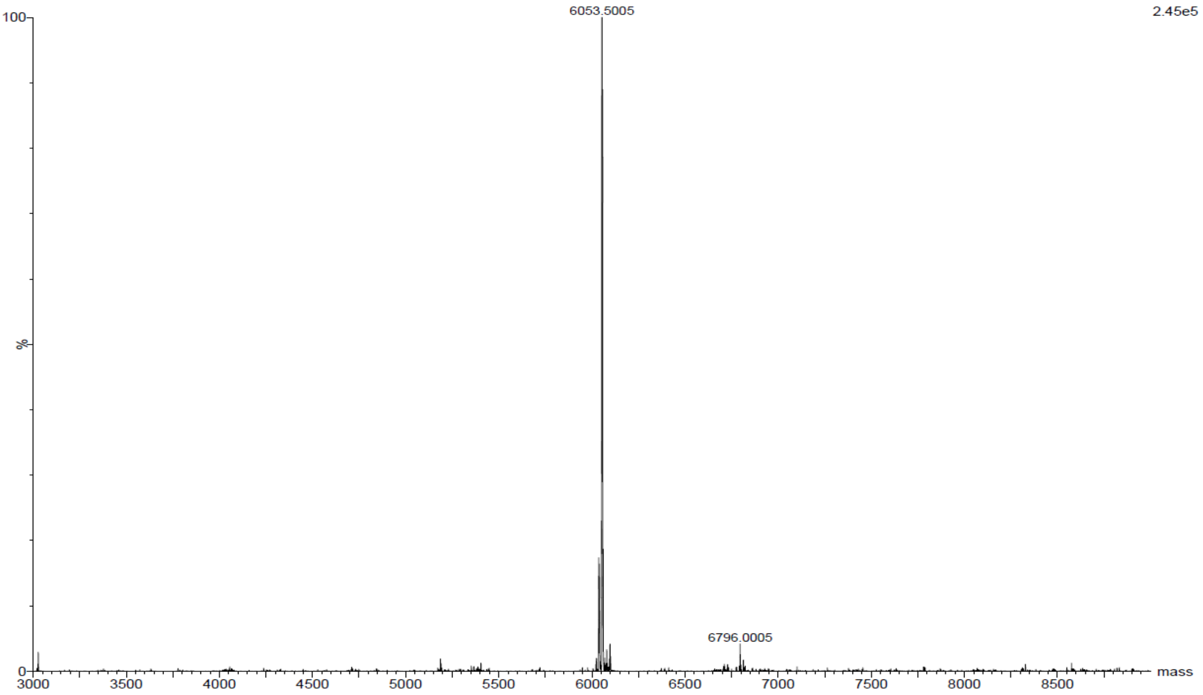

Supplementary Figure S4. Mass spectrum (ES-) of crude **ON2** after DMT removal before purification. Required **6053.20** Da, found **6053.50** Da. y-axis = relative intensity (%), x-axis = mass in Da.

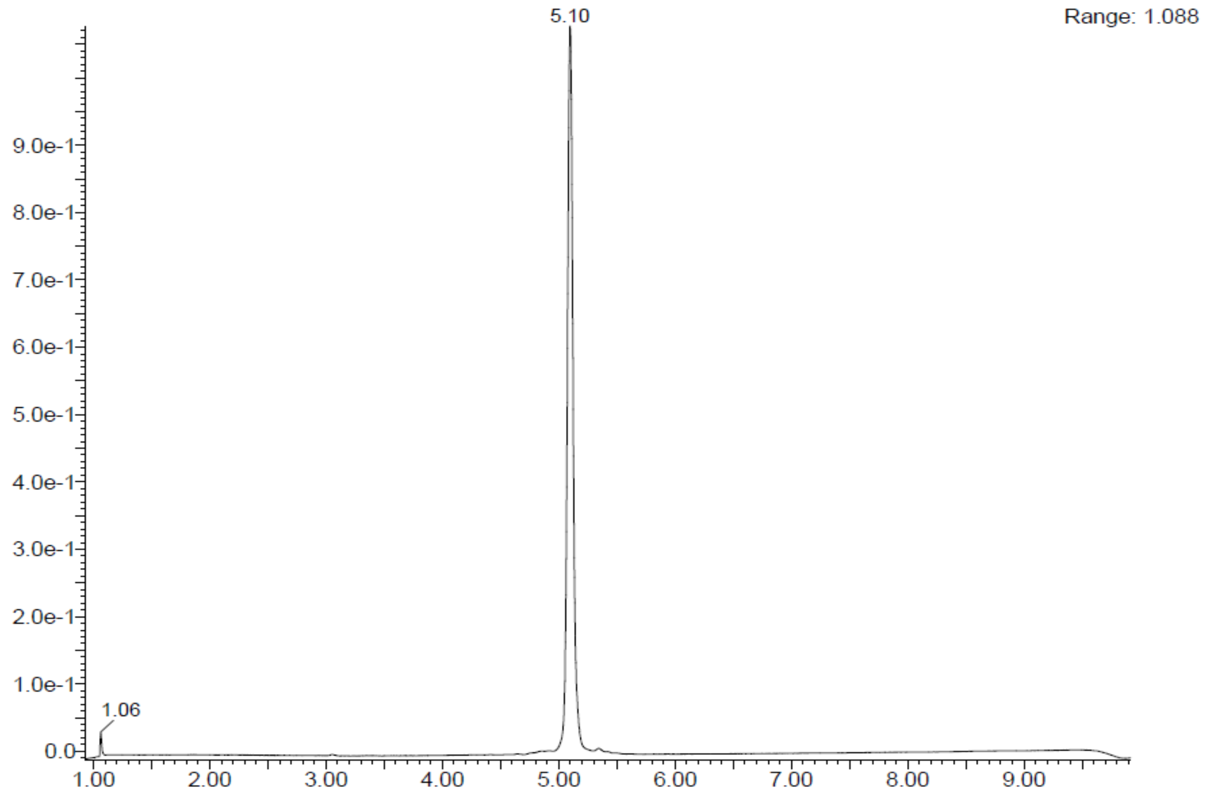

Supplementary Figure S5. Reverse-phase UPLC of crude **ON3** after DMT removal before purification (UV absorbance at 260 nm vs time in min)

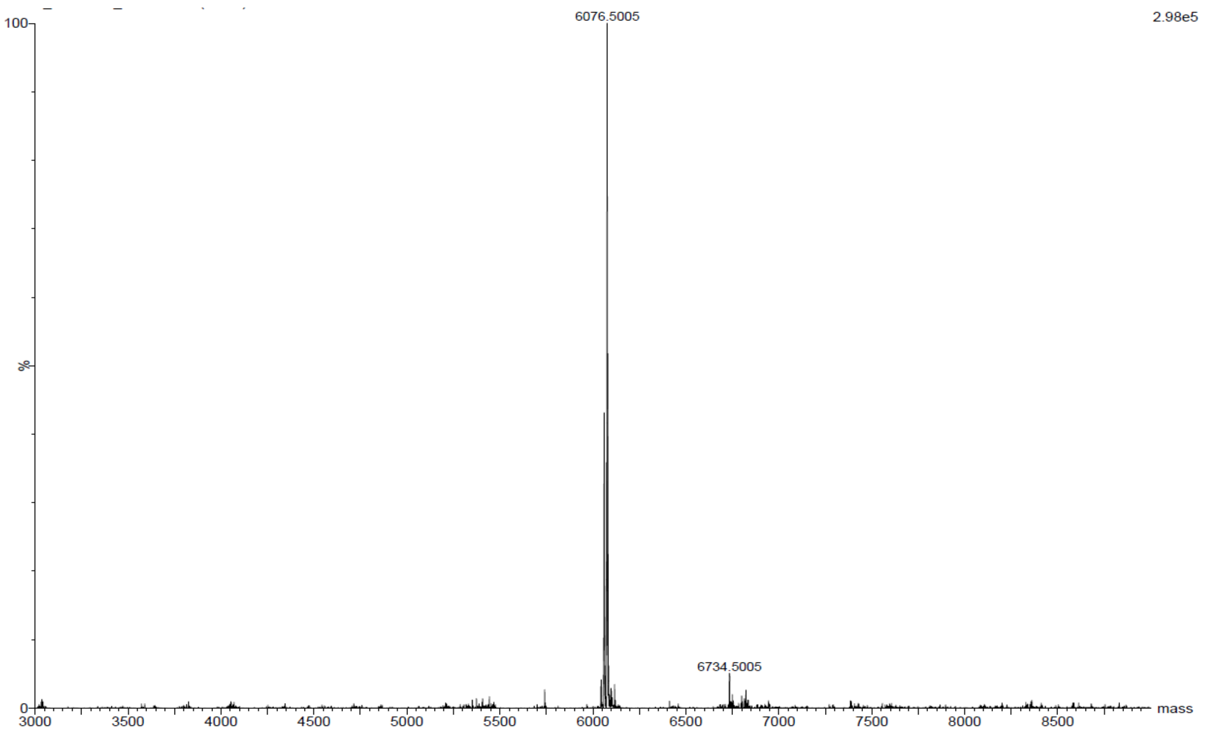

Supplementary Figure S6. Mass spectrum (ES-) of crude **ON3** after DMT removal before purification. Required **6074.77** Da, found **6076.50** Da. y-axis = relative intensity (%), x-axis = mass in Da.

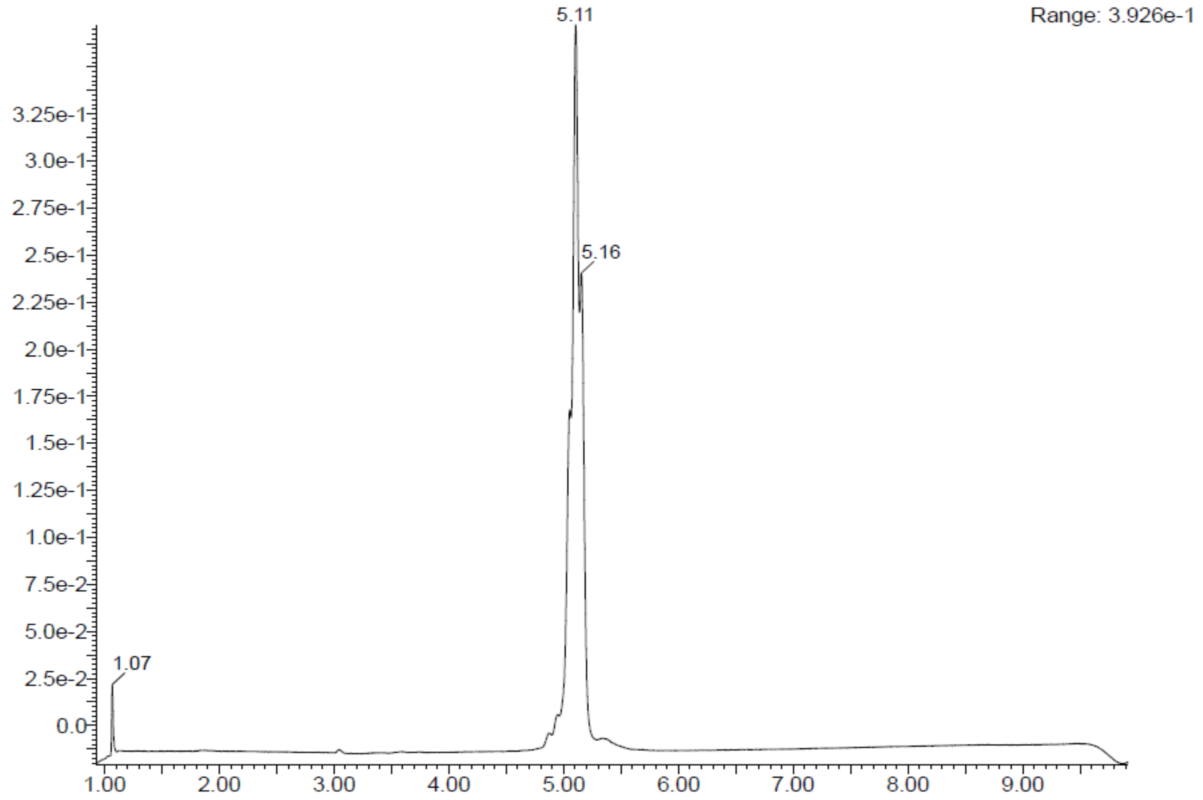

Supplementary Figure S7. Reverse-phase UPLC crude **ON2_Ac** after DMT removal without purification (UV absorbance at 260 nm vs time in min).

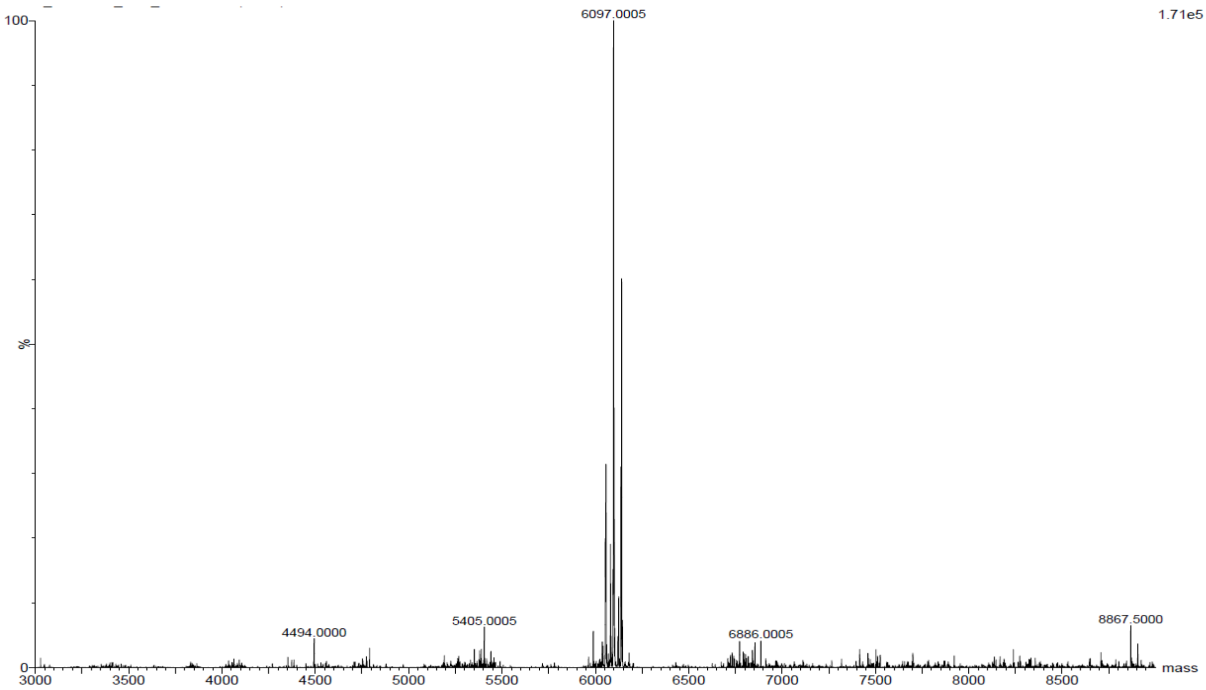

Supplementary Figure S8. Mass spectrum (ES-) of crude **ON2_Ac** after DMT removal without purification. Required **6095.24** and **6137.28**, found **6097.00** and **6139.00** Da. y-axis = relative intensity (%), x-axis = mass in Da.

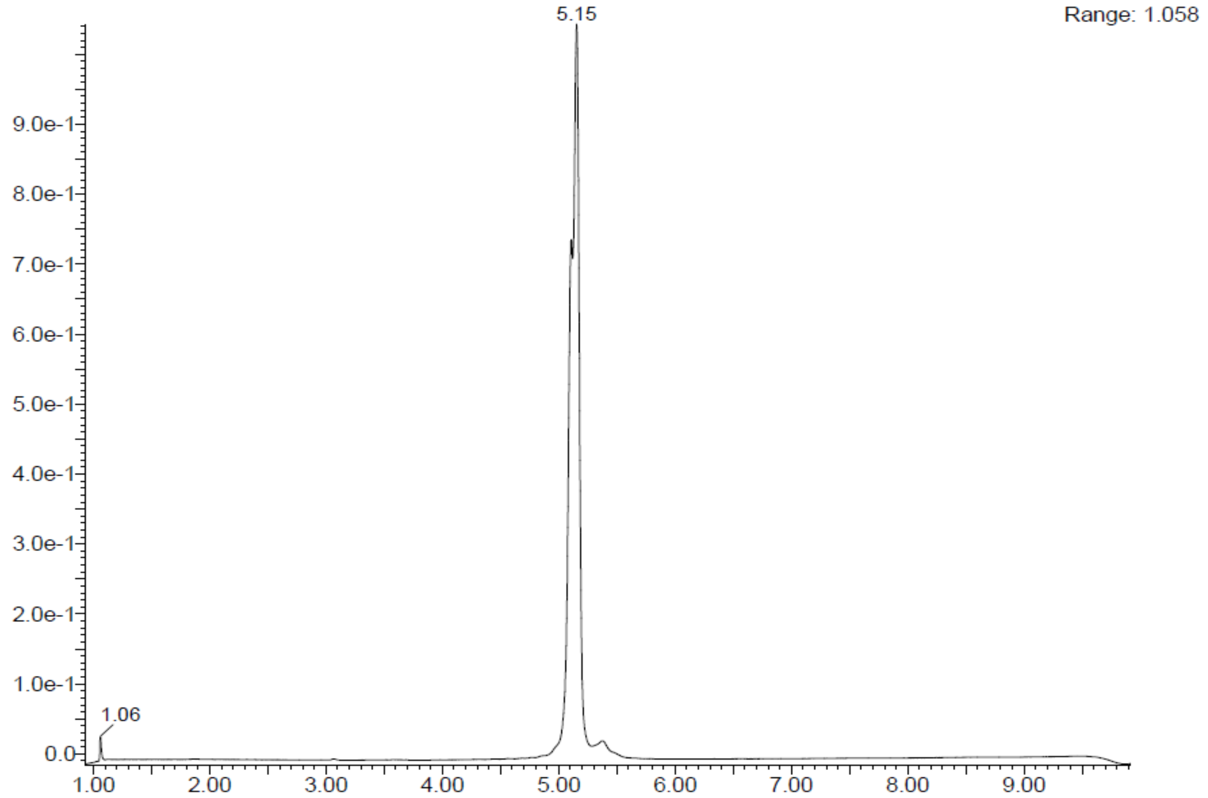

Supplementary Figure S9. Reverse-phase UPLC crude **ON3_Ac** after DMT removal without purification (UV absorbance at 260 nm vs time in min)

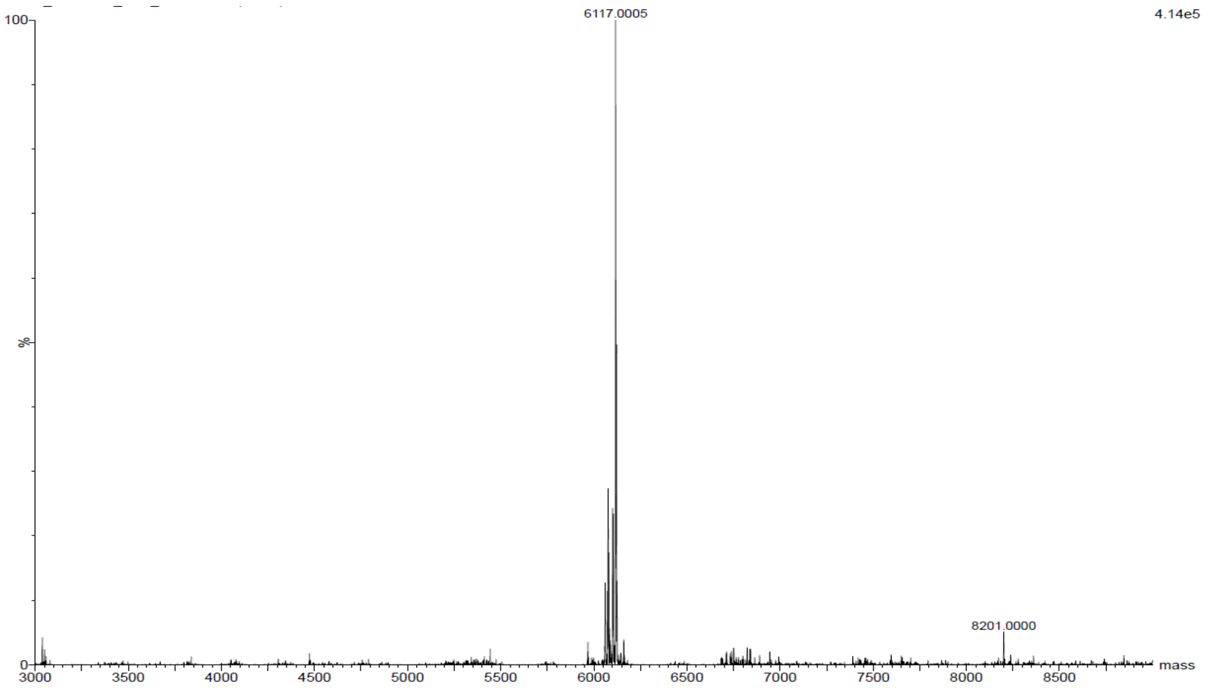

Supplementary Figure S10. Mass spectrum (ES-) of crude **ON3_Ac** after DMT removal without purification. Required **6116.81** Da, found **6117.00** Da. y-axis = relative intensity (%), x-axis = mass in Da.

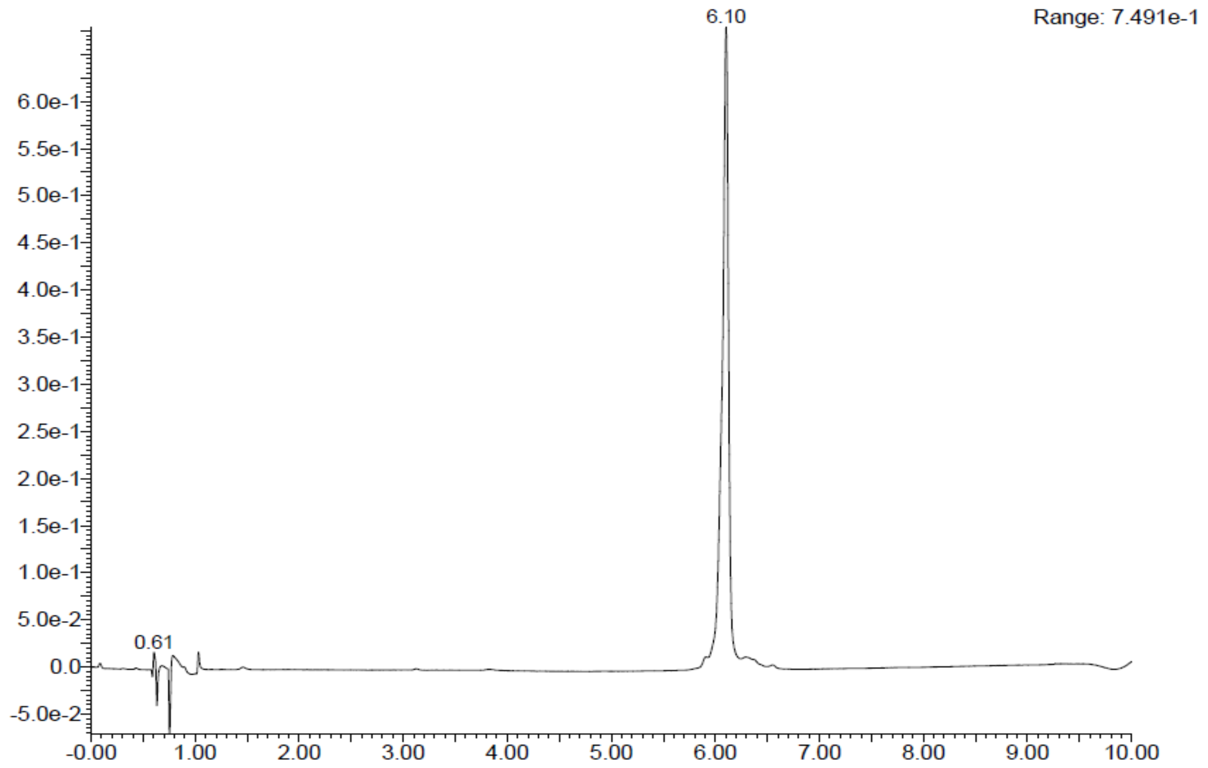

Supplementary Figure S11: Reverse-phase UPLC of **ON4** after DMT group removal followed by purification by HPLC (UV absorbance at 260 nm vs time in min)

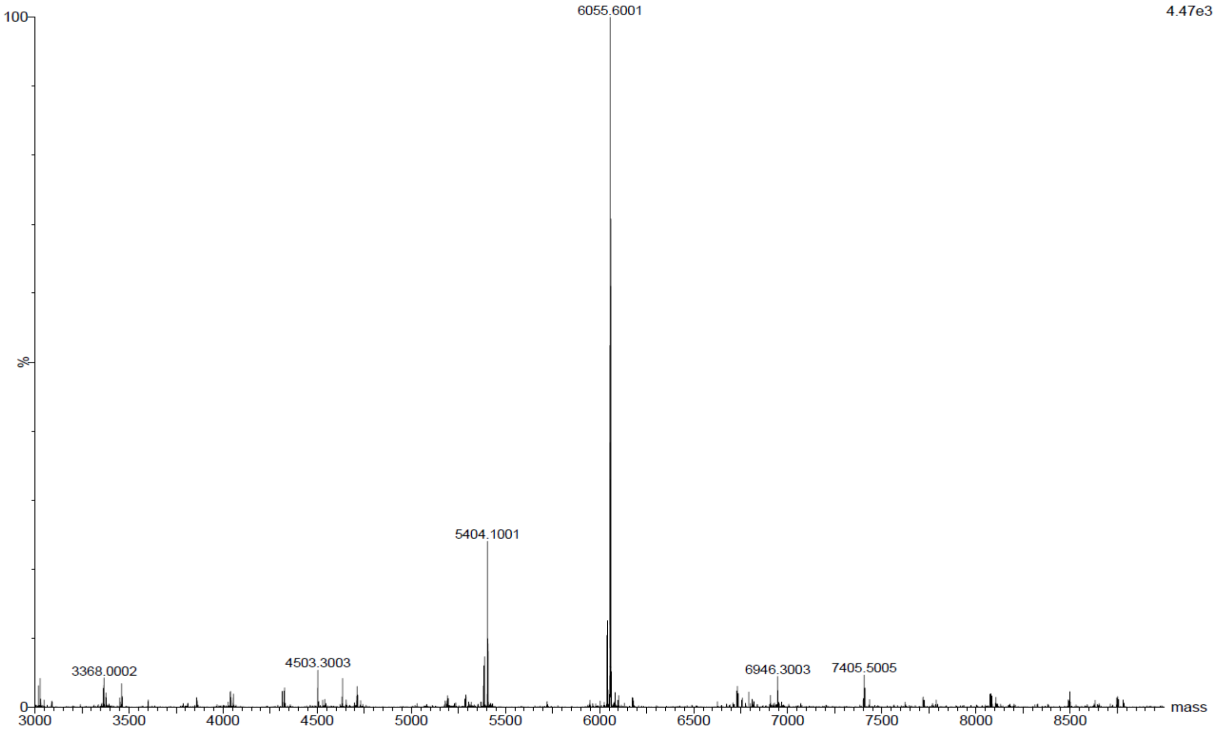

Supplementary Figure S12: Mass spectrum (ES-) of **ON4**. Required **6055.18** Da, found **6055.60** Da. y-axis = relative intensity (%), x-axis = mass in Da.

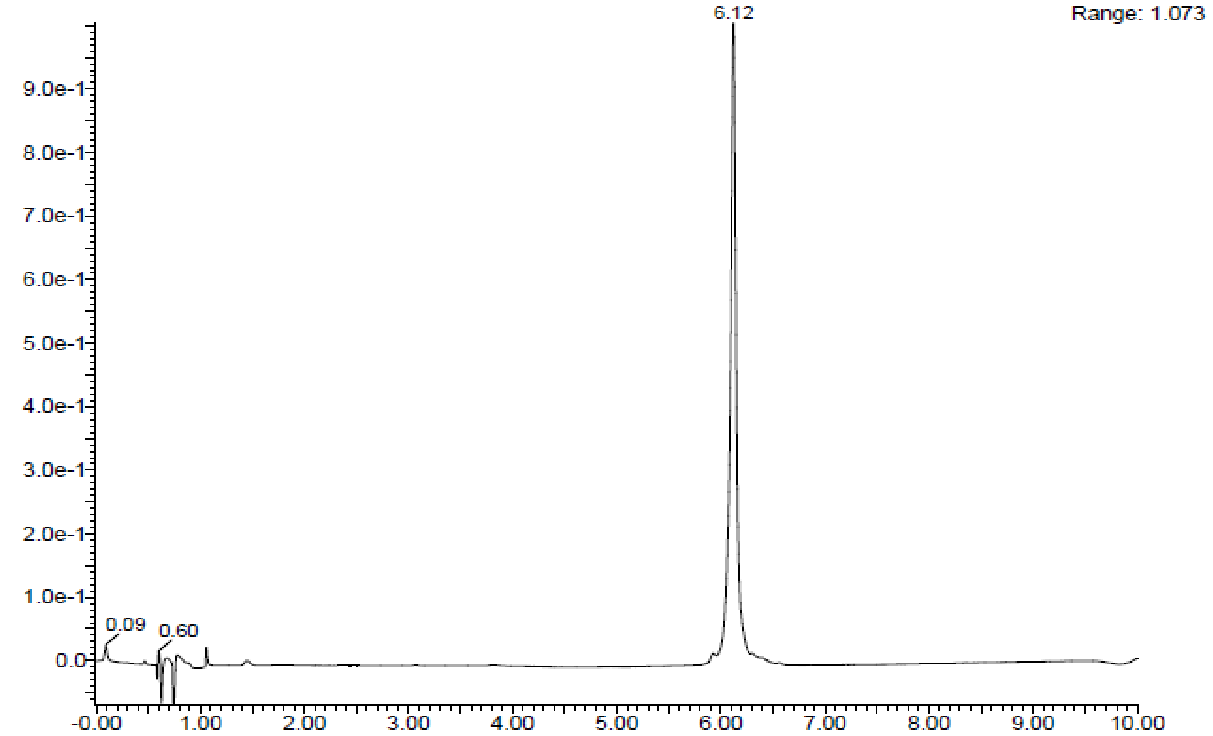

Supplementary Figure S13: Reverse-phase UPLC of **ON5** after DMT grooup removal followed by purification by HPLC (UV absorbance at 260 nm vs time in min)

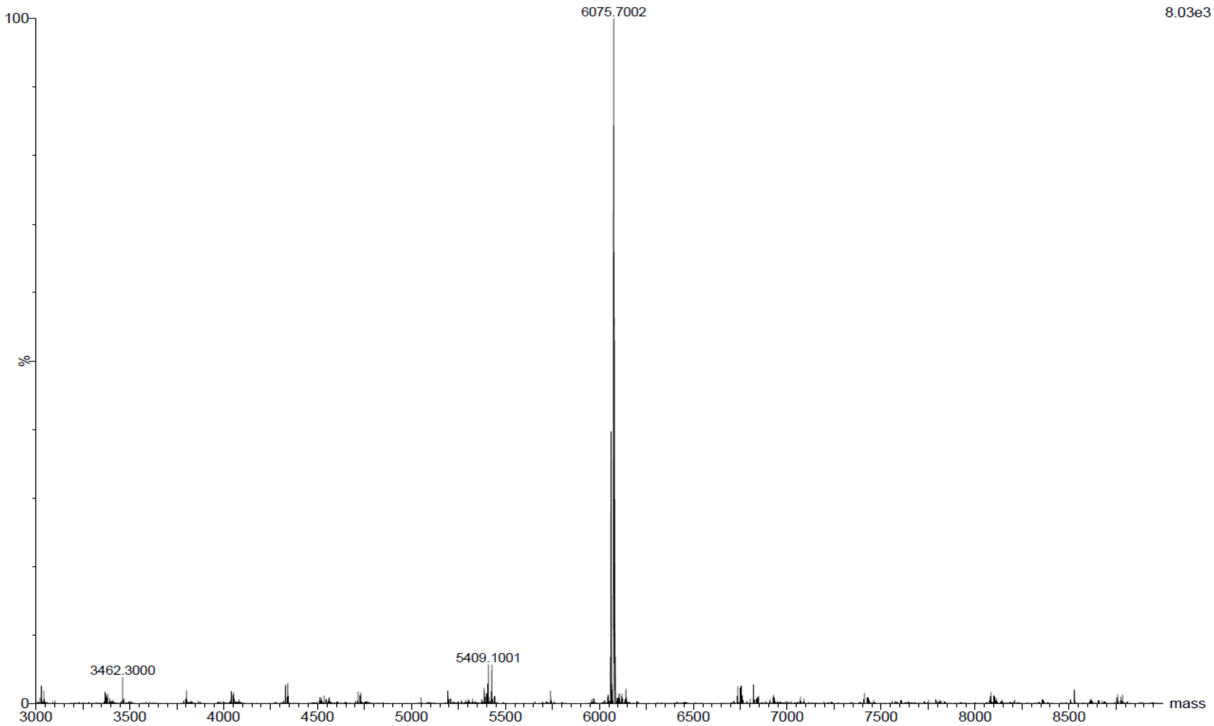

Supplementary Figure S14: Mass spectrum (ES-) of **ON5**. Required **6075.26** Da, found **6075.70** Da. y-axis = relative intensity (%), x-axis = mass in Da.

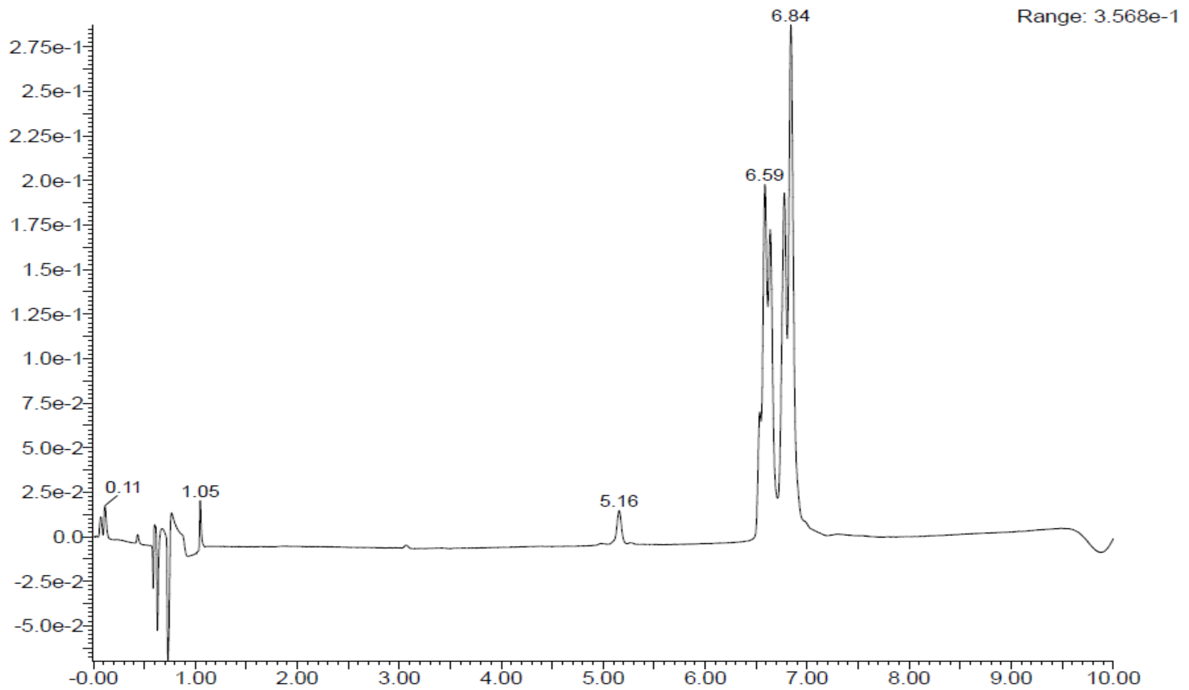

Supplementary Figure S15: Reverse-phase UPLC of **DMT-ON ON1** after deprotection from solid support followed by purification by HPLC (UV absorbance at 260 nm vs time in min)

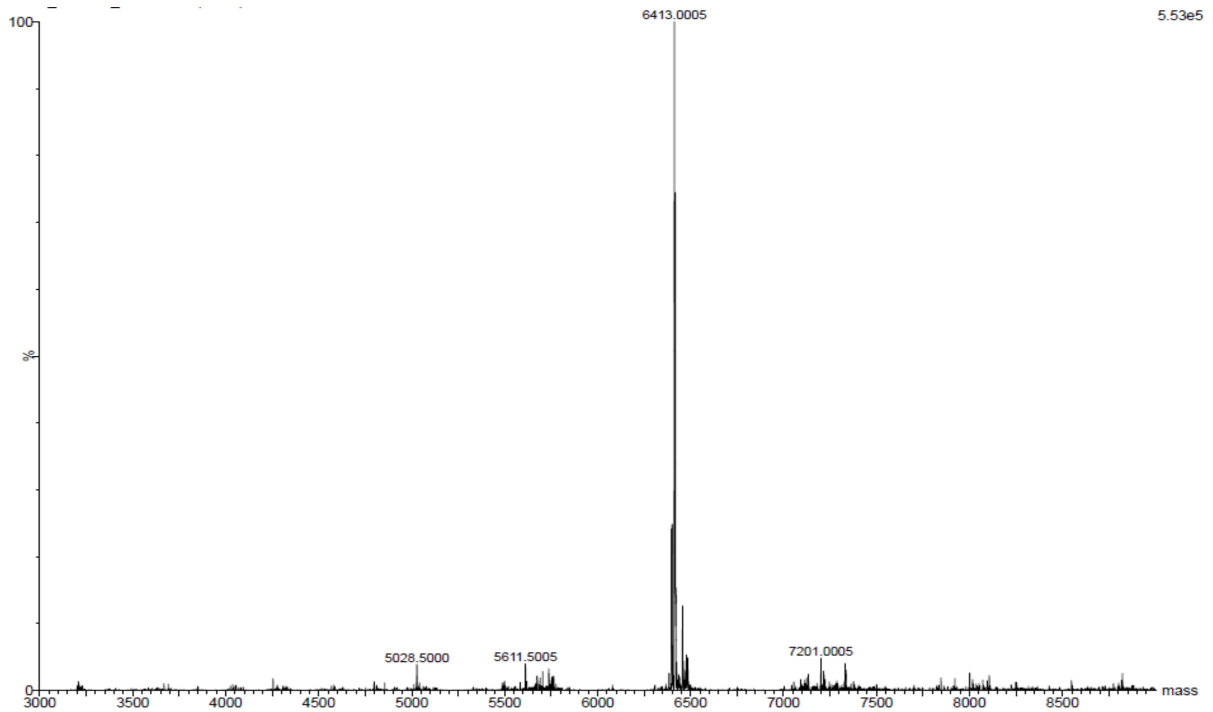

Supplementary Figure S16. Mass spectrum (ES-) of pure **DMT-ON ON1**. Required **6413.32** Da, found **6413.00** Da. y-axis = relative intensity (%), x-axis = mass in Da.

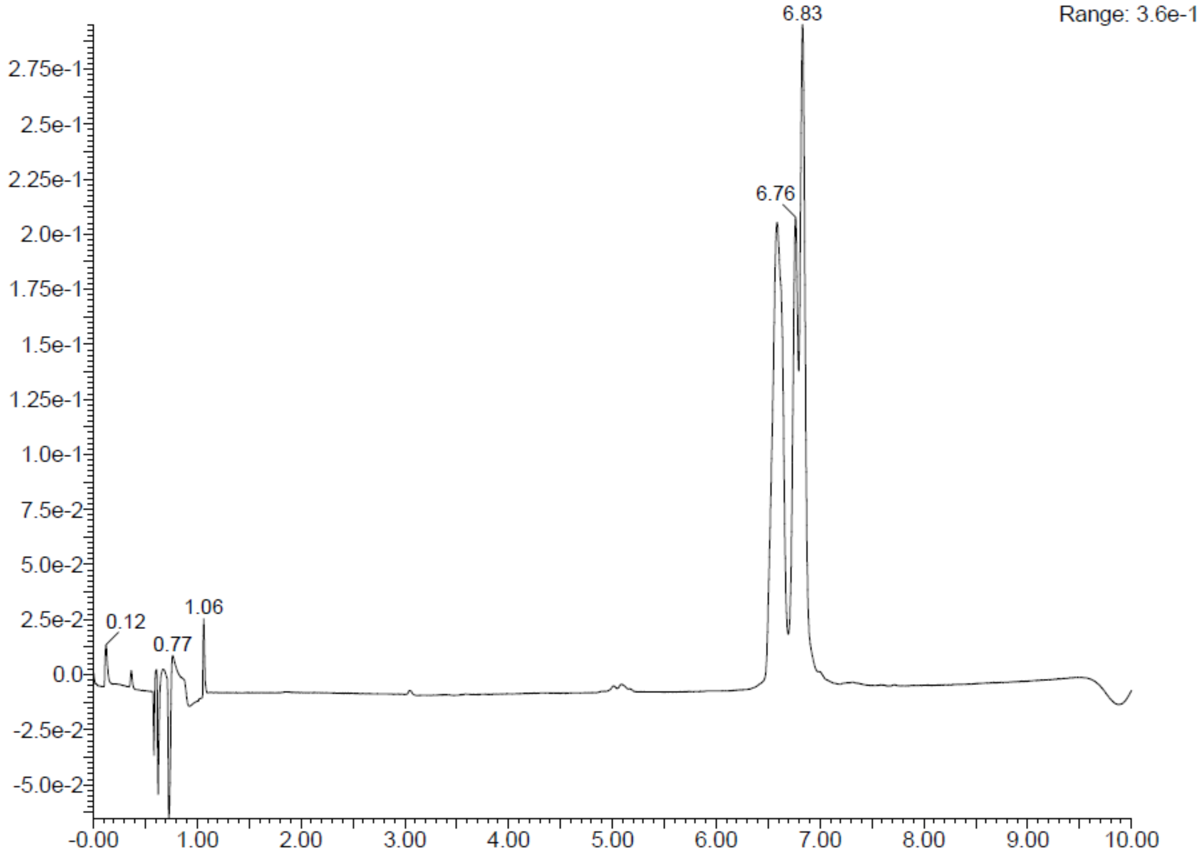

Supplementary Figure S17: Reverse-phase UPLC of **DMT-ON ON2** after deprotection from solid support **with EDA** followed by purification by HPLC (UV absorbance at 260 nm vs time in min)

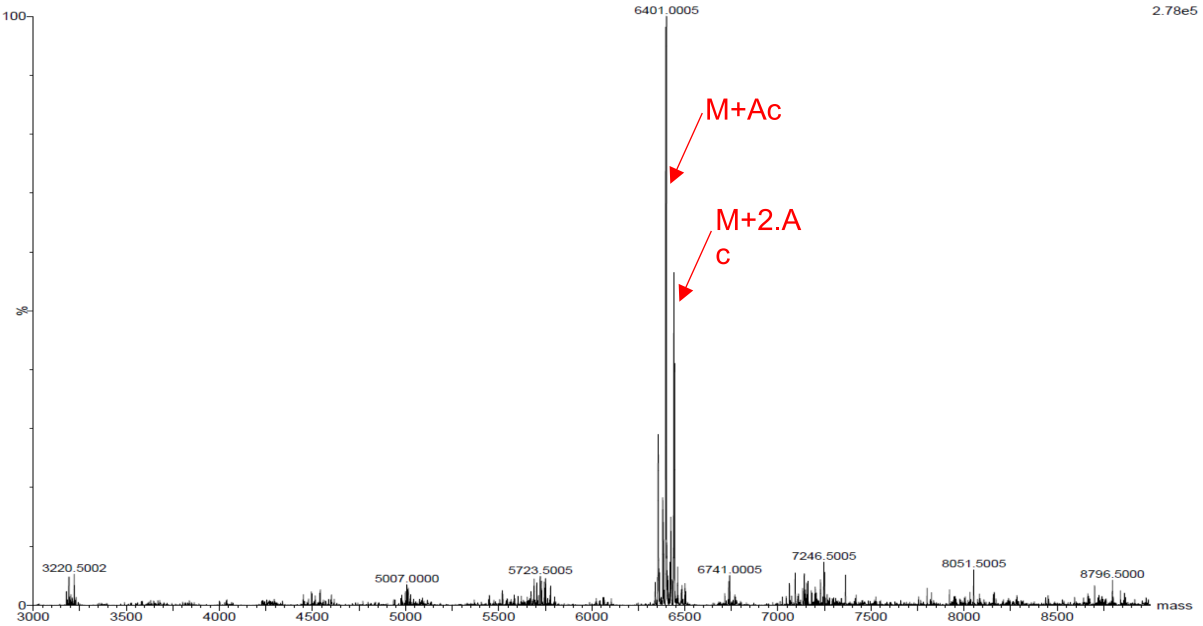

Supplementary Figure S18. Mass spectrum (ES-) of pure **DMT-ON ON2** after deprotection from solid support **with EDA** followed by purification by HPLC. Required 6355.34 Da, found **6401.00 and 6443.04** Da (presence of acetyl group). y-axis = relative intensity (%), x-axis = mass in Da.

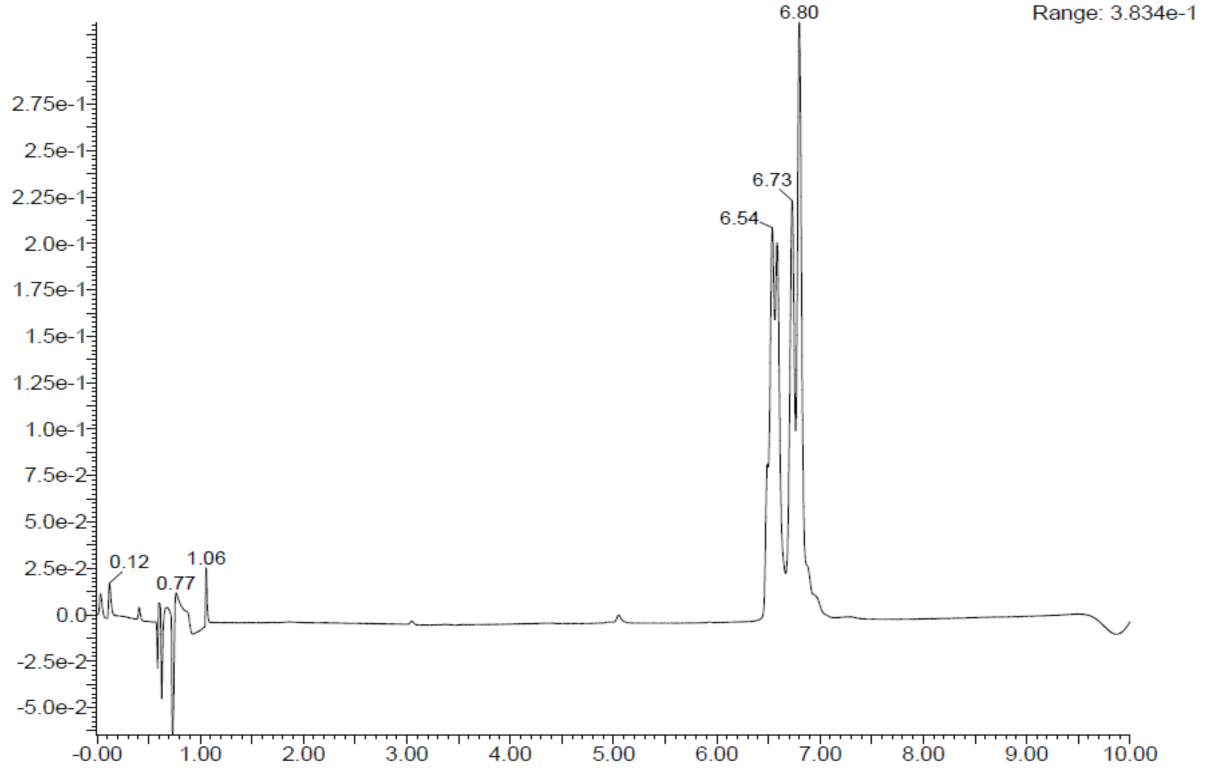

Supplementary Figure S19: Reverse-phase UPLC of **DMT-ON ON2** after deprotection from solid support **with ammonia** followed by purification by HPLC (UV absorbance at 260 nm vs time in min)

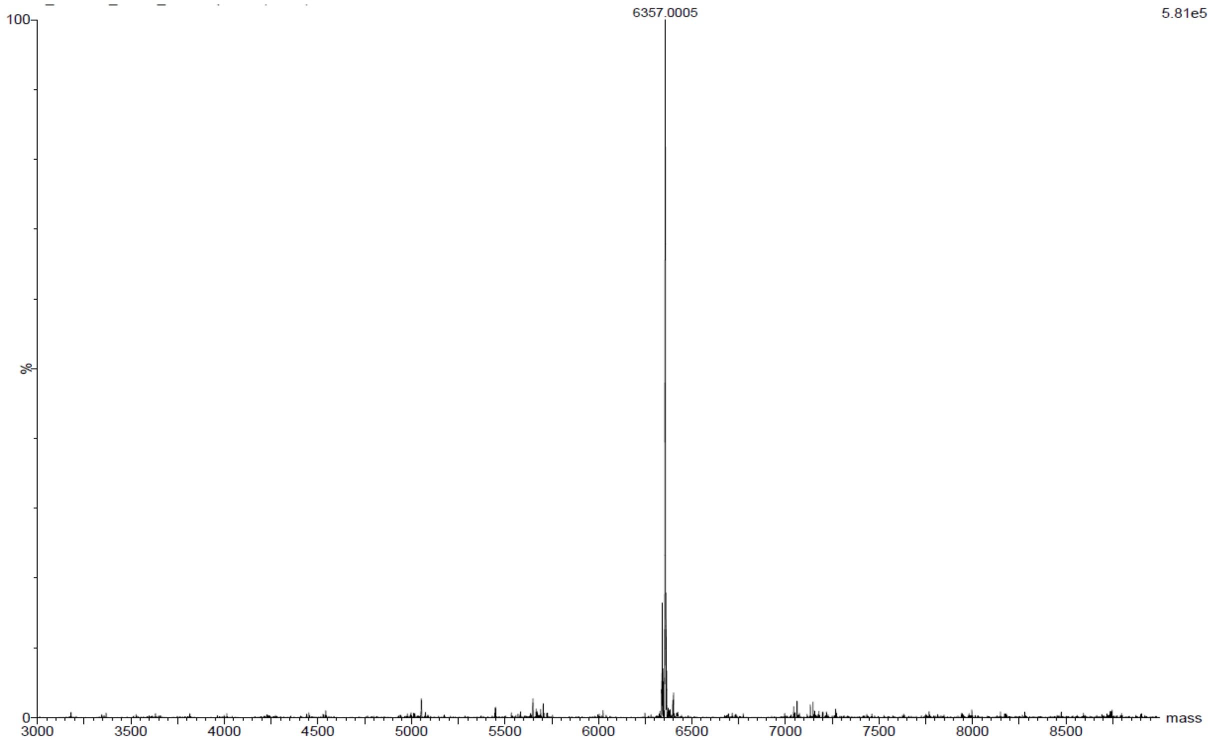

Supplementary Figure S20. Mass spectrum (ES-) of pure **DMT-ON ON2** after deprotection from solid support **with ammonia** followed by purification by HPLC. Required **6355.34** Da, found **6357.00** Da. y-axis = relative intensity (%), x-axis = mass in Da.

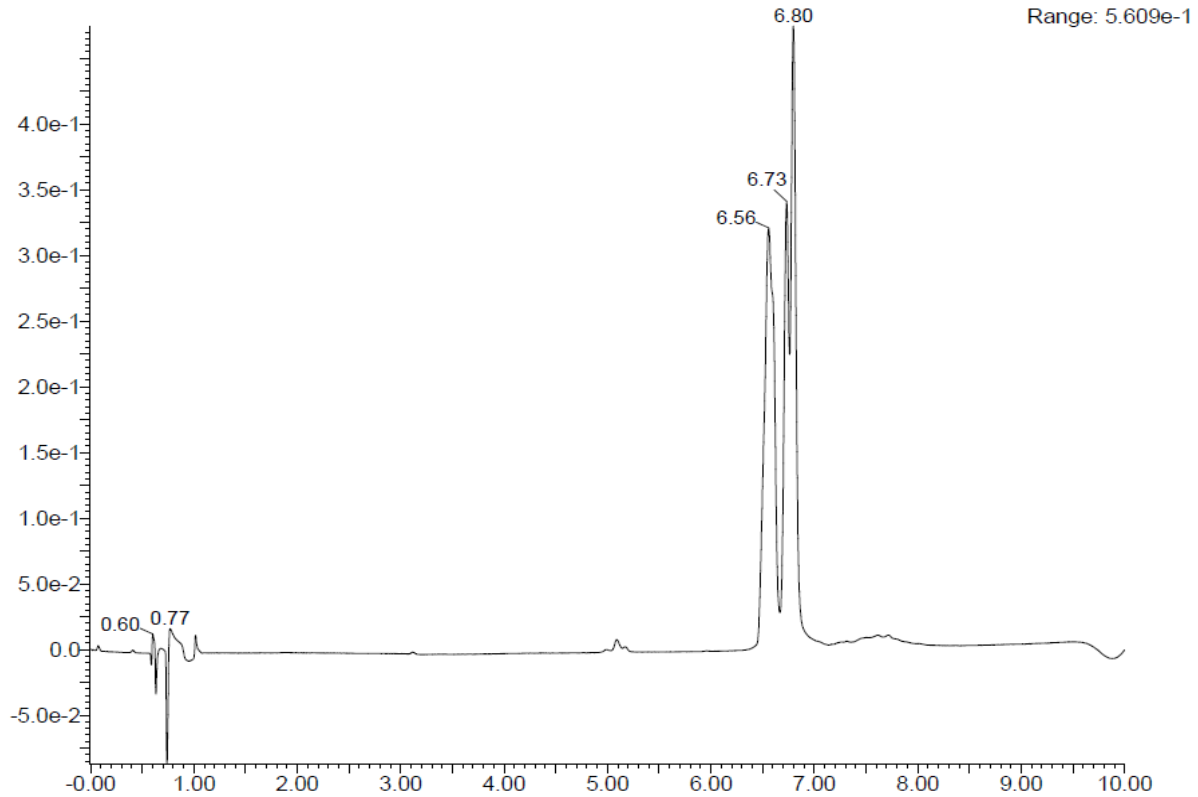

Supplementary Figure S21: Reverse-phase UPLC of **DMT-ON ON3** after deprotection from solid support **with EDA** followed by purification by HPLC (UV absorbance at 260 nm vs time in min)

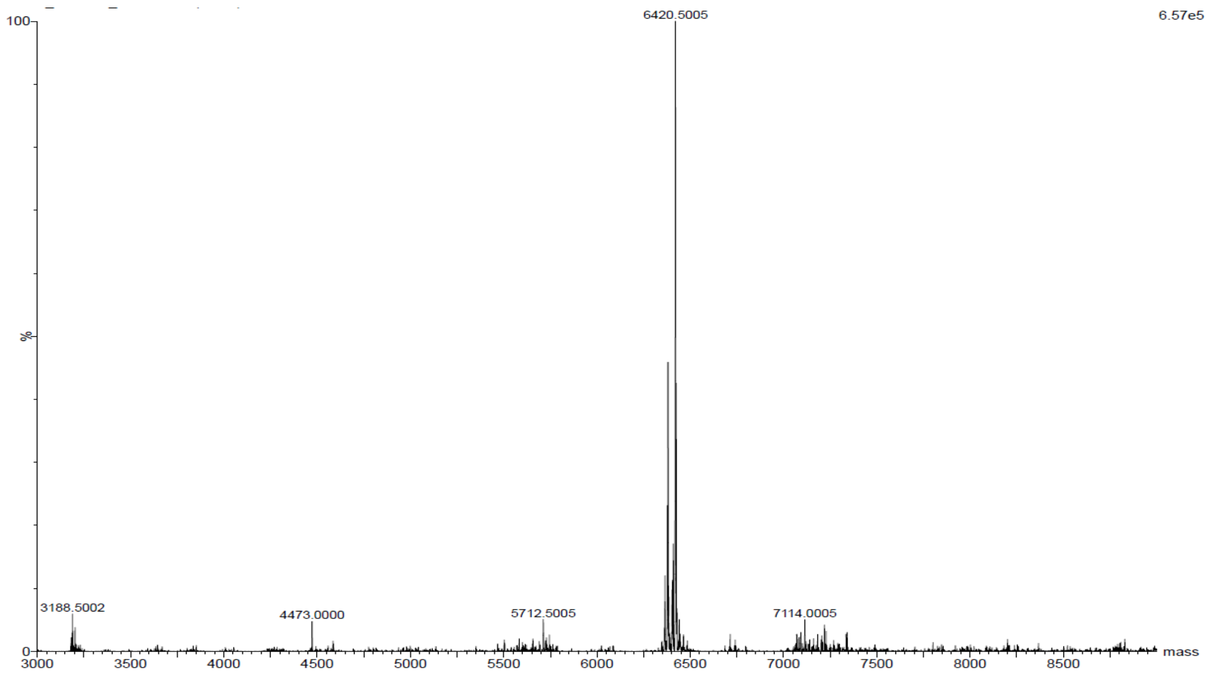

Supplementary Figure S22. Mass spectrum (ES-) of pure **DMT-ON ON3** after deprotection from solid support **with EDA** followed by purification by HPLC. Required **6376.91** Da, found **6420.50** Da (presence of acetyl group). y-axis = relative intensity (%), x-axis = mass in Da.

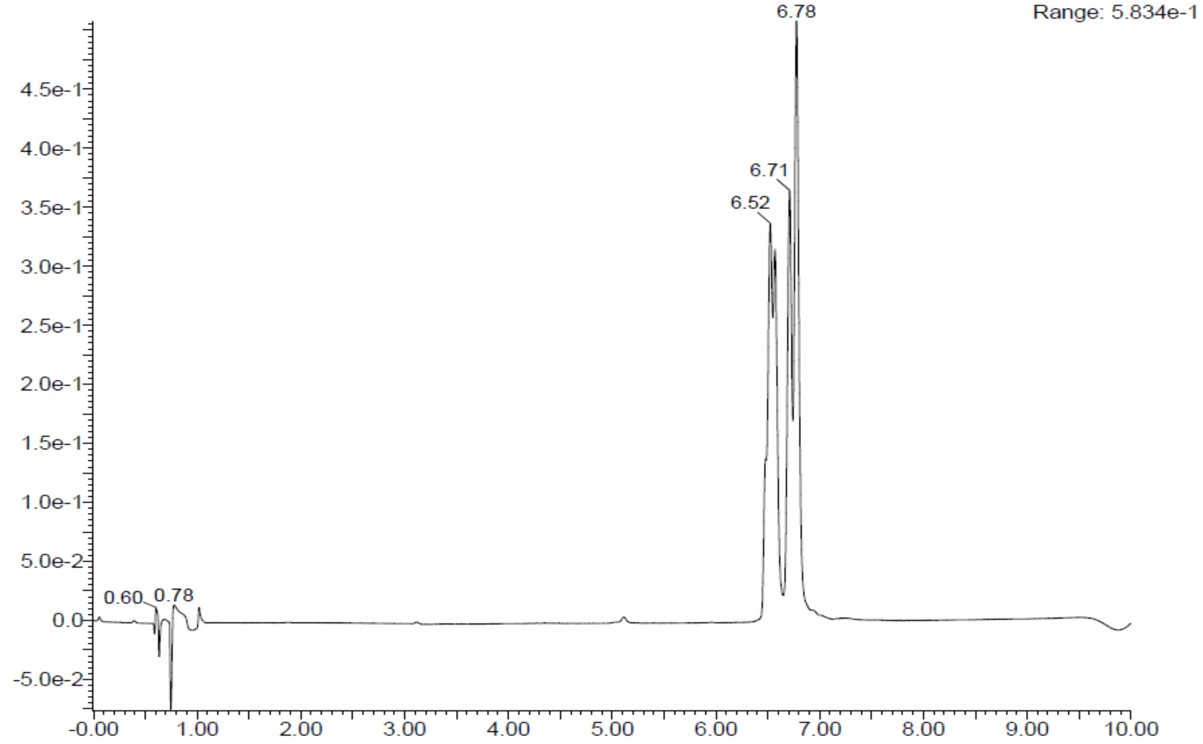

Supplementary Figure S23: Reverse-phase UPLC of **DMT-ON ON3** after deprotection from solid support **with ammonia** followed by purification by HPLC (UV absorbance at 260 nm vs time in min)

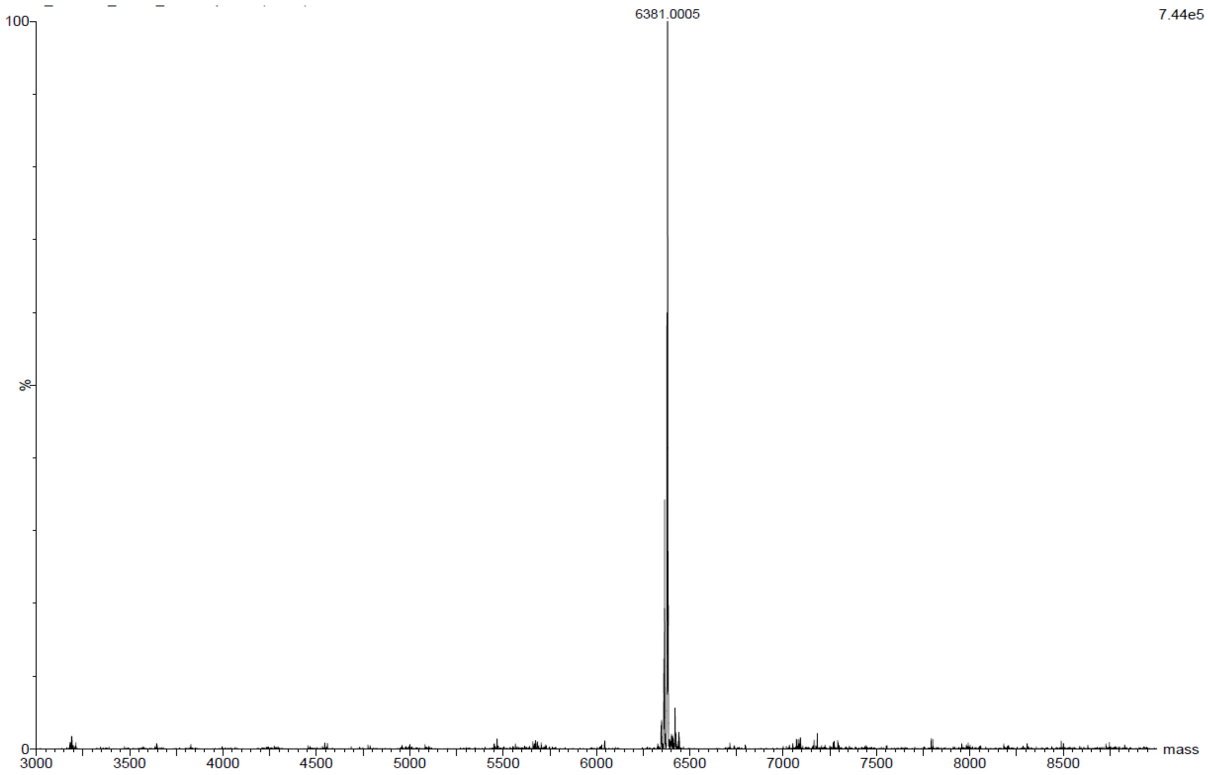

Supplementary Figure S24. Mass spectrum (ES-) of pure **DMT-ON ON3** after deprotection from solid support **with ammonia** followed by purification by HPLC. Required **6376.91** Da, found **6381.00** Da. y-axis = relative intensity (%), x-axis = mass in Da.

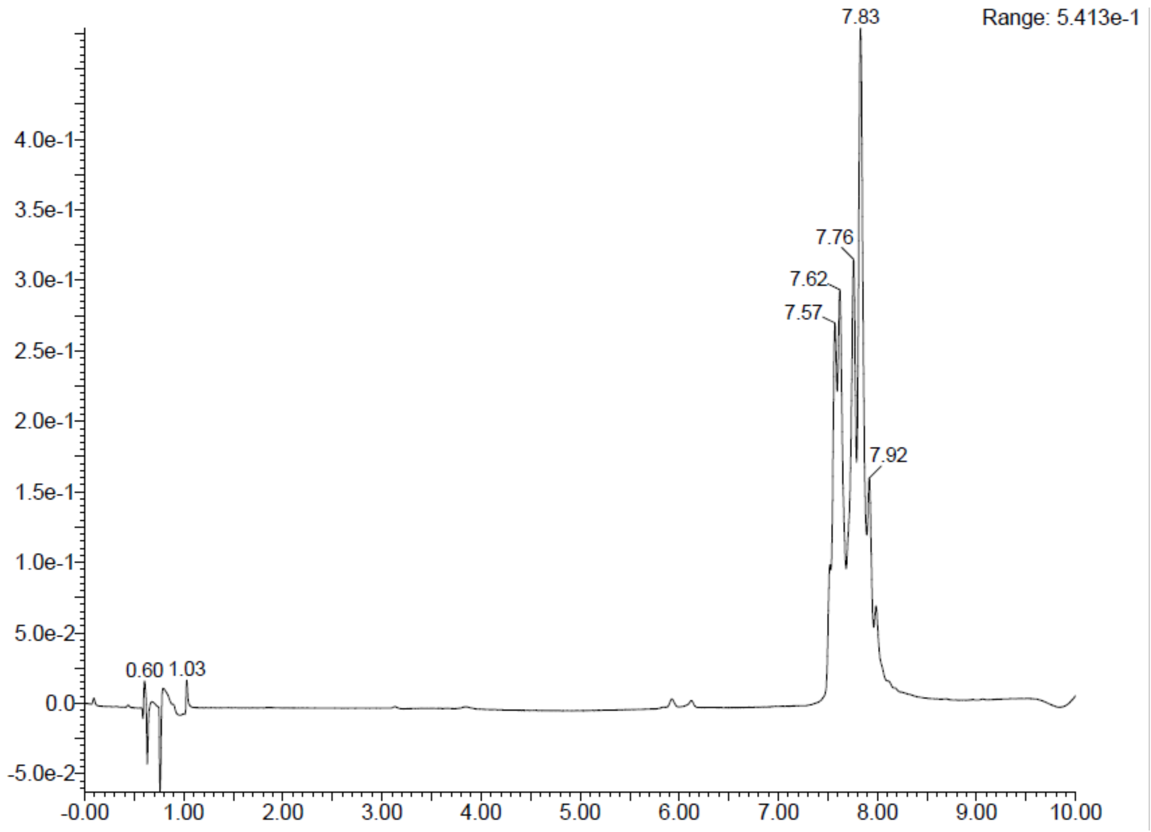

Supplementary Figure S25: Reverse-phase UPLC of **DMT-ON ON4** after deprotection from solid support **with ammonia** followed by purification by HPLC (UV absorbance at 260 nm vs time in min)

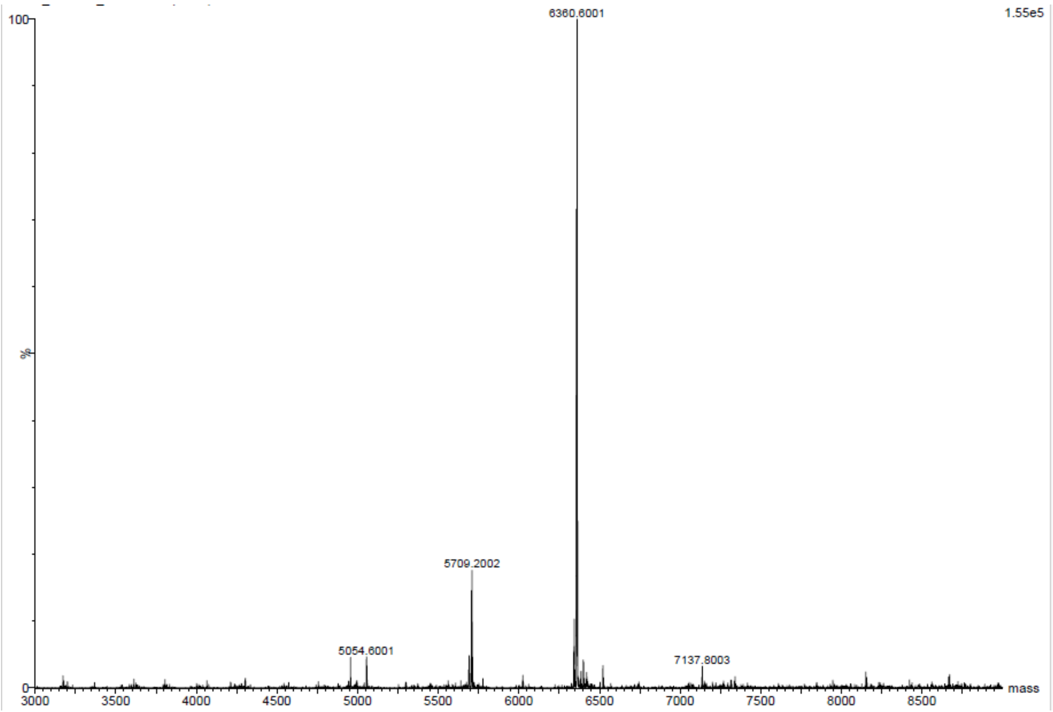

Supplementary Figure S26. Mass spectrum (ES-) of pure **DMT-ON ON4** after deprotection from solid support **with ammonia** followed by purification by HPLC. Required **6357.32** Da, found **6360.60** Da. y-axis = relative intensity (%), x-axis = mass in Da.

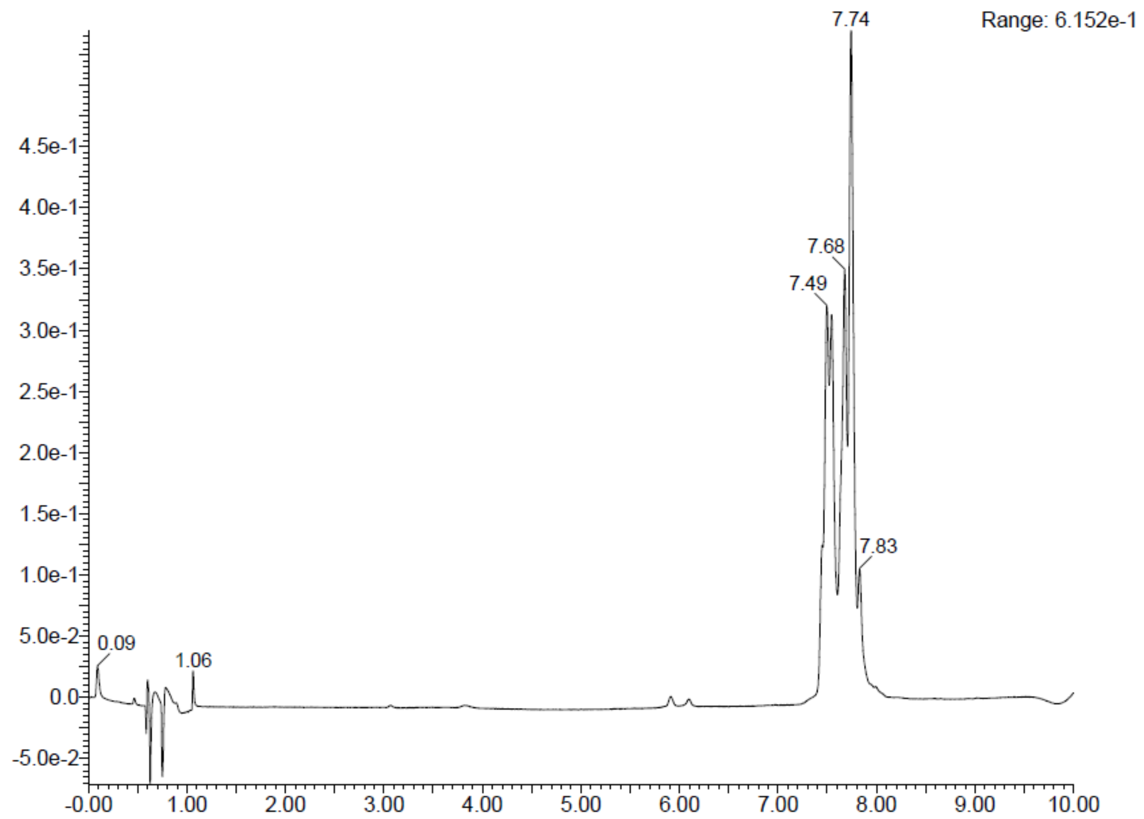

Supplementary Figure S27: Reverse-phase UPLC of **DMT-ON ON5** after deprotection from solid support **with ammonia** followed by purification by HPLC (UV absorbance at 260 nm vs time in min)

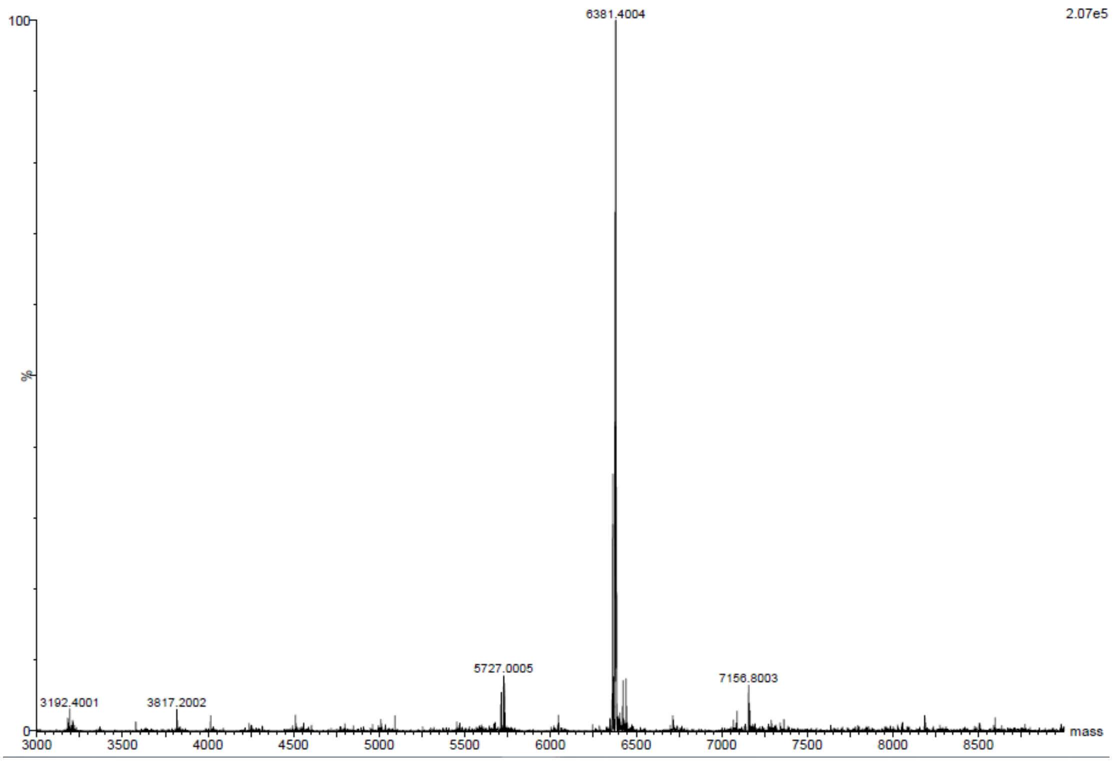

Supplementary Figure S28. Mass spectrum (ES-) of pure **DMT-ON ON5** after deprotection from solid support **with ammonia** followed by purification by HPLC. Required **6377.90** Da, found **6381.40** Da. y-axis = relative intensity (%), x-axis = mass in Da.

6. UV melting experiments

UV melting experiments were performed using a Cary 4000 scan UV-vis spectrophotometer. 2 nmol of each oligonucleotide was dissolved in 1 mL of 10 mM phosphate buffer containing 100 mM NaCl at pH 7.0. The samples were first denatured by heating to 85 °C (10 °C/min) and then annealed by slowly cooling to 20 °C (1 °C/min). Six successive cycles of heating and cooling were performed (20 °C to 85 °C) at a gradient of 1 °C/min whilst recording the change in UV absorbance at 260 nm. Cary WinUV 3.0 software was then used to calculate the melting temperature from the first derivative of the averaged melting curves.

.

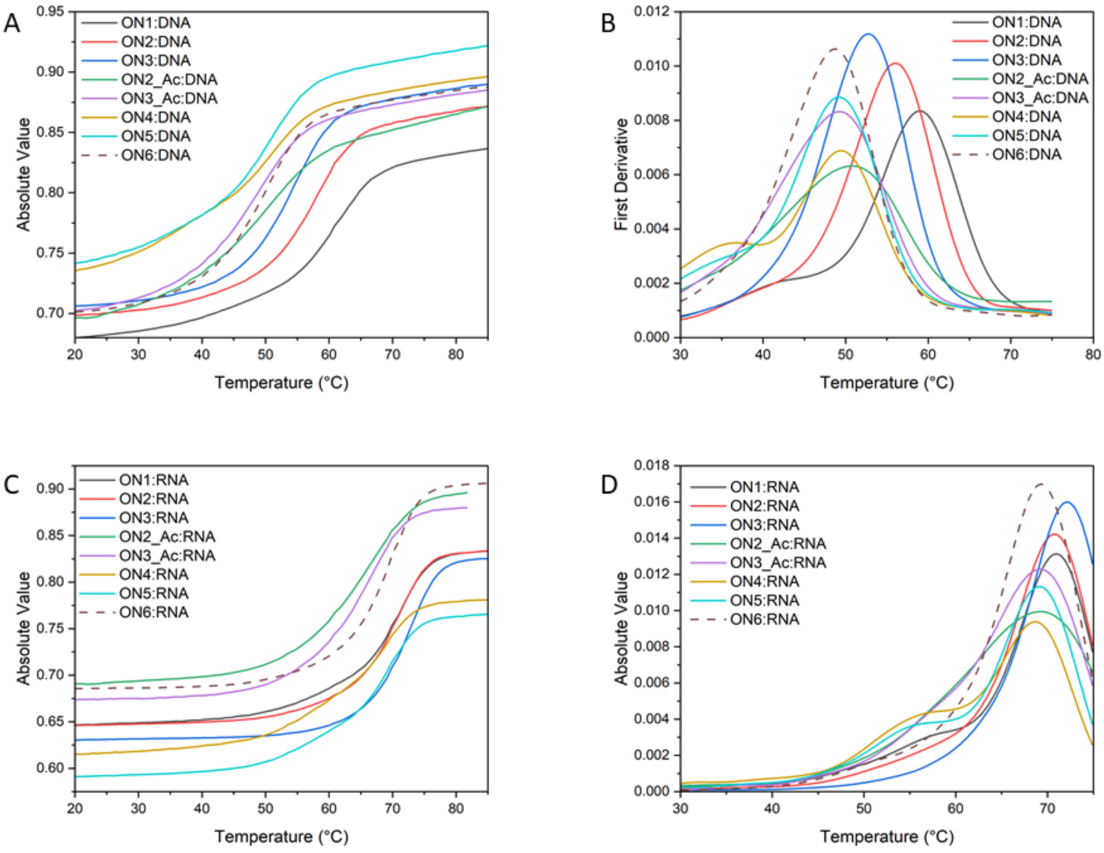

Supplementary Figure S29: UV melting studies for oligonucleotides with complementary DNA and RNA. A) Representative melting curves for DNA using 2 μM of each oligonucleotide in 100 mM NaCl, 10 mM phosphate buffer at pH 7.0; B) 1st derivative of melting curves for DNA; C) Representative melting curves for RNA; D) 1st derivative of melting curves for RNA.

7. Circular Dichroism

CD spectra were acquired with a Chirascan CD spectrometer (Applied Photophysics Ltd). Duplex sequences (2 μM, 10 mM sodium phosphate buffer, pH 7.0, 100 mM NaCl) were measured in 2 mm quartz cuvettes at 25 °C. Spectra were obtained by the accumulation of six scans over the range of 220–330 nm.

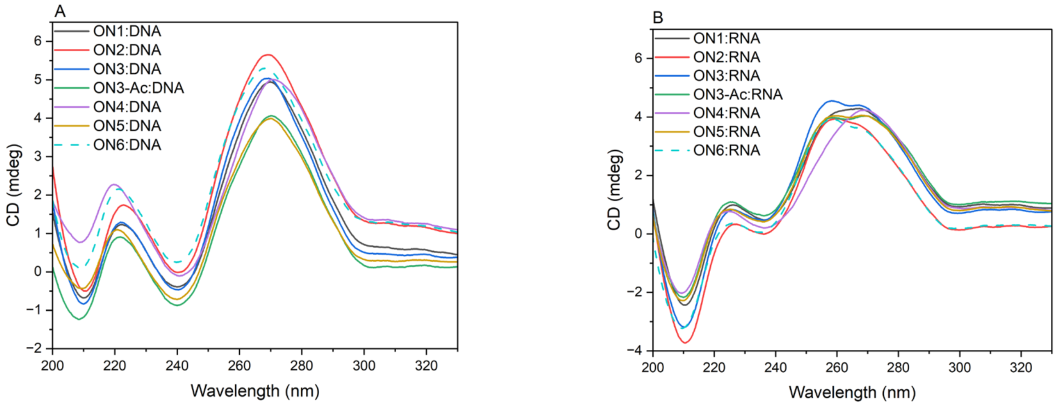

Supplementary Figure S30. CD spectra of modified oligonucleotides against (2 μM, 10 mM Sodium phosphate buffer, pH 7.0, 100 mM NaCl) A) DNA targets and B) RNA targets.

8. Nuclease stability

Nuclease S1 from Aspergillus oryzae and Gibco PBS buffer (pH 7.0) were mixed and the control DNA (unmodified PO backbone) or modified oligonucleotide (**ON1-ON5**) was added to reach final concentration 5 µM. The sample was mixed by pipetting and 10 µL of this solution was immediately removed, mixed with formamide (10 µL) and stored at −20 °C (0 h). The remaining reaction mixtures were incubated at 37 °C and aliquots (10 µL) were taken at different time intervals, mixed with formamide (10 µL), and stored at −20 °C. 10 µL of each sample was taken, 10 µl loading buffer was added and the protein denatured at 95 °C for 2 minutes. The samples were then analysed by denaturing 20% polyacrylamide gel electrophoresis.

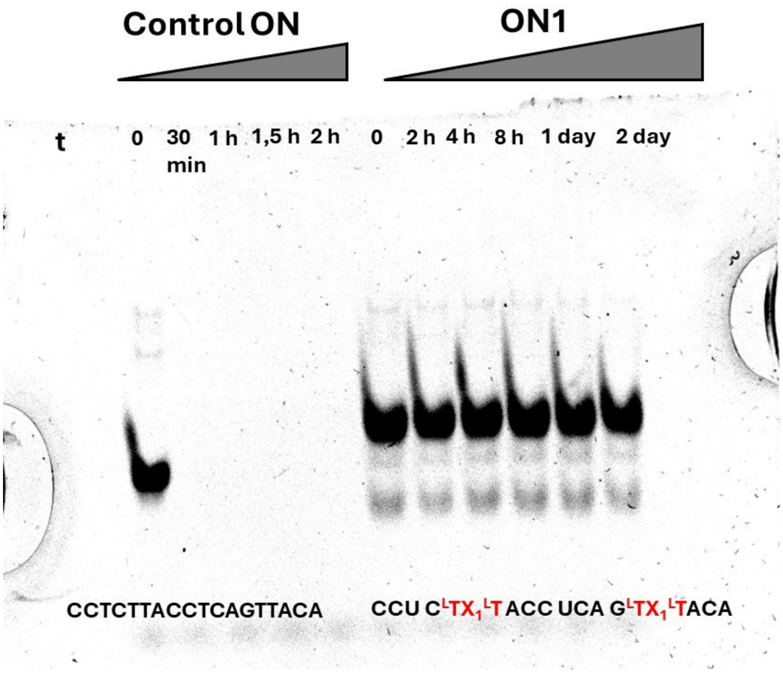

Supplementary Figure 31. Denaturing polyacrylamide gel electrophoresis (PAGE) analysis of modified **ON1** and unmodified **control ON** after incubation in Nuclease S1 *Aspergillus oryzae*:PBS (50 µL:50 µL). t = incubation time.

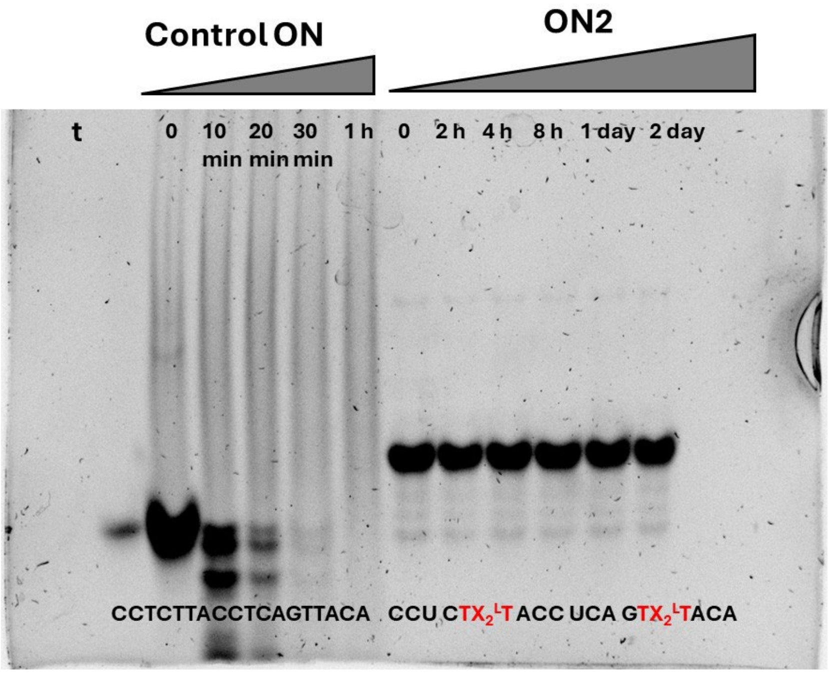

Supplementary Figure 32. Denaturing polyacrylamide gel electrophoresis (PAGE) analysis of modified **ON2** and unmodified **control ON** after incubation in Nuclease S1 *Aspergillus oryzae*:PBS (10 µL:85 µL). t = incubation time.

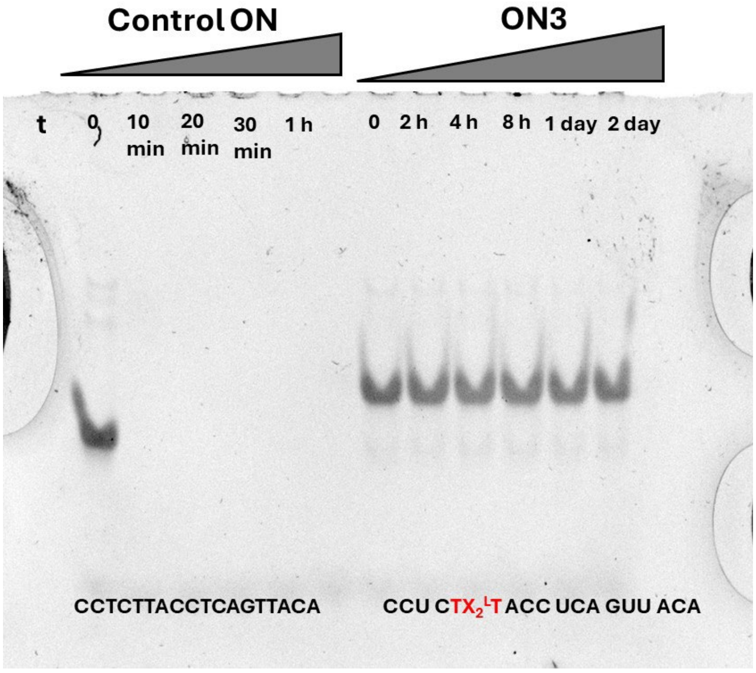

Supplementary Figure 33. Denaturing polyacrylamide gel electrophoresis (PAGE) analysis of modified **ON3** and unmodified **control ON** after incubation in Nuclease S1 *Aspergillus oryzae*:PBS (50 µL:50 µL). t = incubation time.

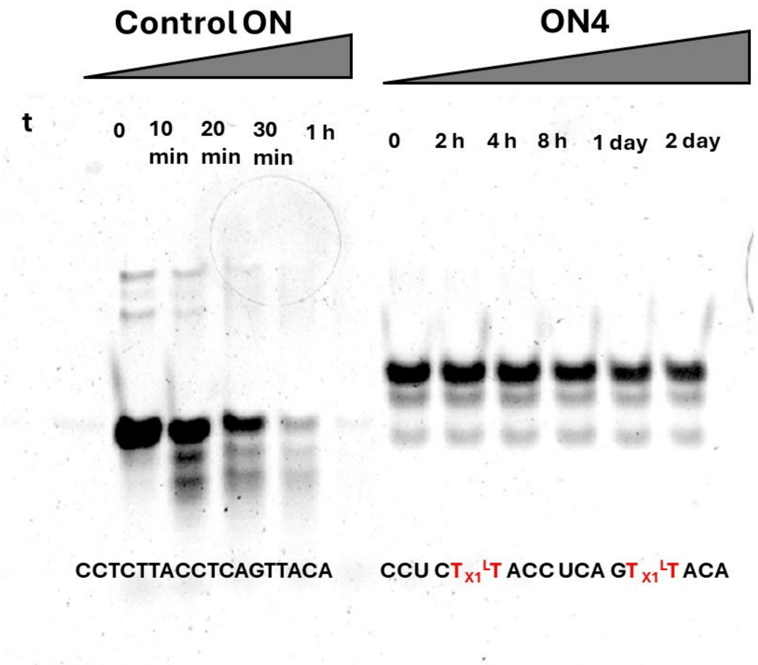

Supplementary Figure 34. Denaturing polyacrylamide gel electrophoresis (PAGE) analysis of modified **ON4** and unmodified **control ON** after incubation in Nuclease S1 *Aspergillus oryzae*:PBS (10 µL:85 µL). t = incubation time.

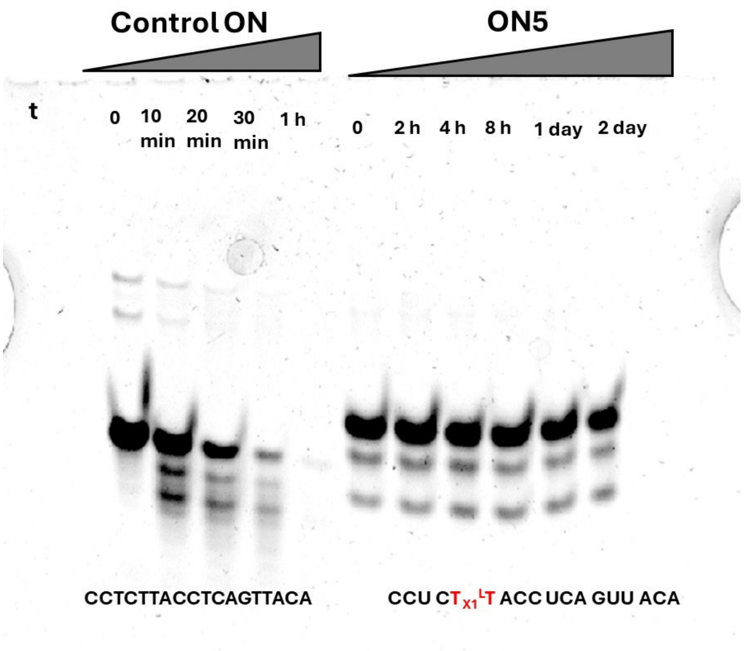

Supplementary Figure 35. Denaturing polyacrylamide gel electrophoresis (PAGE) analysis of modified **ON5** and unmodified **control ON** after incubation in Nuclease S1 *Aspergillus oryzae*:PBS (10 µL:85 µL). t = incubation time.

9. References

(1) Koshkin, A. A.; Fensholdt, J.; Pfundheller, H. M.; Lomholt, C. A simplified and efficient route to 2'-O, 4'-C-methylene-linked bicyclic ribonucleosides (locked nucleic acid). J Org Chem 2001, 66 (25), 8504-8512. DOI: 10.1021/jo010732p.

(2) Nielsen, P.; Christensen, N. K.; Dalskov, J. K. α-LNA (locked nucleic acid with α-D-configuration): Synthesis and selective parallel recognition of RNA. Chem-Eur J 2002, 8 (3), 712-722. DOI: 10.1002/1521-3765(20020201)8:3<712::Aid-Chem712>3.0.Co;2-0.

(3) Palframan, M. J.; Alharthy, R. D.; Powalowska, P. K.; Hayes, C. J. Synthesis of triazole-linked morpholino oligonucleotides via Cu(I) catalysed cycloaddition. Org Biomol Chem 2016, 14 (11), 3112-3119. DOI: 10.1039/c6ob00007j.

(4) Lin, L.; Sheng, J.; Momin, R.; Du, Q.; Huang, Z. Facile Synthesis and Anti-Tumor Cell Activity of Se-Containing Nucleosides. Nucleos Nucleot Nucl 2009, 28 (1), 56-66. DOI: 10.1080/15257770802581765.

(5) Chen, J. K.; Schultz, R. G.; Lloyd, D. H.; Gryaznov, S. M. Synthesis of Oligodeoxyribonucleotide N3'-P5' Phosphoramidates. Nucleic Acids Res 1995, 23 (14), 2661-2668. DOI: 10.1093/nar/23.14.2661.

10. NMR Spectra of Compounds

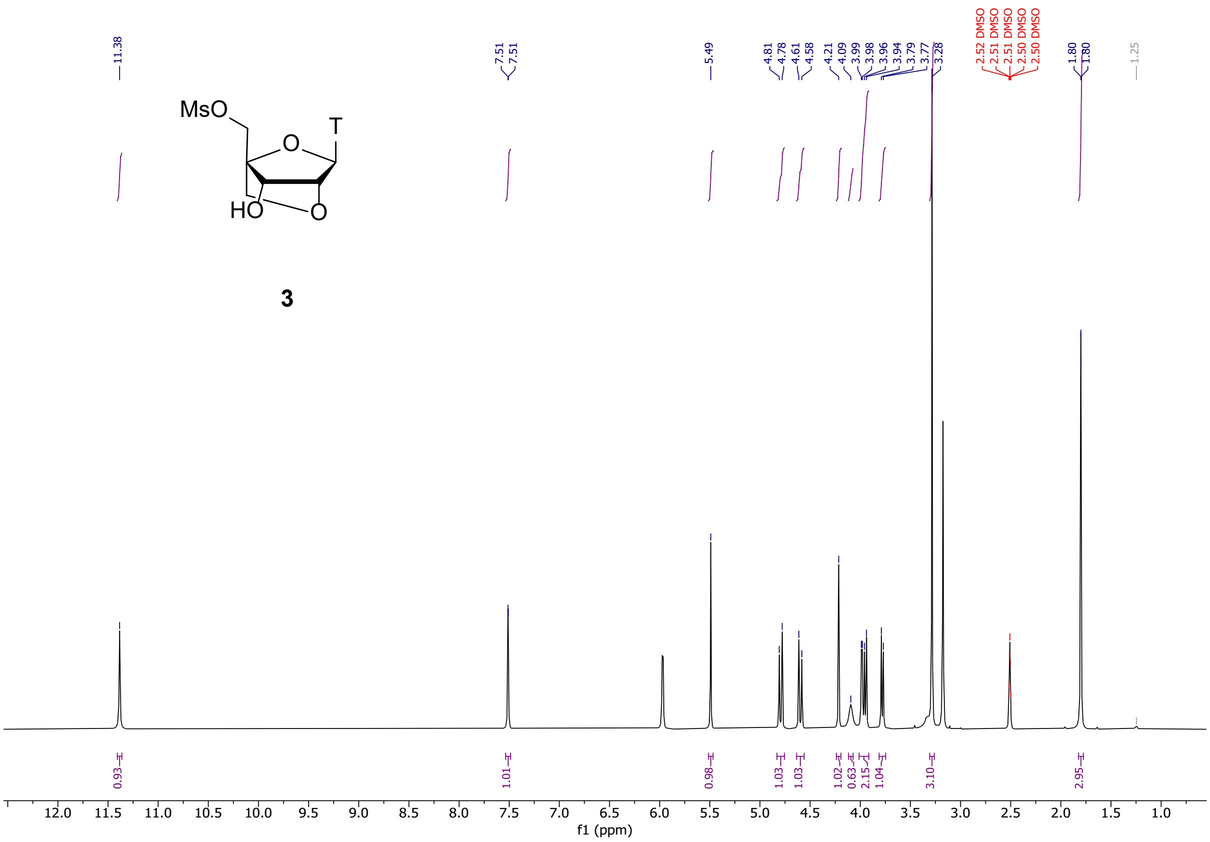

Supplementary Figure 36. ^{1}H NMR (400 MHz, DMSO-*d6*) spectrum of compound **3.**

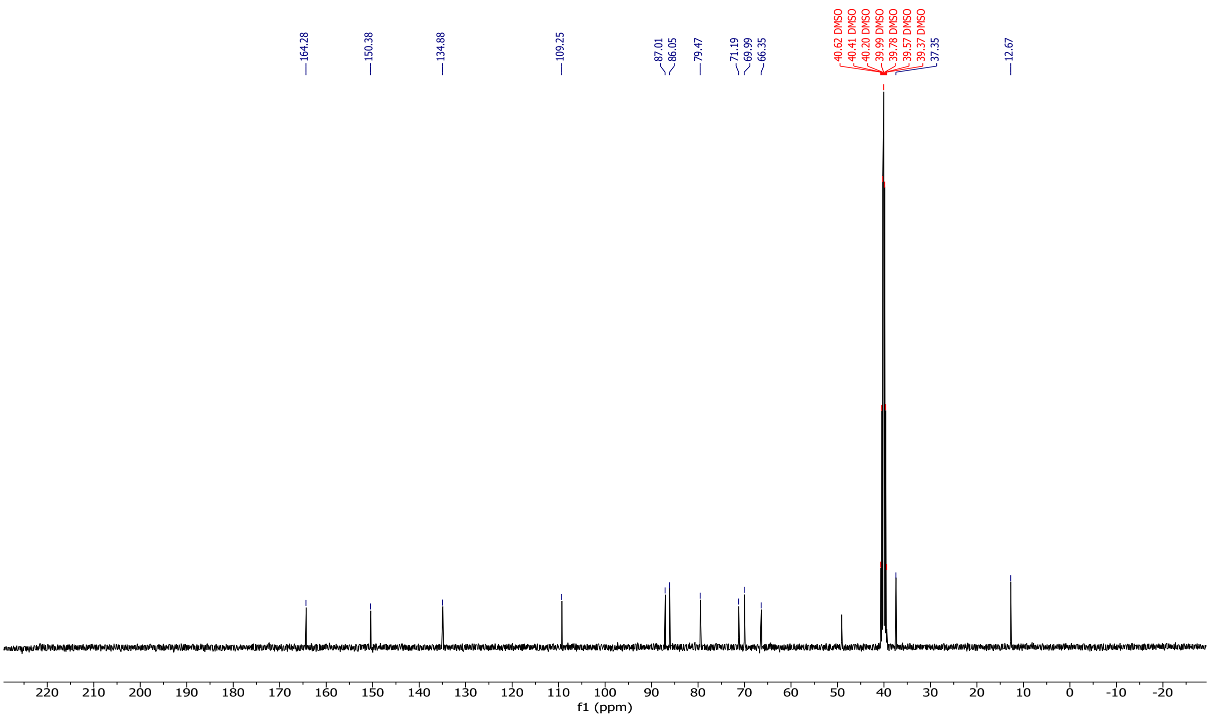

Supplementary Figure 37. ^{13}C NMR (101 MHz, DMSO-*d6*) spectrum of compound **3.**

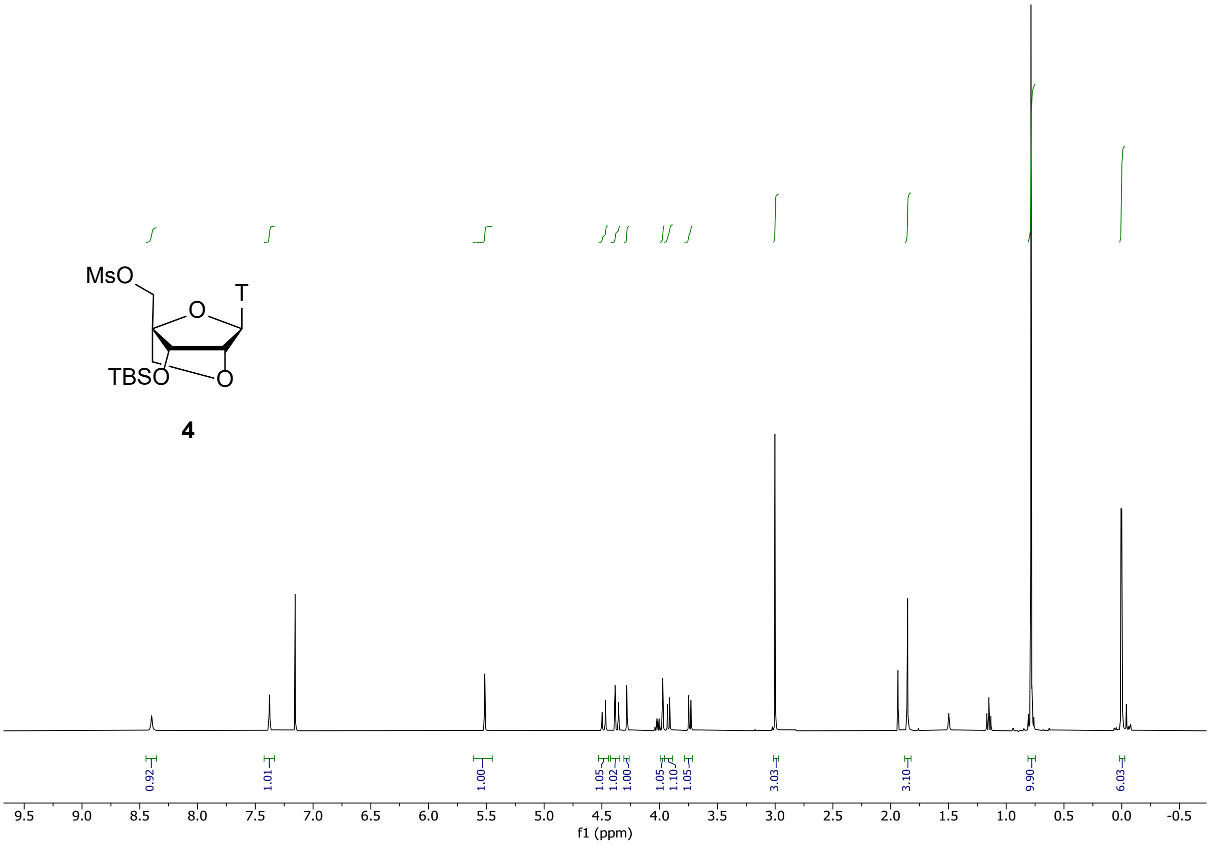

Supplementary Figure 38. ^{1}H NMR (400 MHz, $CDCl_3$) spectrum of compound **4**

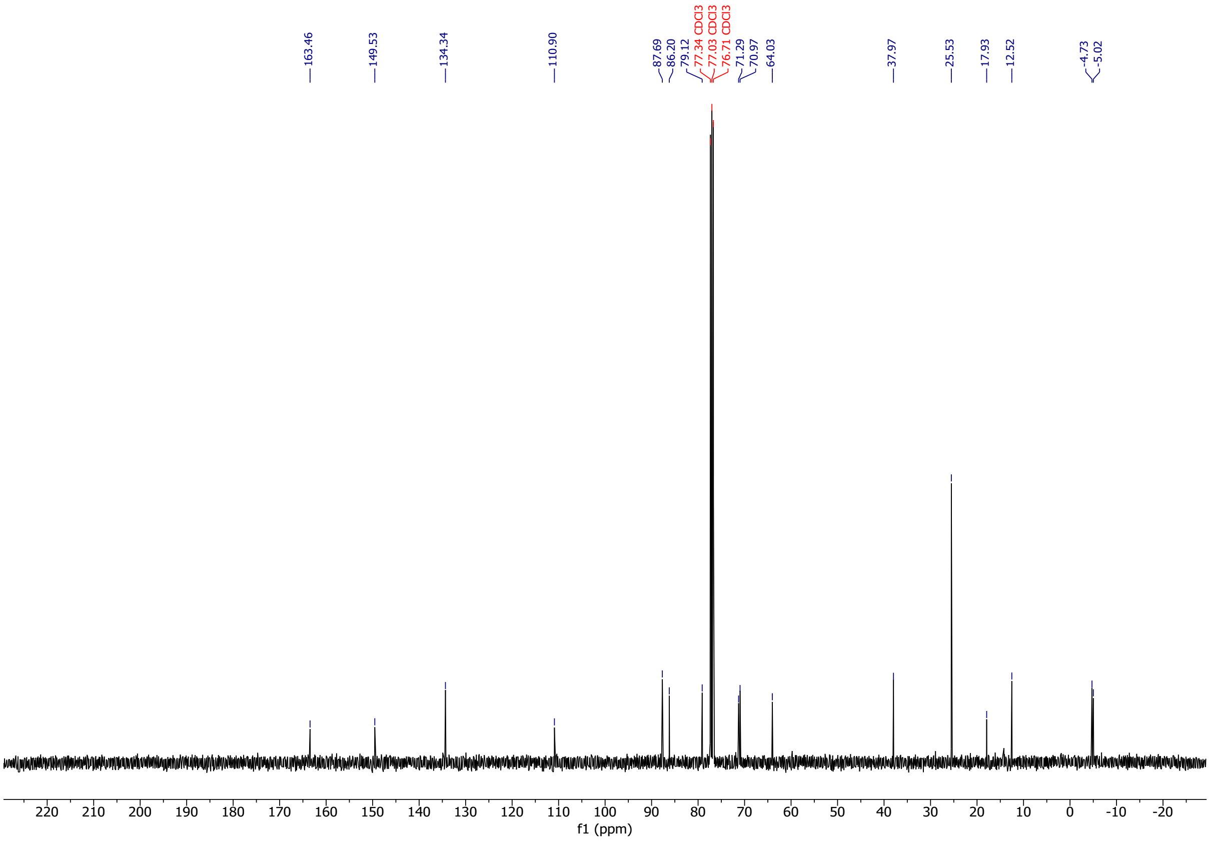

Supplementary Figure 39. ^{13}C NMR (101 MHz, $CDCl_3$) spectrum of compound **4.**

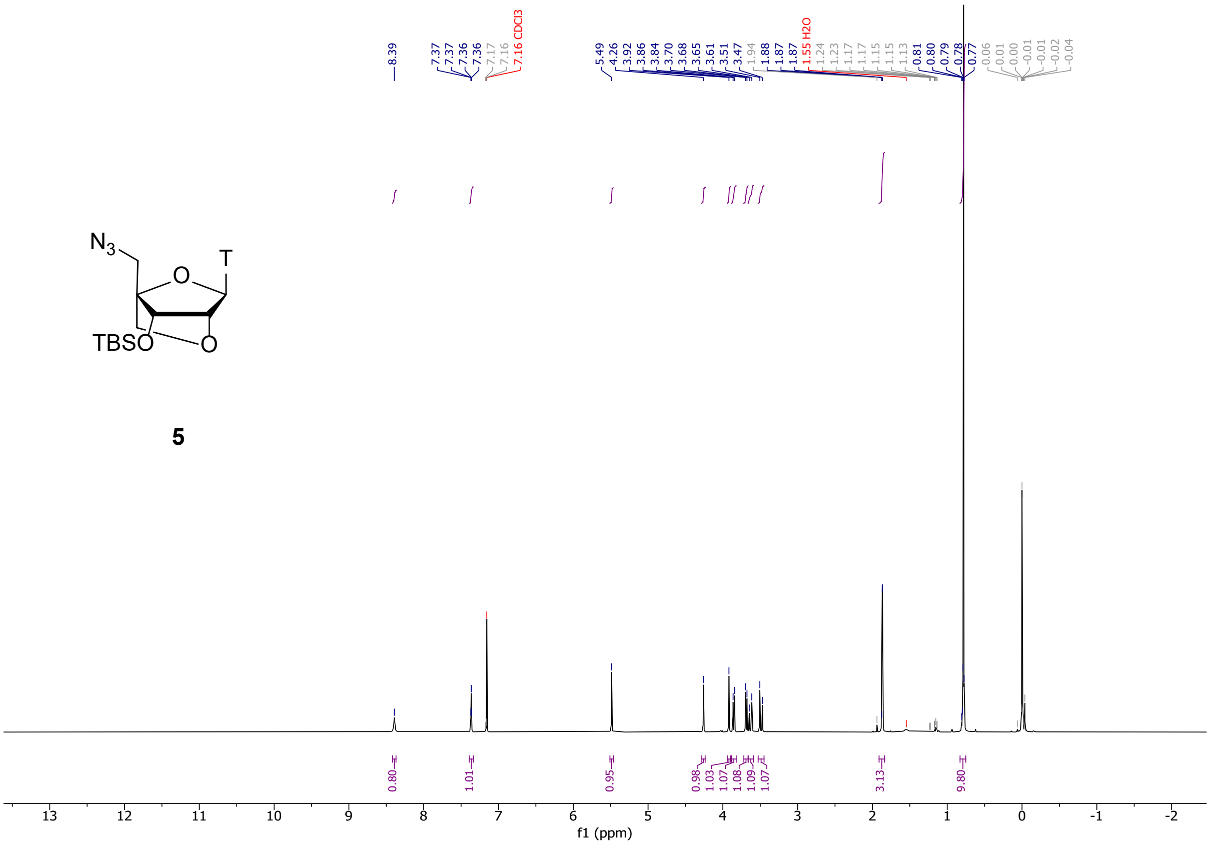

5

Supplementary Figure 40. ^{1}H NMR (400 MHz, $CDCl_3$) spectrum of compound **5.**

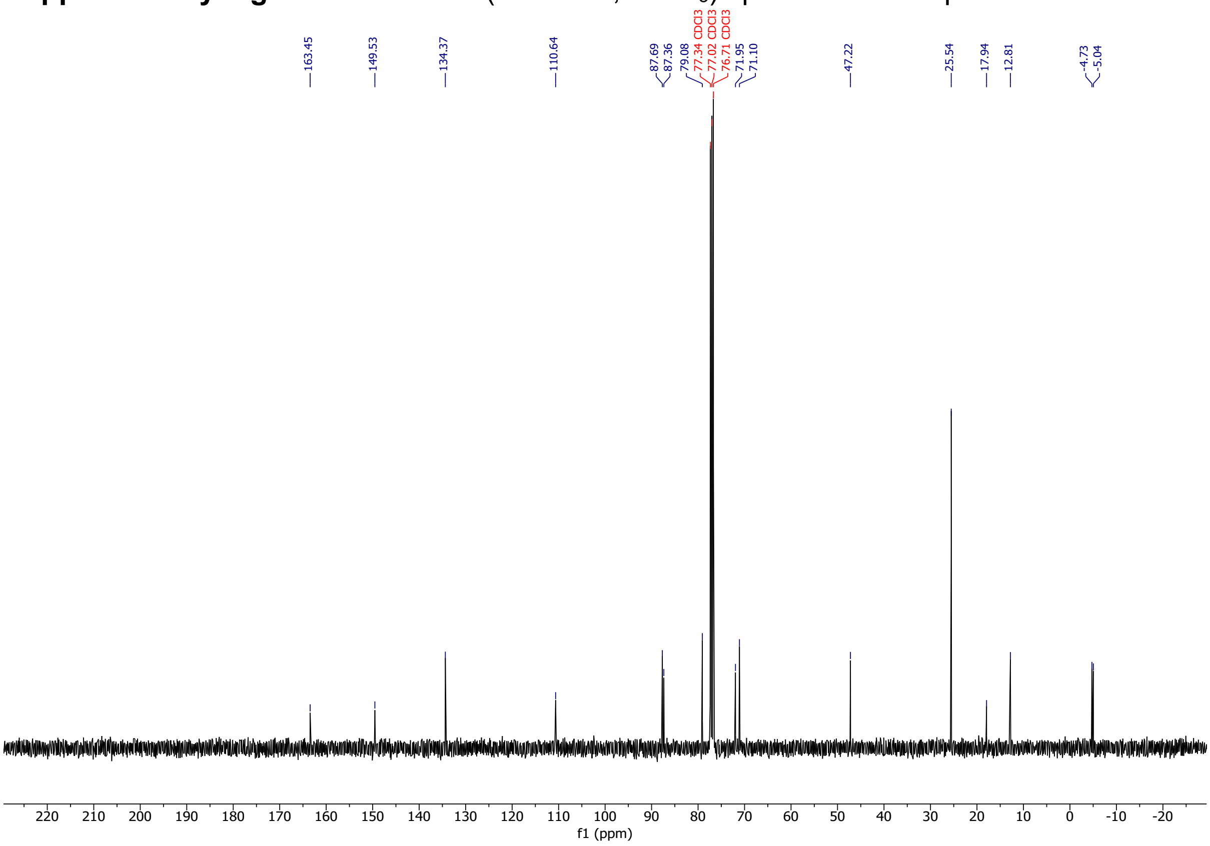

Supplementary Figure 41. ^{13}C NMR (101 MHz, $CDCl_3$) spectrum of compound **5.**

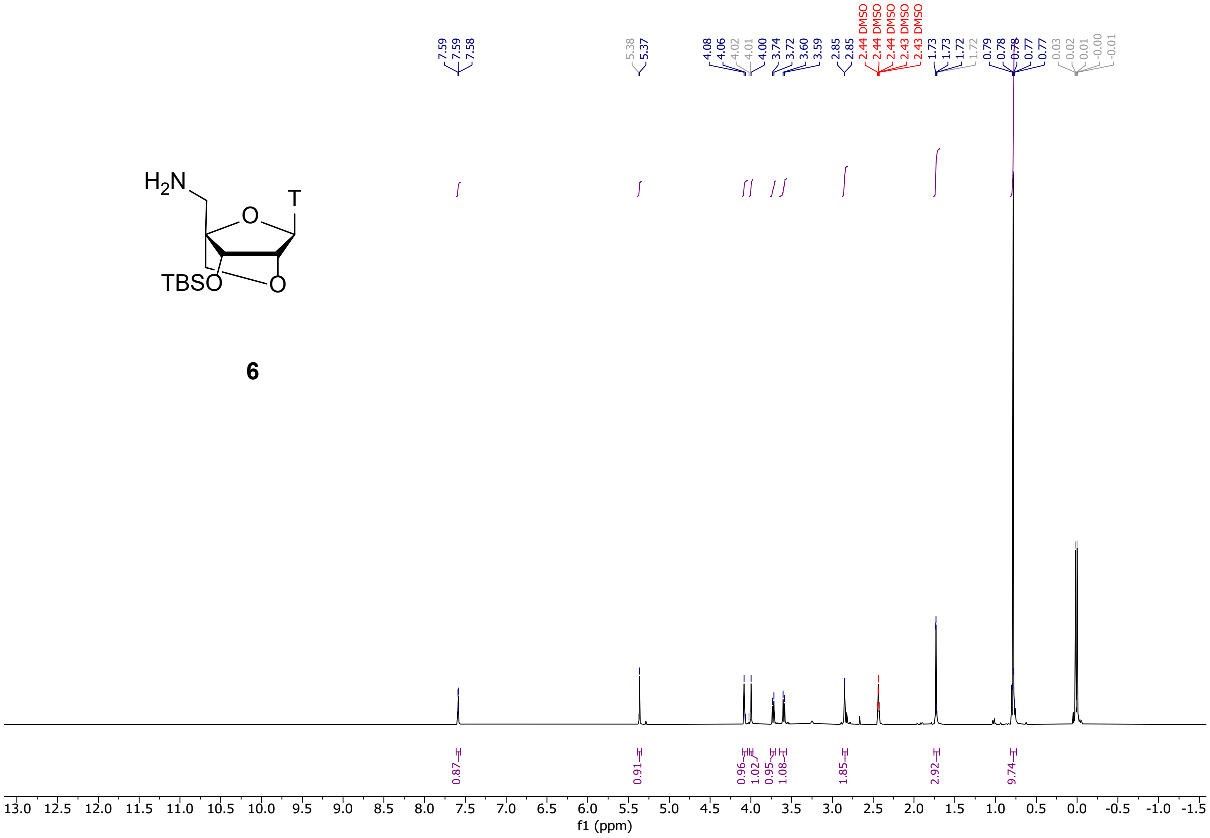

Supplementary Figure 42. ^{1}H NMR (400 MHz, DMSO-*d6*) spectrum of compound **6.**

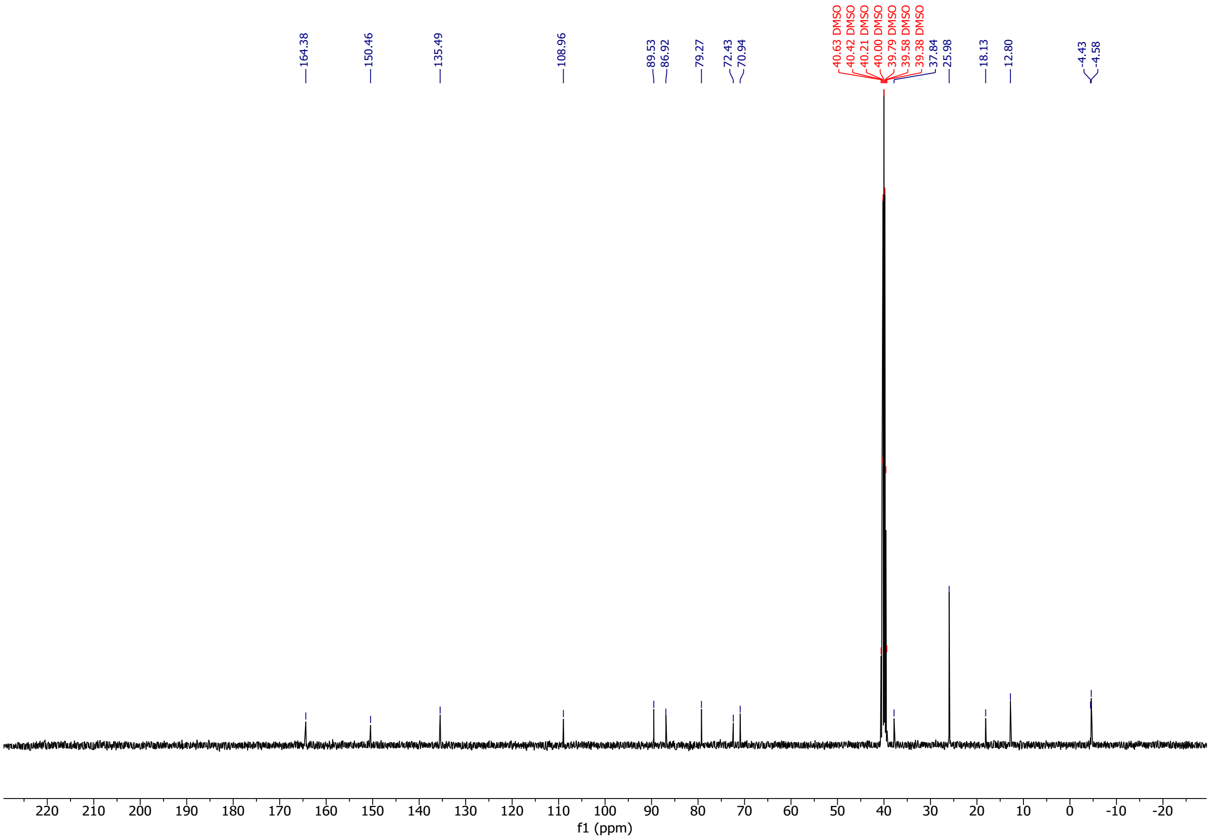

Supplementary Figure 43. ^{13}C NMR (101 MHz, DMSO-*d6*) spectrum of compound **6.**

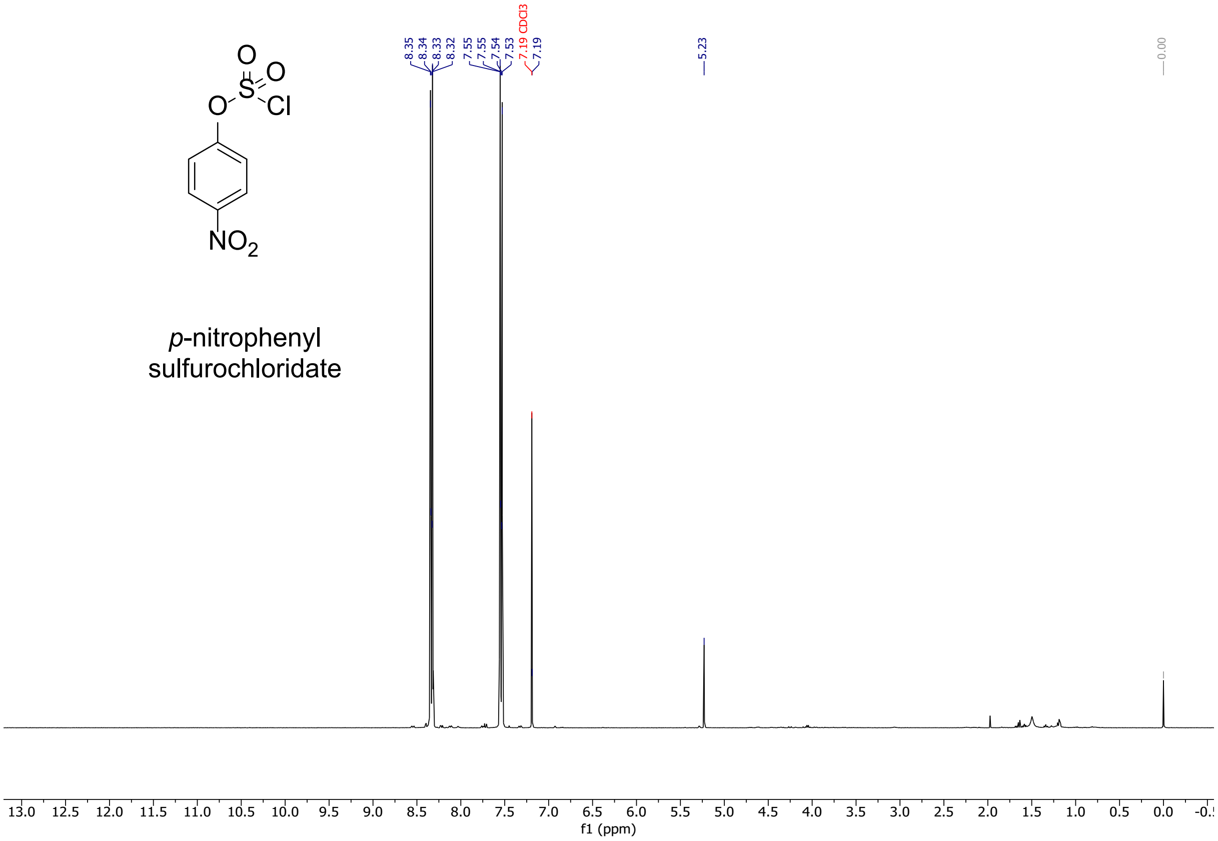

Supplementary Figure 44. ^{1}H NMR (400 MHz, $CDCl_3$) spectrum of 4-nitrophenyl sulfurochloridate**.**

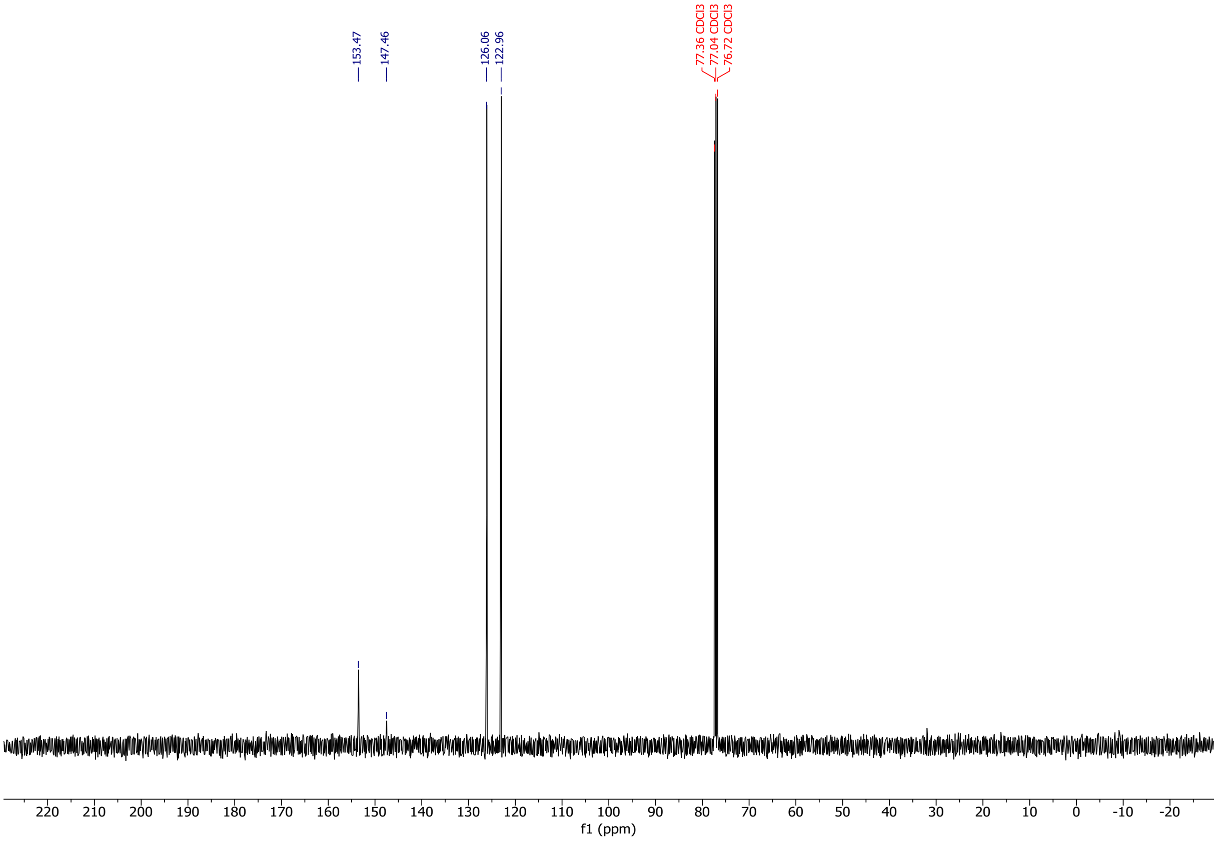

Supplementary Figure 45. ^{13}C NMR (101 MHz, $CDCl_3$) spectrum of 4-nitrophenyl sulfurochloridate**.**

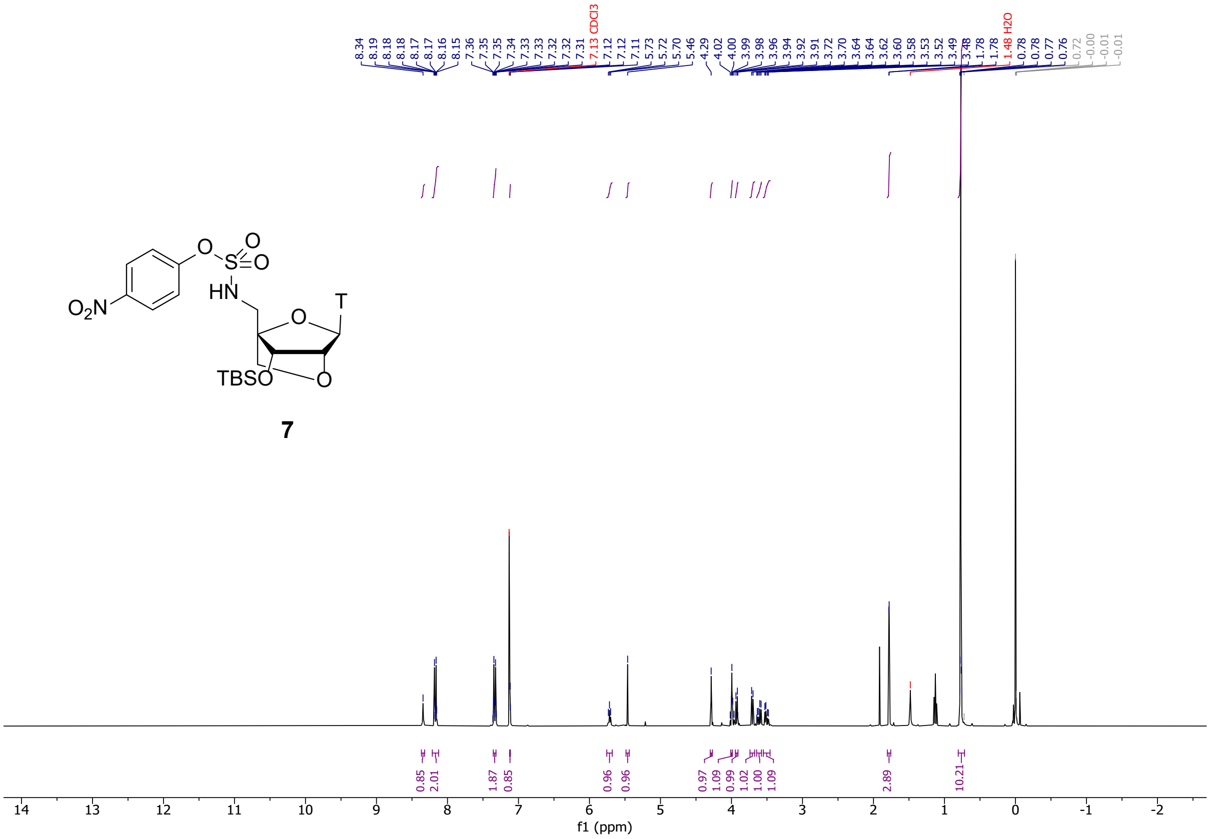

Supplementary Figure 46. ^{1}H NMR (400 MHz, $CDCl_3$) spectrum of compound **7.**

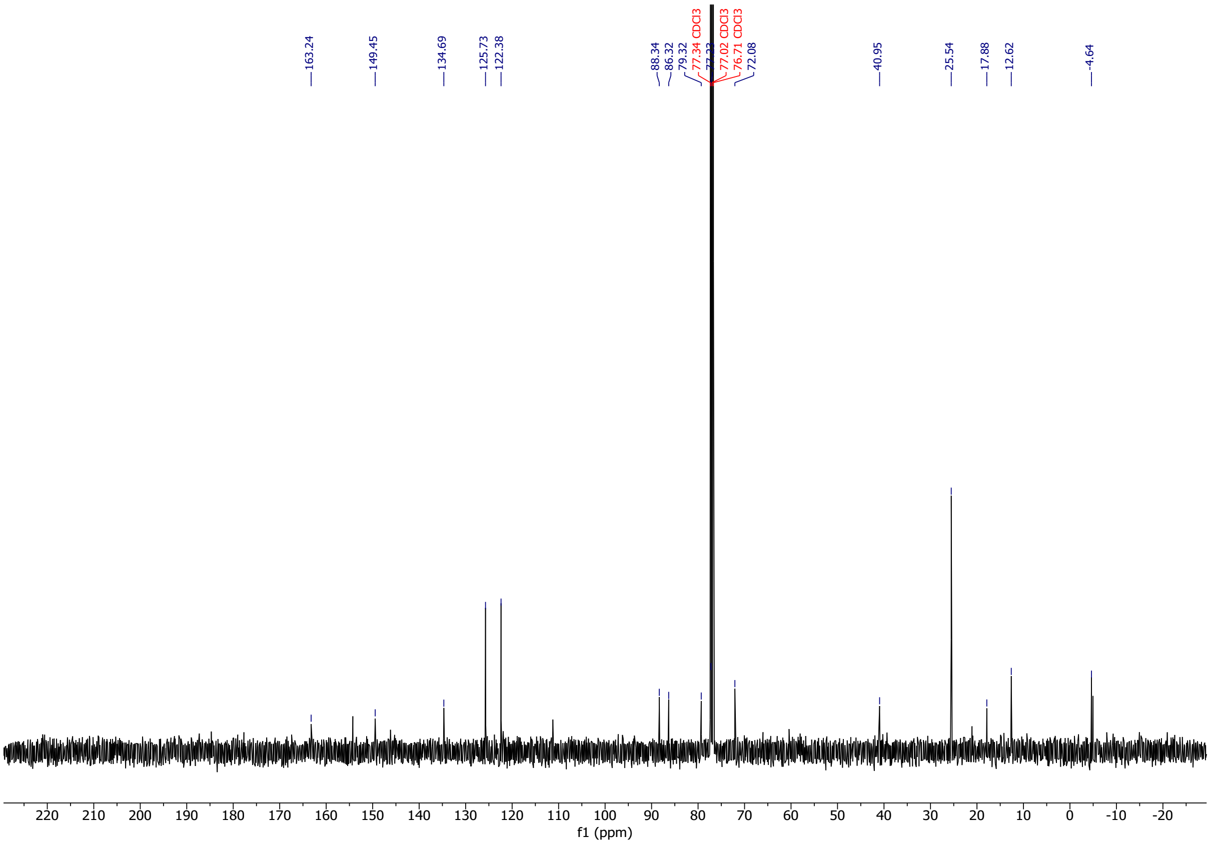

Supplementary Figure 47. ^{13}C NMR (101 MHz, $CDCl_3$) spectrum of compound **7.**

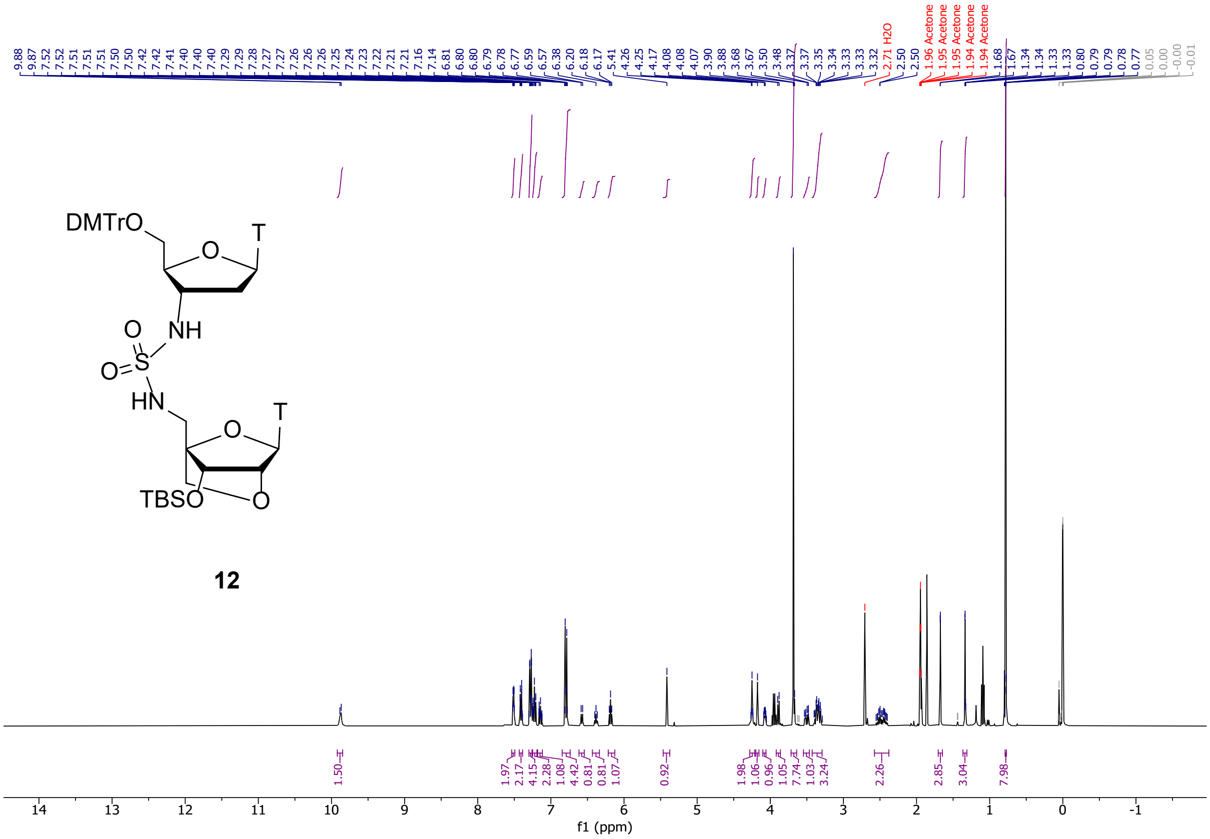

Supplementary Figure 48. ^{1}H NMR (400 MHz, deuteroacetone) spectrum of compound **12.**

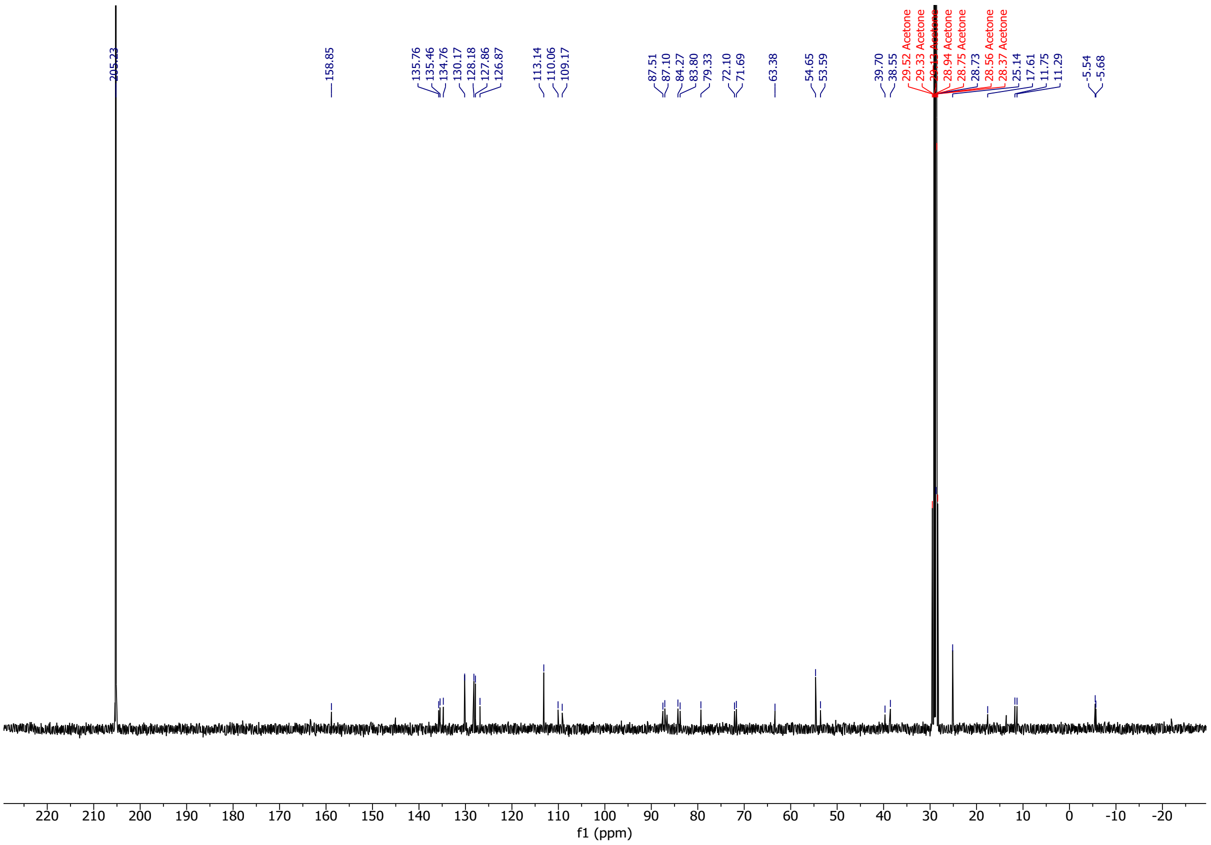

Supplementary Figure 49. ^{13}C NMR (101 MHz, deuteroacetone) spectrum of compound **12.**

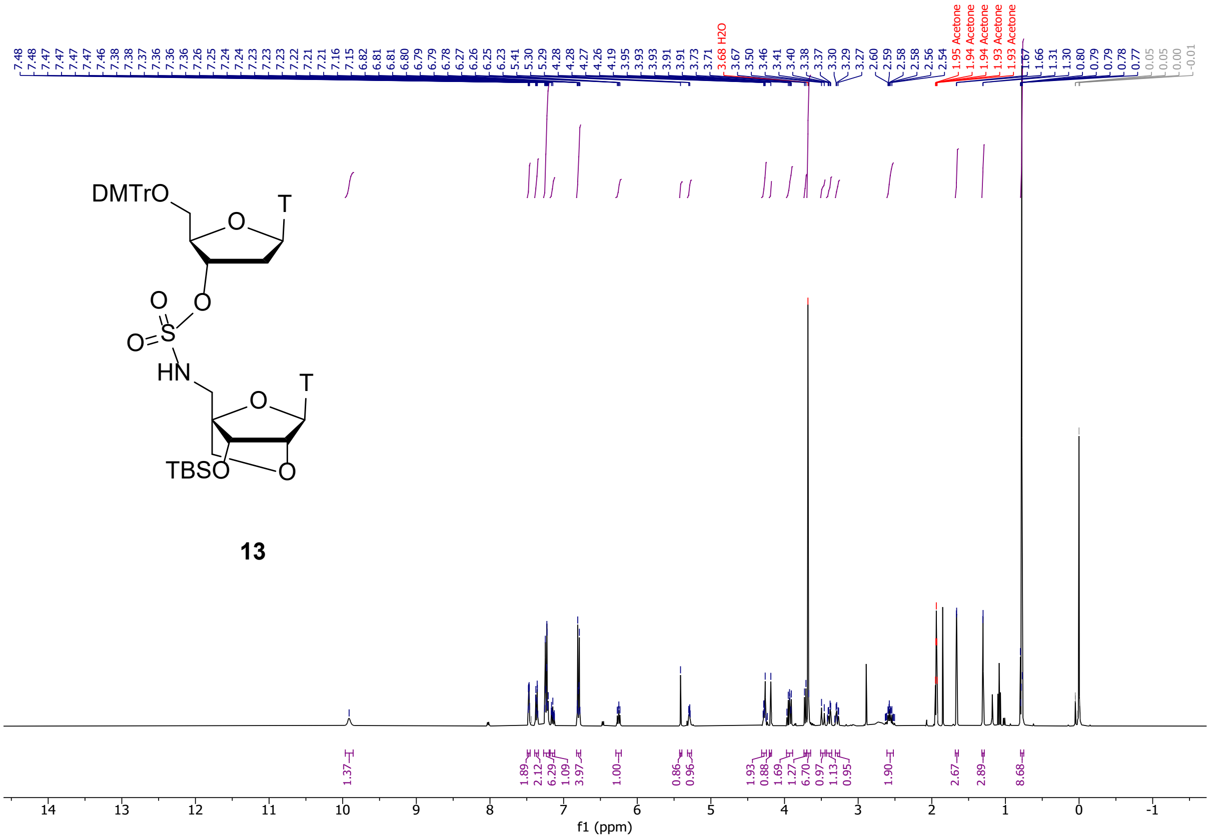

Supplementary Figure 50. ^{1}H NMR (400 MHz, deuteroacetone) spectrum of compound **13.**

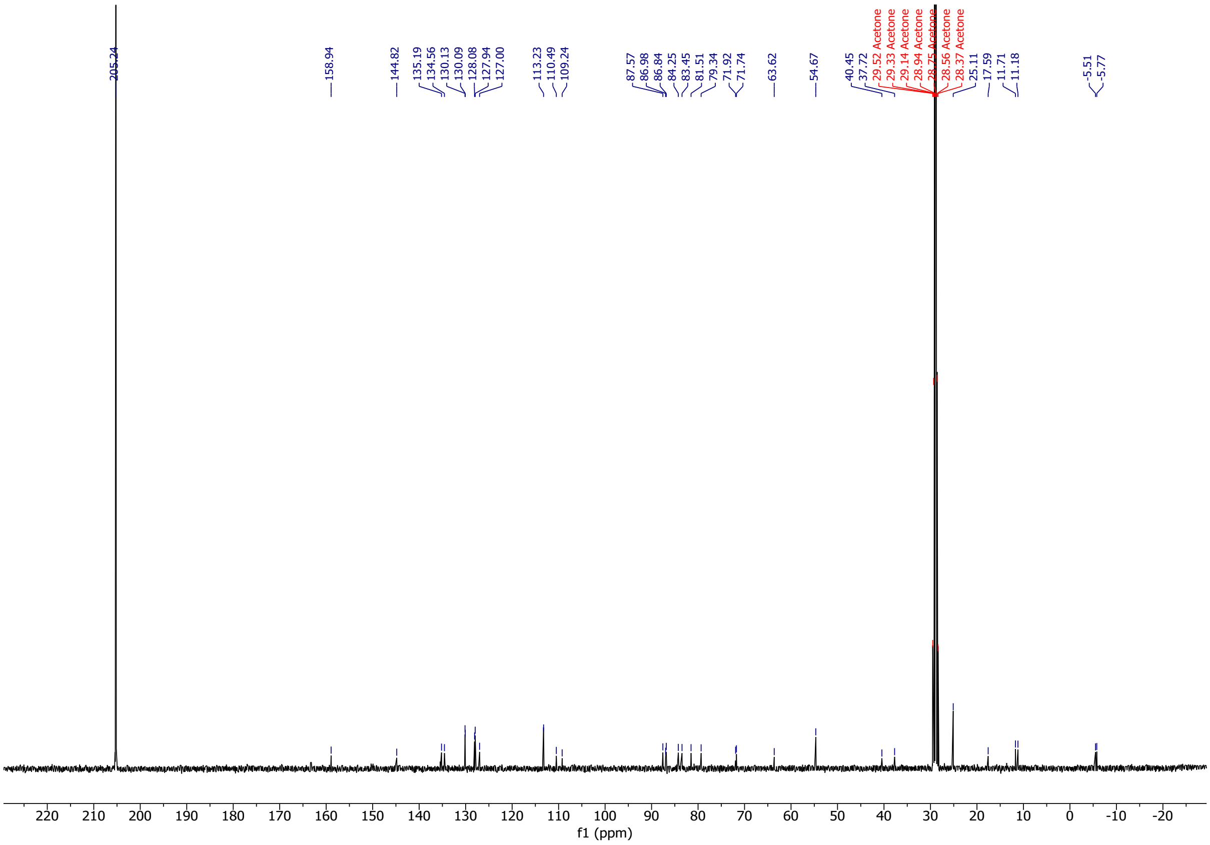

Supplementary Figure 51. ^{13}C NMR (101 MHz, deuteroacetone) spectrum of compound **13.**

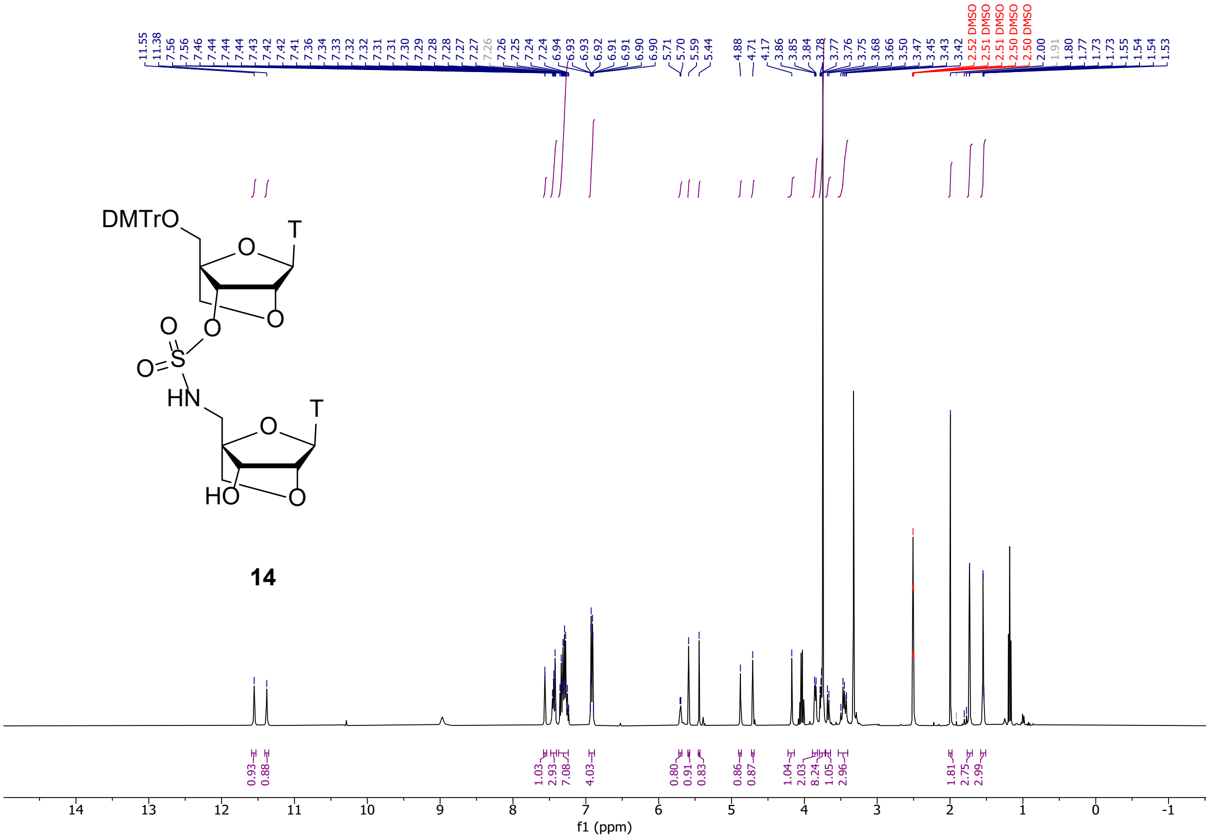

Supplementary Figure 52. ^{1}H NMR (400 MHz, DMSO-*d6*) spectrum of compound **14.**

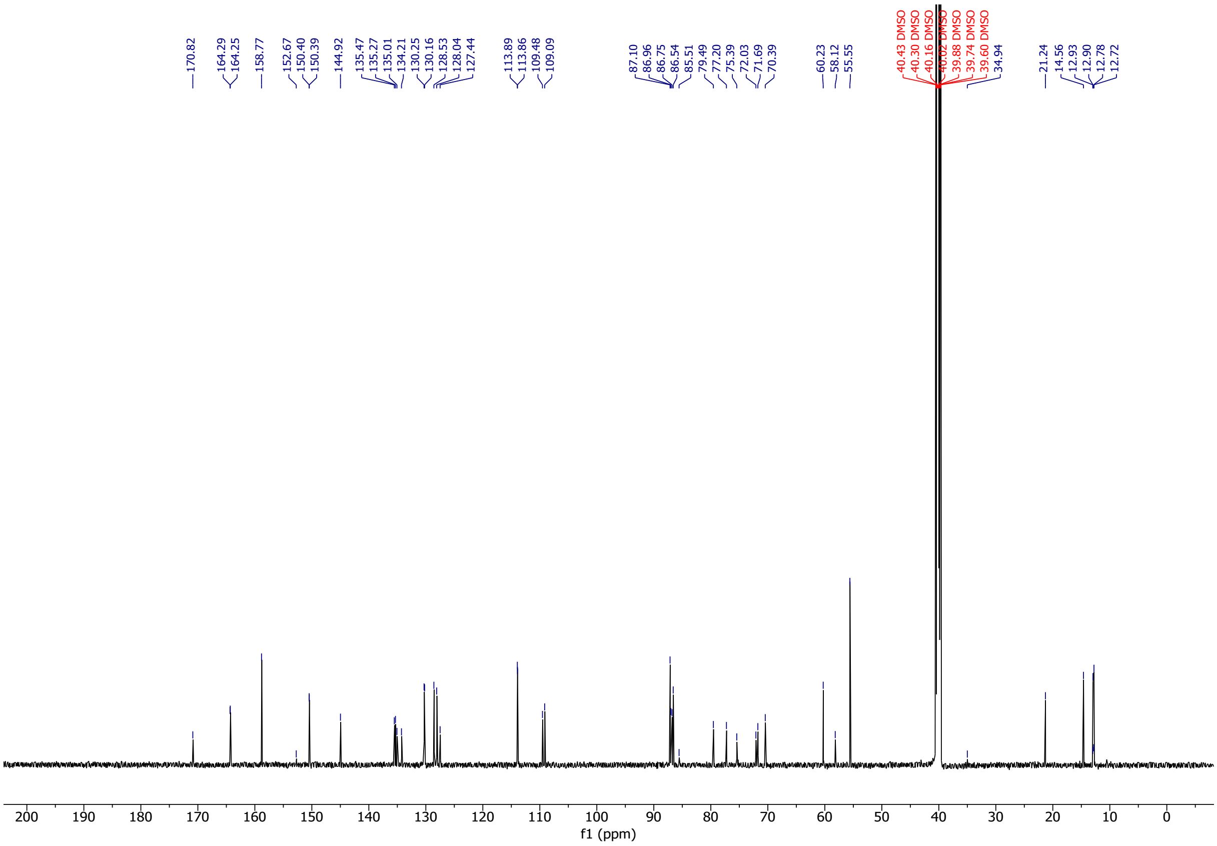

Supplementary Figure 53. ^{13}C NMR (151 MHz, DMSO-*d6*) spectrum of compound **14.**

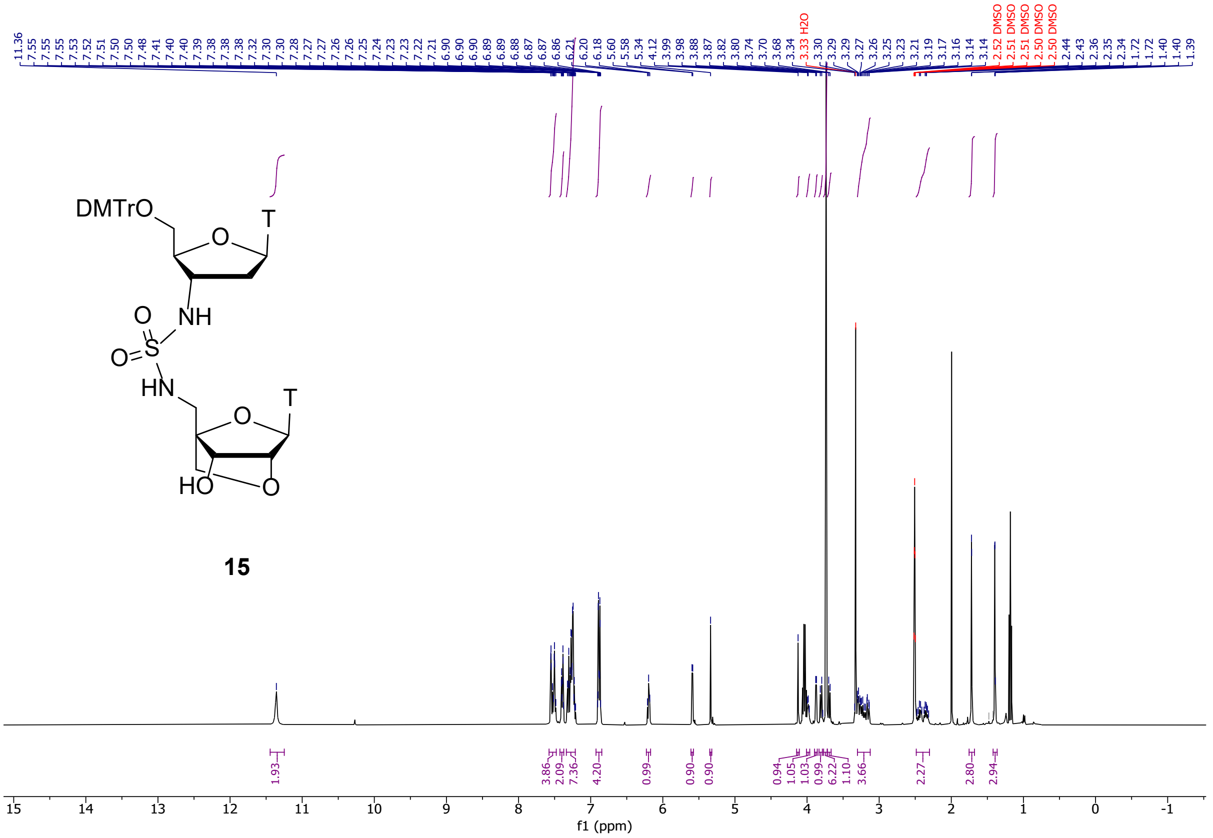

Supplementary Figure 54. ^{1}H NMR (400 MHz, DMSO-*d6*) spectrum of compound **15.**

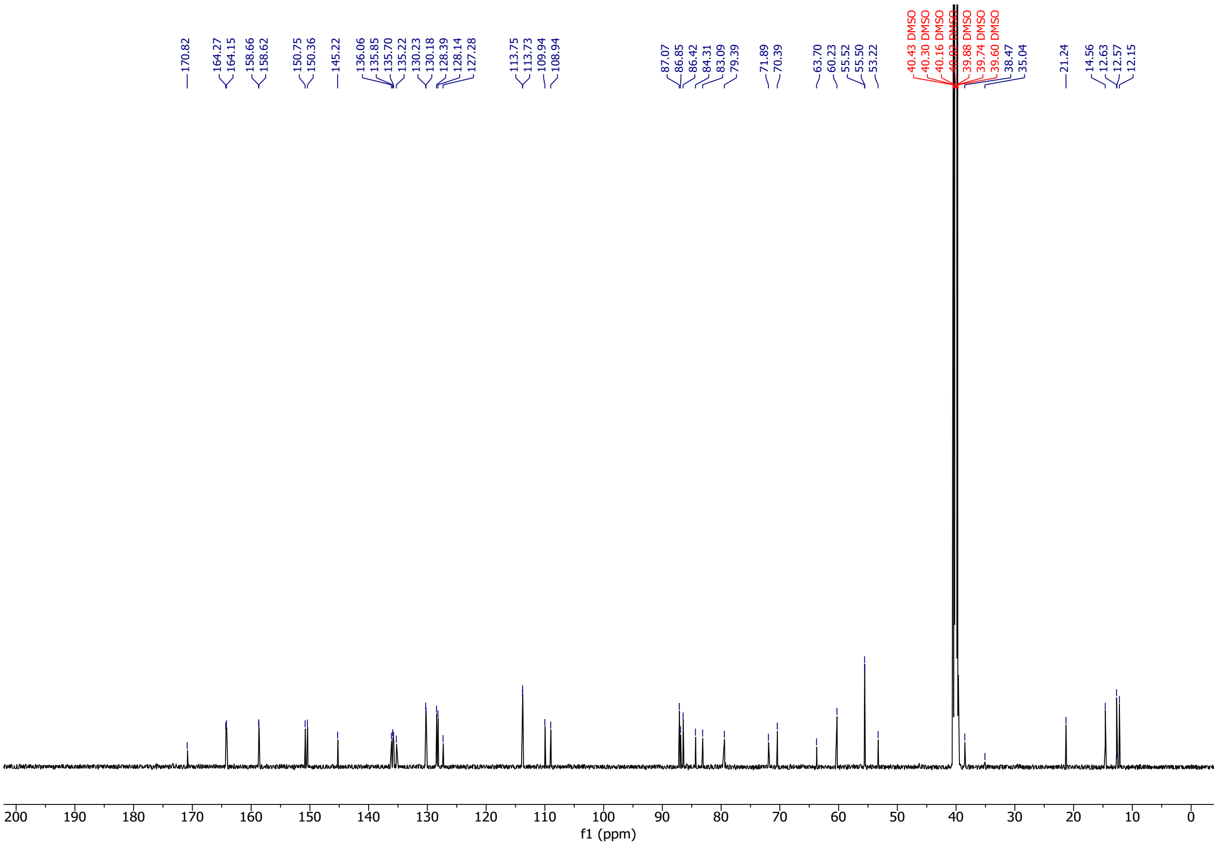

Supplementary Figure 55. ^{13}C NMR (151 MHz, DMSO-*d6*) spectrum of compound **15.**

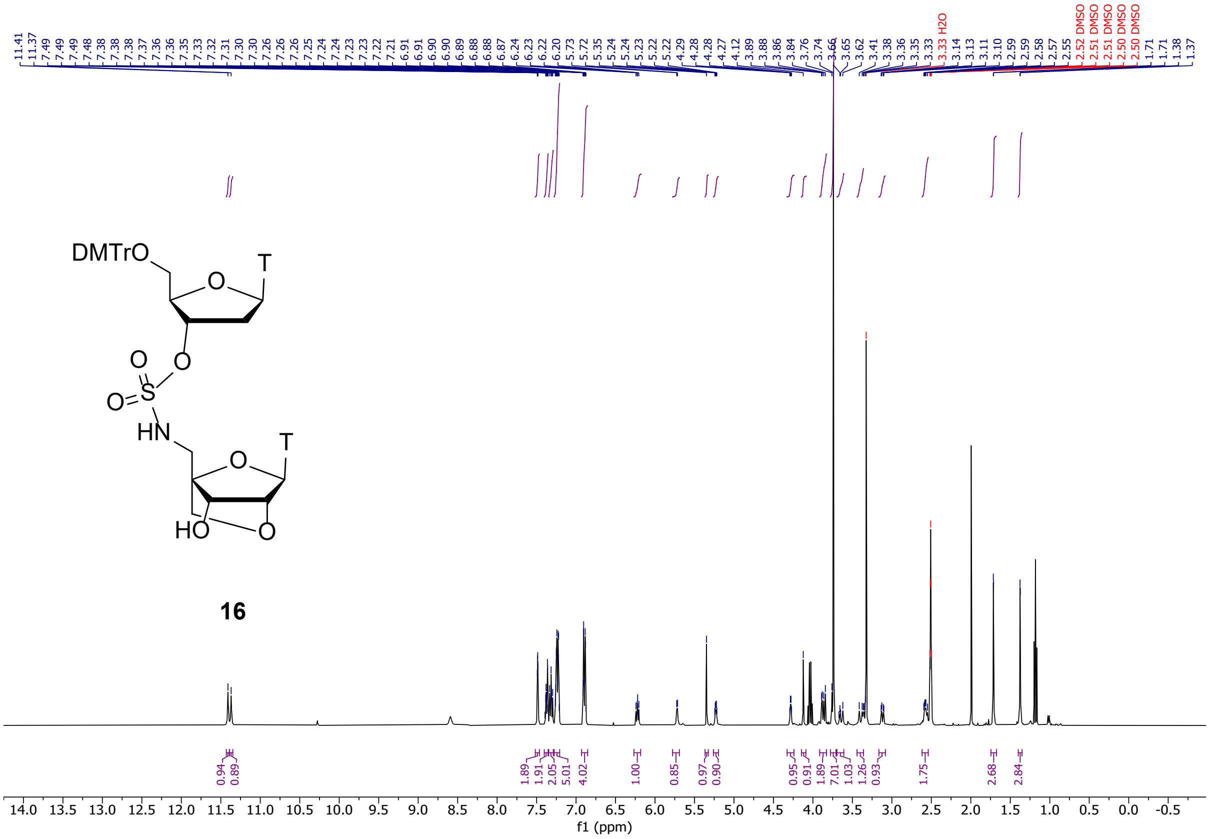

Supplementary Figure 56. ^{1}H NMR (400 MHz, DMSO-*d6*) spectrum of compound **16**

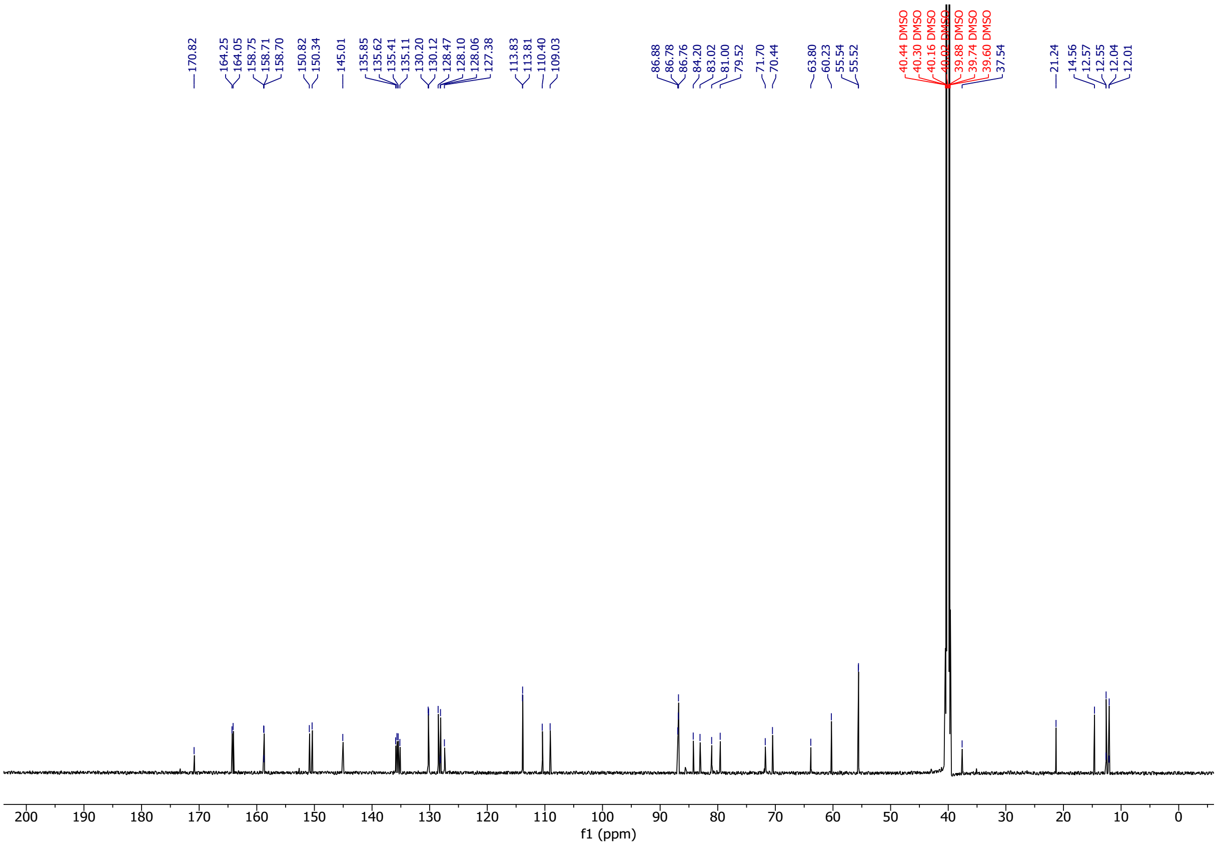

Supplementary Figure 57. ^{13}C NMR (151 MHz, DMSO-*d6*) spectrum of compound **16.**

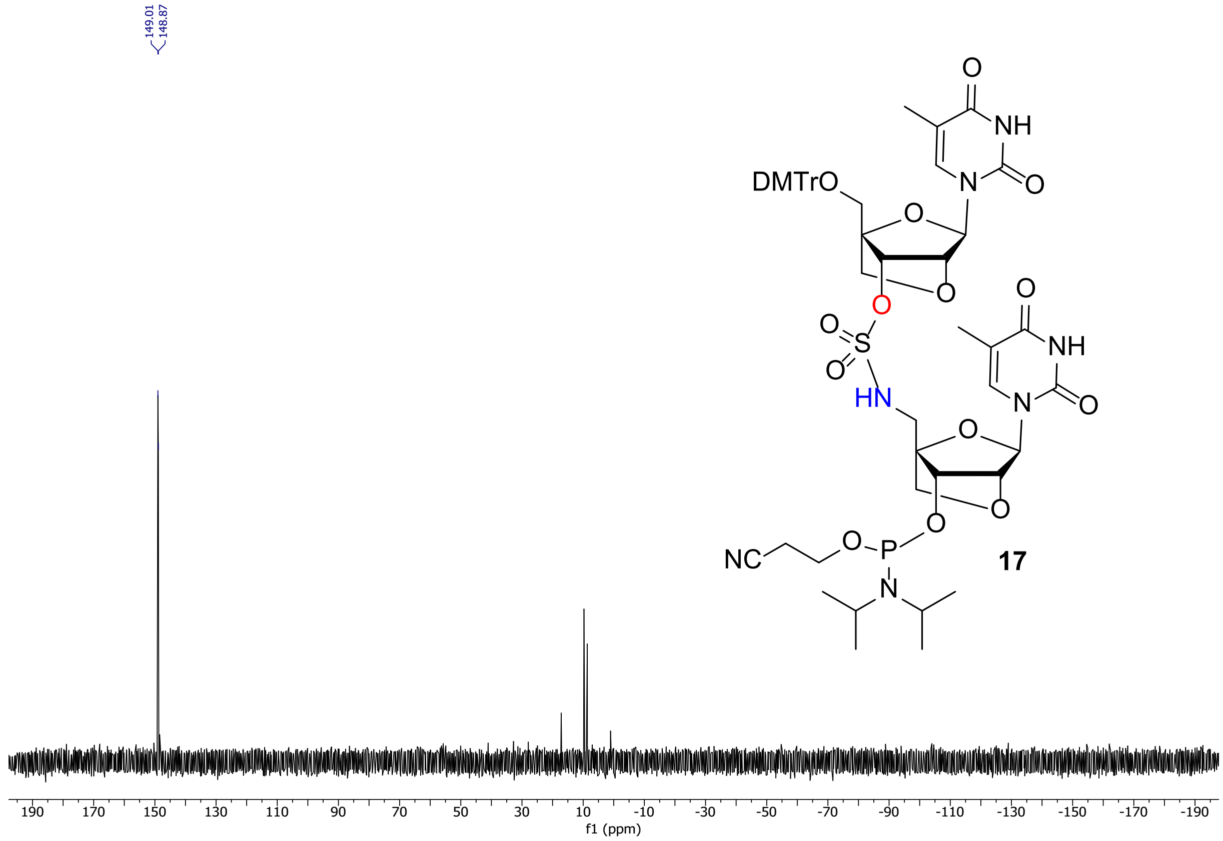

Supplementary Figure 58. ^{31}P NMR (162 MHz, CD_3CN) spectrum of compound **17.**

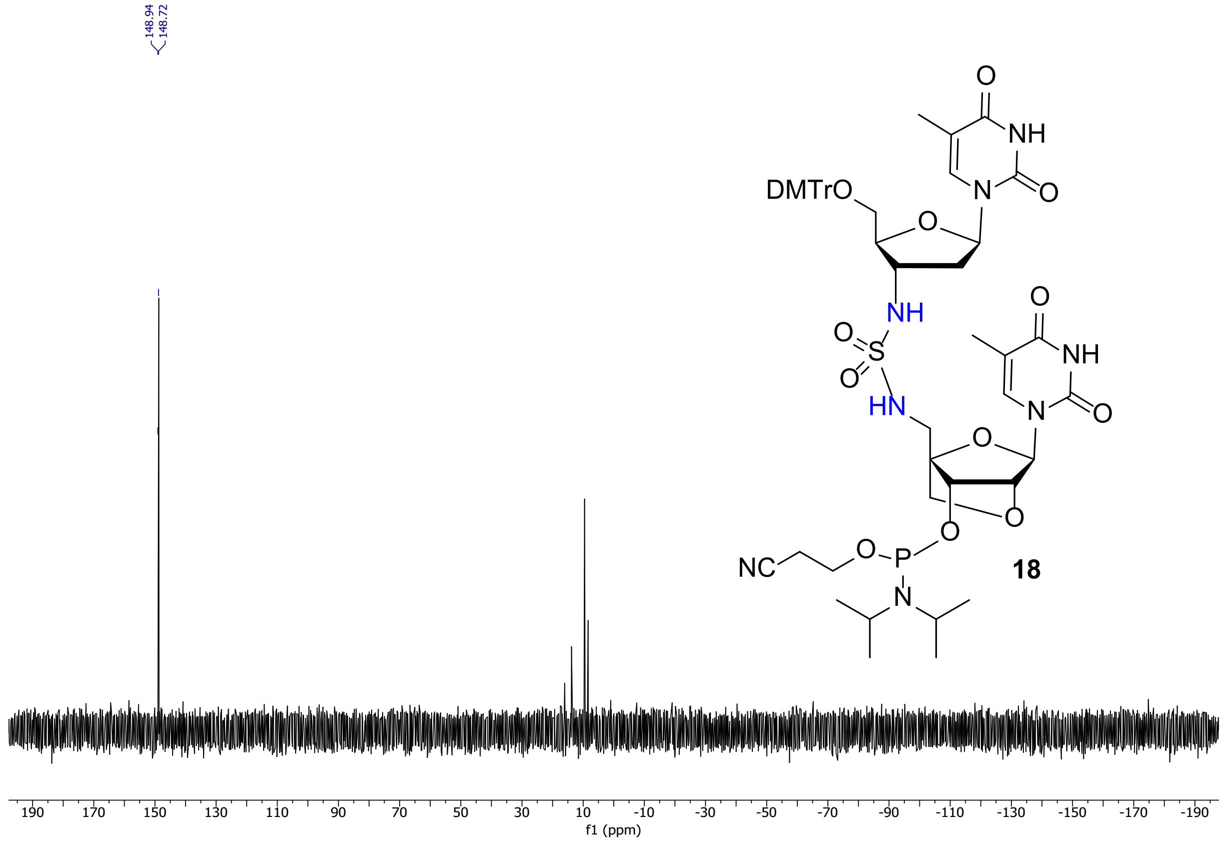

Supplementary Figure 59. ^{31}P NMR (162 MHz, CD_3CN) spectrum of compound **18.**

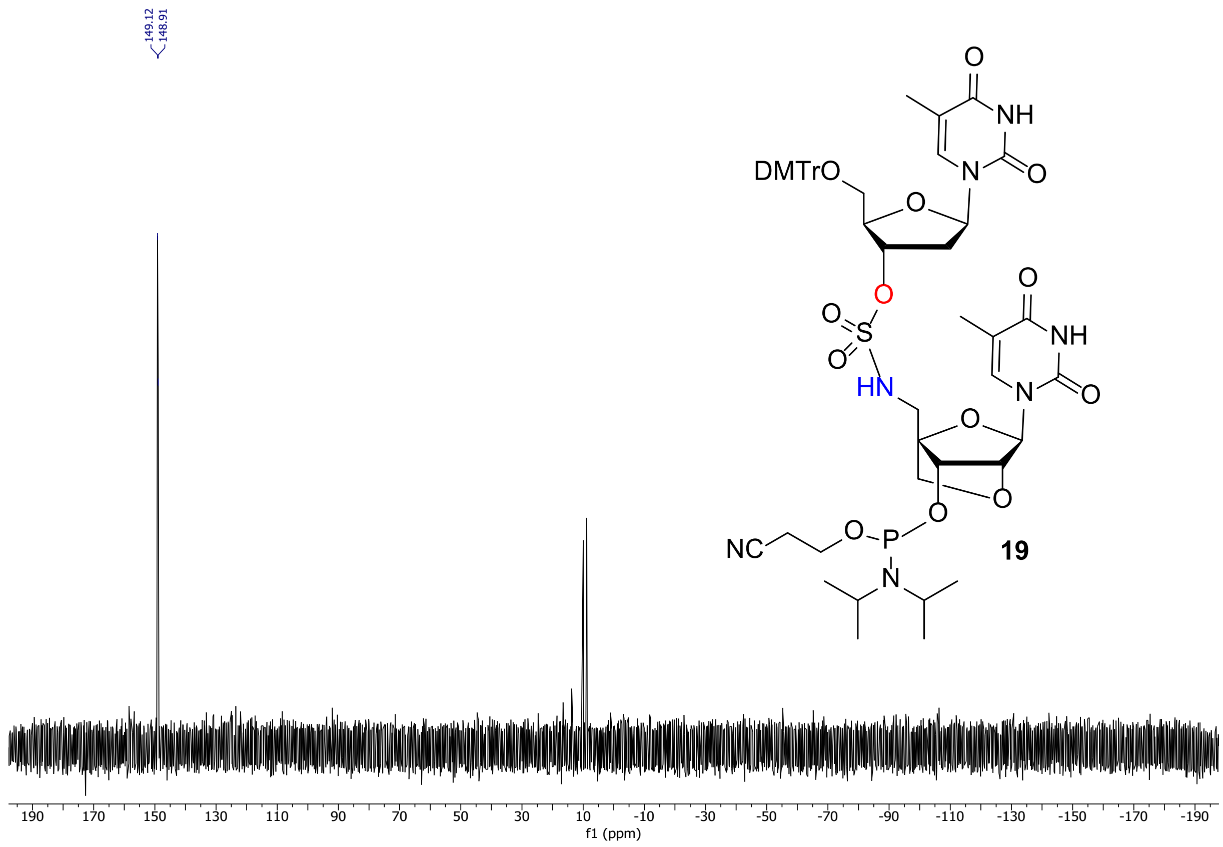

Supplementary Figure 60. ^{31}P NMR (162 MHz, CD_3CN) spectrum of compound **19.**
